# Syphilis infection prevalence in the Middle East and North Africa: a systematic review and meta-analysis

**DOI:** 10.1016/j.eclinm.2024.102746

**Published:** 2024-07-29

**Authors:** Mariam El-Jamal, Beyhan Annan, Alaa Al Tawil, Melissa Hamati, Sawsan Almukdad, Iman Fakih, Fatema Dabdoub, Eman Sharara, Muhammad S. Jamil, Ahmed S. Alaama, Joumana G. Hermez, Jane Rowley, Laith J. Abu-Raddad, Ghina R. Mumtaz

**Affiliations:** a Department of Epidemiology and Population Health, American University of Beirut, Beirut, Lebanon; b Interprofessional Education Office, QU Health, Qatar University, Doha, Qatar; c Department of Family and Community Medicine, University of Texas Southwestern Medical Center, Dallas, TX, USA; d Department of Communicable Diseases, HIV/Hepatitis/STIs Unit, World Health Organization Regional Office for the Eastern Mediterranean, Cairo, Egypt; e Department of Global HIV, Hepatitis, and Sexually Transmitted Infections Programmes, World Health Organization, Geneva, Switzerland; f Infectious Disease Epidemiology Group, Weill Cornell Medicine-Qatar, Cornell University, Qatar Foundation – Education City, Doha, Qatar; g World Health Organization Collaborating Centre for Disease Epidemiology Analytics on HIV/AIDS, Sexually Transmitted Infections, and Viral Hepatitis, Weill Cornell Medicine–Qatar, Cornell University, Qatar Foundation–Education City, Doha, Qatar; h Department of Population Health Sciences, Weill Cornell Medicine, Cornell University, New York, NY, USA; i Department of Public Health, College of Health Sciences, QU Health, Qatar University, Doha, Qatar; j College of Health and Life Sciences, Hamad bin Khalifa University, Doha, Qatar; k Center for Infectious Diseases Research, Faculty of Medicine, American University of Beirut, Beirut, Lebanon

**Keywords:** Syphilis, *Treponema pallidum*, Prevalence, Middle East and North Africa

## Abstract

**Background:**

Syphilis is a sexually transmitted infection (STI) that can be prevented and effectively treated; yet it continues to be a cause of morbidity and mortality worldwide. There is a limited understanding of the epidemiology of syphilis in the Middle East and North Africa (MENA) region.

**Methods:**

A systematic review conducted up to April 30, 2024 assessed the prevalence of syphilis and followed PRISMA guidelines, without language and date restrictions. Syphilis infection was categorized based on the diagnostic test type, distinguishing between current and lifetime infections. Pooled mean prevalence estimates were determined through random-effects meta-analyses. Random-effects meta-regression analyses were conducted to investigate sources of heterogeneity between studies and identify factors associated with syphilis prevalence.

**Findings:**

The review identified 643 studies based on close to 38 million syphilis tests in the 24 MENA countries. The pooled prevalence for probable current syphilis infection was 0.004% (95% CI: 0.001%–0.025%) among blood donors, 0.48% (95% CI: 0.22%–0.82%) in the general population (pregnant women and other general population groups), 2.76% (95% CI: 1.51%–4.35%) in populations at intermediate risk, 4.18% (95% CI: 2.08%–6.89%) among STI clinic attendees, 12.58% (95% CI: 8.45%–17.35%) among female sex workers, and 22.52% (95% CI: 12.73%–34.06%) among men who have sex with men and transgender people. Meta-regression analyses explained 62% of the prevalence variation and indicated a hierarchical pattern in prevalence by population group, higher prevalence among men, considerable subregional variability, and an annual decline of 3% in prevalence among general population groups and 8% among populations at high risk.

**Interpretation:**

Syphilis prevalence in MENA is comparable to global levels, emphasizing a considerable yet often overlooked disease burden with implications for reproductive health and social well-being. The observed rate of decline in syphilis prevalence and the current response fall short of the global targets. Action is required to control syphilis and mitigate its impact, especially in most affected populations.

**Funding:**

Qatar Research, Development, and Innovation Council (ARG01-0524-230273); 10.13039/100008982Qatar National Research Fund (NPRP grant number 9-040-3-008).

**Research in context**

**Evidence before this study**

Syphilis is a common, preventable, and treatable sexually transmitted infection (STI) caused by the bacterium *Treponema pallidum*. If left untreated, syphilis can lead to severe adverse conditions, including poor reproductive outcomes if transmitted vertically from mother to child, serious morbidity associated with advanced stages of the disease, reduced quality of life, and increased HIV transmission. The World Health Organization (WHO)'s 2022–2030 Global Health Sector Strategy on STIs aims to achieve a 90% reduction in syphilis incidence by 2030. Despite global reduction in prevalence over the past three decades, syphilis has remained endemic in low- and middle-income countries, while a surge in prevalence has been observed in several high-income countries in recent years. The epidemiology of syphilis is poorly understood in the Middle East and North Africa (MENA) where STIs have been historically neglected on national public health agendas. We searched PubMed, from database inception to April 30, 2024, with no language restrictions, using the terms “Syphilis”, “*Treponema pallidum*”, and “Review”, and found no systematic review covering the different at-risk populations for this infection in MENA.

**Added value of this study**

By searching various data sources and using rigorous methodologies, this study identified a large volume of data on syphilis prevalence in MENA, enabling diverse analyses and establishing a detailed understanding of the infection's epidemiology across various population groups. The prevalence of probable current syphilis infection in the general population was estimated at 0.48%, which is comparable to the global average and higher than expected given the sexually conservative norms of the region. Syphilis prevalence exhibited significant variability across MENA subregions and had substantially higher levels among populations at high risk, including men who have sex with men, transgender people, and female sex workers. Syphilis prevalence showed a decreasing trend over the last few decades, with a 3% annual decline among general populations and a more substantial 8% decline among populations at high risk.

**Implications of all the available evidence**

Syphilis prevalence in the MENA region aligns with global rates, indicating a significant, yet overlooked disease burden with implications for reproductive health and social well-being. The elevated prevalence of this treatable infection may, in part, be attributed to limited access to and underutilization of screening and treatment services for STIs. Although on the right track, the observed rates of decline in syphilis prevalence in the region are still much lower than the reductions needed to achieve the targets of the WHO's Global Health Sector Strategy on STIs. Urgent response and an inclusive public health agenda are needed to curb syphilis transmission and its associated disease burden, while addressing enduring challenges such as STI stigma, socio-cultural sensitivities, and effects of ongoing conflict and humanitarian crises. Critical steps involve the development of targeted, culturally sensitive, and gender-specific programs to expand prevention and treatment services. Specific strategies include increasing antenatal screening coverage across the region, integrating syphilis testing into testing for key populations, combining STI services with HIV and other health program areas, and promoting partner services.

## Introduction

Syphilis is a common, preventable, and treatable sexually transmitted infection (STI) caused by the spirochete *Treponema pallidum*—subspecies of *pallidum*.^1^^,^^2^ If left untreated, syphilis can lead to significant morbidity including poor reproductive outcomes, reduced quality of life, and increased HIV transmission.^1^^,^^3^^,^^4^ *T. pallidum* is transmitted through sex and vertically from mother to fetus during pregnancy or childbirth, leading to congenital syphilis, which is a major cause of fetal and neonatal morbidity and mortality globally despite being easily preventable.^5^^,^^6^ Although syphilis is typically only transmitted during early stages of the infection, up to two years after pathogen acquisition, its asymptomatic nature in a large proportion of cases complicates its control.^3^

The global annual incidence of syphilis is estimated by the World Health Organization (WHO) at 7.1 million cases in 2020, with most occurring in low- and middle-income countries (LMIC).^7^ Despite significant global reductions in syphilis prevalence in the 1990s in part due to syndromic management of STIs, reductions in risk behavior prompted by the HIV pandemic, and AIDS-related deaths,^8^, ^9^, ^10^, ^11^, ^12^ syphilis has remained endemic in LMIC.^3^ Within the last few years, an increase in syphilis prevalence, including congenital syphilis, has been documented in several high-income countries, accompanying budget reductions in previously successful STI programs and public health surveillance systems.^11^^,^^13^^,^^14^ Increasing syphilis rates have been notably documented among men who have sex with men (MSM), possibly due to decreased use of protective measures or increased sexual risk behaviour following the introduction of antiretroviral therapy (ART)^11^^,^^15^ and the more recent scale-up of pre-exposure prophylaxis (PrEP) for HIV.^16^^,^^17^

The WHO's 2022–2030 Global Health Sector Strategy aims to achieve a 90% reduction in the number of new syphilis cases by 2030 and to decrease the incidence of congenital syphilis to below 50 cases per 100,000 live births by 2030.^18^ One of the strategic directions to reach the global target is to “generate and use data to drive decisions for action”,^18^ which implies a better characterization of the epidemiology of these infections. Yet, there remain important gaps in our understanding of the true burden of syphilis, particularly in LMIC due to limited screening programs, inadequate surveillance, and the scarcity of population-based studies.^11^^,^^15^ The epidemiology of syphilis is particularly poorly understood in the Middle East and North Africa (MENA) where STIs have been historically neglected on national public health agendas, leading to limited capacity for surveillance as well as poor prevention, screening, and treatment programs.^19^^,^^20^ For example, less than half of MENA countries report having a national strategy plan for STI prevention and control, and only about half report having policy for routinely screening pregnant women for syphilis.^21^

While the MENA region has been represented in earlier global analyses of syphilis data^7^^,^^12^^,^^22^ and in two reviews of select population groups,^23^^,^^24^ this is to our knowledge the first systematic review and meta-analytics that present an analysis of the epidemiology of syphilis across all population groups in MENA. The objectives of the present study are to 1) systematically compile and synthesize evidence on the prevalence of syphilis infection in MENA, 2) estimate the pooled mean prevalence of syphilis infection in various population groups at subregional and regional levels, and 3) explore sources of between-study heterogeneity, factors associated with syphilis prevalence, as well as temporal trends in syphilis prevalence in the region.

## Methods

### Search strategy and selection criteria

We conducted a systematic review on the prevalence of syphilis in MENA informed by the Cochrane Collaboration guidelines.^25^ Findings were reported based on the Preferred Reporting Items for Systematic Reviews and Meta-analyses (PRISMA) guidelines.^26^^,^^27^ Our study included 24 countries—Afghanistan, Algeria, Bahrain, Djibouti, Egypt, Iran, Iraq, Jordan, Kuwait, Lebanon, Libya, Morocco, Oman, Pakistan, Palestine, Qatar, Saudi Arabia, Somalia, Sudan, recently independent South Sudan, Syria, Tunisia, United Arab Emirates, and Yemen—based on the combined MENA definitions of the WHO, United Nations Programme on HIV/AIDS (UNAIDS), and the World Bank,^28^ and is consistent with existing convention in such systematic reviews.^20^^,^^29^, ^30^, ^31^, ^32^, ^33^, ^34^, ^35^, ^36^ These countries share specific similarities, whether historical, socio-cultural, or linguistic; and are conventionally included together as part of HIV/STI programming for the region.

Countries were classified into the following six subregions: Fertile Crescent (Egypt, Iraq, Jordan, Lebanon, Palestine, Syria), North Africa (Algeria, Libya, Morocco, Tunisia), the Gulf (Bahrain, Kuwait, Oman, Qatar, Saudi Arabia, and United Arab Emirates), Horn of Africa (Djibouti, Somalia, Sudan, South Sudan, and Yemen), Pakistan (classified alone because it has the largest population size and contributed the largest number of syphilis prevalence measures), and Eastern MENA (Afghanistan and Iran).

We searched PubMed and Embase up to April 30, 2024, with no restriction on language or date of publication, using a combination of MESH/Emtree and text terms for syphilis (example “*Treponema pallidum*”, “syphilis”) and names of countries and territories included in the study (example “Eastern Mediterranean”, “Oman”). We did not include a ‘prevalence’ term to avoid restricting the search and missing potentially relevant records. The detailed search criteria can be found in Table S1. We also searched UNAIDS database of integrated biobehavioral surveillance surveys (IBBSS), national data as part of the WHO Global Health Observatory data repository,^37^ including UNAIDS SPECTRUM database, up to December 2022, and abstracts of international conferences for the years 2010–2021 (International AIDS Conferences^38^; International AIDS Society Conferences on HIV Pathogenesis, Treatment and Prevention^39^; and International Society for Sexually Transmitted Disease Research^40^).

PubMed and Embase search results were combined in EndNote (Thomson Reuters, USA) where duplicates were identified and excluded. Titles and abstracts were then screened independently by two authors (MEJ, BA, AAT, MH, IF, and SA). The full-texts of potentially relevant citations were retrieved and screened for eligibility. Any report with primary data on the prevalence of syphilis in any of the 24 MENA countries was considered eligible for inclusion in the review. Qualitative studies, editorials, letters to editors, commentaries, and reviews were excluded. Although excluded, relevant reviews were screened to make sure no studies were missed. Bibliographies of all included reports were also hand searched for additional reports. Prevalence measures extracted from UNAIDS IBBSS database, the WHO Global Health Observatory, and from international conferences were compared with records identified through PubMed and Embase to avoid duplication. Discrepancies between independent reviewers were resolved by GRM.

### Data extraction

The data was double extracted independently by MEJ, BA, AAT, MH, FD, IF, and ES using a pre-designed computerized data extraction sheet. Discrepancies were settled either by consensus, including also GRM and LJA, or by contacting the authors. Full-texts of articles in French were extracted by French-speaking authors (GRM and MH) (n = 9). Data from one article in Farsi was extracted from the English abstract. All other articles were in English. Both overall and stratified syphilis prevalence rates were extracted, when applicable.

### Syphilis diagnostics and categorization

Syphilis is a complex and challenging infection to diagnose with currently available diagnostics. Since it is not possible to culture *T. pallidum* on artificial media, serological tests remain the mainstay of syphilis diagnosis.^41^ Typically, the serologic diagnosis of syphilis requires the detection of both treponemal and non-treponemal antibodies.^41^, ^42^, ^43^ Yet, in most circumstances, it is challenging to distinguish current from previously treated infections based on a set of serologic test results alone, without review of past serologic test results, patient history, or physical examination results.^41^^,^^42^ This comprehensive assessment is, however, not typically available in most studies of syphilis prevalence, where only treponemal and/or non-treponemal test results are reported and used to diagnose syphilis. Therefore, in this review, syphilis prevalence measures have been categorized based on the results of the serological test(s) reported in eligible studies.

Despite the complexities and limitations in interpreting these serological test results, each category was assigned a general interpretation, for epidemiological relevance. As such, the following categories, based on different serological test results combinations, were used for infection type: 1) *probable current (active) syphilis infection* (positive both treponemal and non-treponemal tests), 2) *possible current infection, unspecified* (positive non-treponemal test, irrespective of treponemal test result if done), 3) *lifetime syphilis infection* (positive treponemal test, irrespective of non-treponemal test result if done), and 4) *unclear* if the diagnostic test used was not reported or not adequately described. These categories, their description, interpretation in terms of syphilis diagnosis, and limitations in this interpretation are further described in Table S2.

Whenever available in a report, we extracted all prevalence measures corresponding to these infection/serological test categories. Consequently, one report could contribute prevalence measures for more than one syphilis infection type. This approach enabled us to utilize the maximum information possible from the testing done in each study.

Ideally, *probable current syphilis infection* diagnosis (category 1) would entail non-treponemal antibody titers to disentangle individuals with a past, adequately treated infection. However, this information was rarely reported in the studies we identified. Most countries of the MENA region are low- or middle-income countries with limited resources and inadequate laboratory capacity where qualitative non-treponemal tests are most commonly used. It was therefore not possible to include antibodies titers as part of the classification of diagnostics in our study. A positive result on both non-treponemal and treponemal tests without titers is considered, as per WHO screening guidelines, the indicator of choice for *probable current syphilis infection*,^42^^,^^43^ and is the definition used in previous global systematic reviews.^12^^,^^22^^,^^42^^,^^44^

### Bias and precision assessment

We qualified the quality of individual syphilis prevalence measures by assessing their risk of bias (ROB) and precision as informed by the Cochrane Collaboration approach.^45^ The results of these assessments were incorporated into meta-regression analyses (see Results section) to investigate the influence of study quality on the observed syphilis prevalence, using established methodologies.^20^^,^^23^^,^^30^^,^^46^

Each prevalence measure was classified as having high, low, or unclear ROB on each of two domains. The first domain is sampling methodology: a prevalence measure was classified as having high ROB if it used non-probability-based sampling (e.g., convenience, snow-balling, and others…), low ROB if it used probability-based sampling (e.g., simple random sampling, time-location sampling, respondent-driven sampling given that the recruitment process is implemented in a manner that allows for the calculation of selection probabilities,^47^ and others), and unclear ROB if the sampling methodology was not reported or is unclear. The second domain is syphilis ascertainment: A prevalence measure was classified as having low risk of bias on this domain if the biological assay for diagnosing the infection was explicitly indicated and described, and unclear ROB if otherwise.

A syphilis prevalence measure was considered to have “high precision” if the number of participants tested was ≥200 for populations at high risk and STI clinic attendees, and ≥500 for all other population groups. These sample sizes were considered to yield sufficiently narrow 95% confidence intervals (CIs) around the syphilis prevalence. For a median syphilis prevalence of 4.8% among populations at high risk and STI clinic attendees (see Results section), a sample size of 200 implies a 95% CI of 2.4%–9.0%; and for a median syphilis prevalence of 0.7% for all other populations, a sample size of 500 implies a 95% CI of 0.1%–1.7%.

Publication bias in meta-analyses was assessed, stratified by population type, using Doi plots along with the Luis Furuya-Kanamori (LFK) index whenever the number of pooled measures exceeded three.^48^ An asymmetrical Doi plot indicated potential publication bias; the spread of the prevalence measures may not be due to chance alone.^48^ An LFK index value exceeding ±1 was considered indicative of the presence of publication bias.^48^

### Statistical analysis

Meta-analyses were conducted to estimate the pooled mean prevalence of syphilis and corresponding 95% CIs for each population group and infection type. Population groups were classified based on the risk of exposure to syphilis (Panel 1), following established approaches for classifying populations based on risk of exposure to STIs.^20^^,^^30^^,^^46^^,^^49^ In additional analyses, the categories “Pregnant women” and “Other general population groups” were combined together to provide overall estimates for “The general population”.

*Panel 1* **Population group classification as per established approaches for classifying populations based on risk of exposure to sexually transmitted infections.20,30,46,49**

**Table:** 

Population	Description	Population groups included
Blood donors (populations at very low risk)	Individuals who voluntarily donate blood for medical purposes. Blood donors are likely to be a healthy population with low prevalence of risk factors for syphilis infection.	All types of blood donors including volunteer, family, and replacement donors.
Pregnant women (populations at low risk)	Women at any stage of pregnancy or in the post-partum period.	Pregnant women, antenatal clinic attendees, women in delivery wards, post-natal clinic attendees.
Other general populations–excluding blood donors and pregnant women (populations at low risk)	General population groups, other than blood donors and pregnant women, who are generally at low risk of exposure to syphilis infection.	Students, patients at primary health care centers, and others.
Populations at intermediate risk	Populations who presumably might have some sexual contacts with populations engaging in high sexual risk behavior, and have therefore a higher risk of exposure to syphilis infection than the general population.	Incarcerated people, homeless people, people who inject drugs, people living with HIV, truck drivers, clients of sex workers, military personnel, migrants, and others.
Populations at high risk	Populations at high risk of exposure to syphilis as a consequence of specific sexual risk behaviors.	Female sex workers, men who have sex with men^a^, male sex workers, and transgender people^b^.
STI clinic attendees	Persons seeking consultation at STI clinics, whether asymptomatic or with clinical manifestations related to syphilis infection or other STIs, or suspected of having a syphilis infection or other STIs.	Individuals seeking medical consultation at STI clinics or dermatovenereal clinics. Individuals with genital ulcerative lesions or macular rash, women with vaginal discharge, and individuals with venereal diseases.
Special clinical populations	Various clinical populations that are not classified into the above categories, some of whom might be at risk of parenteral transmission.	Individuals with hemophilia, individuals undergoing transplant, blood donors with transfusion transmissible infections, individuals with end-stage renal disease, individuals with tuberculosis, and others.

STI: Sexually transmitted infection.

a Men who have sex with men are defined as biological males who engage in same-sex sexual activities, specifically anal sex, regardless of their gender, sexual, social, or cultural identity. They may include bisexual men who have sex with both men and women.

b All transgender populations in studies included in the systematic review were biological males with a female gender identity (transwomen). No studies included transmen.

To account for sampling variation and capture true heterogeneity, estimates for all population types except blood donors were pooled using Dersimonian-Laird random-effects models, with inverse-variance weighting.^50^^,^^51^ Variances were stabilized using Freeman-Tukey type arcsine square-root transformation.^50^^,^^52^ For blood donor meta-analyses, we used generalized linear mixed models with logit transformation, as the back-transformation of the Freeman-Tukey double arcsine transformation can produce misleading results when pooling prevalence measures with a very large sample and a small number of events, as is typically the case for blood donor data.^53^ Generalized linear mixed models, which account for the binomial structure of the data and avoid the generic inverse variance method, are recommended as alternative methods for pooling single proportions in such cases.^53^

When available, stratified syphilis prevalence measures were used in the meta-analyses rather than the overall measure. When more than one stratification was reported, we used only one stratification based on a pre-defined sequential order that prioritizes test modality followed by population type, year of data collection, sex at birth, collection site, city, and age. This approach ensured that no overlapping prevalence measures from a single report were used. Reports published before 1970 often had unclear reported methodology and outlier data points, and hence were all excluded from the meta-analyses. Meta-analyses were conducted when at least three prevalence measures were available. The I^2^ heterogeneity metric was computed to estimate between-study variation that is due to real differences in effect size (syphilis prevalence) rather than chance.^54^^,^^55^ Pooled prevalence measures and their 95% CI were reported to two decimal places. One additional decimal place was included when any of the rounded estimates (prevalence measure or any of the CI bounds) was 0.00% for greater precision. Meta-analyses were conducted using the *meta* package^56^ in R version 4.3.2.^57^

Random-effects meta-regression analyses were conducted to investigate sources of between study heterogeneity and identify factors associated with syphilis prevalence. Population-related and study methodology-related independent variables were considered *a priori*. Population characteristics included: population type or sub-type, subregion, mid-year of data collection (modeled separately as a continuous and categorical variable to explore the trend of syphilis prevalence over time), and sex at birth. Missing mid-year of data collection was imputed by subtracting three years from the year of publication–the average difference between year of publication and mid-year of data collection for all reports with data on both dates. Study methodology characteristics included infection type (as classified above), sampling methodology (probability versus non-probability based), syphilis ascertainment (adequately described versus not), and precision (low versus high). Three main regression models were conducted: one for all population groups; one for only blood donors, pregnant women, and other general population groups; and one for only populations at high risk.

Univariable and multivariable meta-regression models were conducted using the log-transformed syphilis prevalence and corresponding variance. Crude and adjusted risk ratios and corresponding 95% CIs were reported. Factors associated with syphilis prevalence at p ≤ 0.20 in the univariable analyses were included in the multivariable analysis. Factors with p ≤ 0.05 in the multivariable model were considered as significant predictors of syphilis prevalence. Meta-regression analyses were conducted using Stata/SE v.14 using the *metareg* command.^58^

### Role of the funding source

The funders of the study had no role in study design, data collection, data analysis, data interpretation, or writing of the report.

## Results

### Search results and scope of evidence

**Fig. 1:**
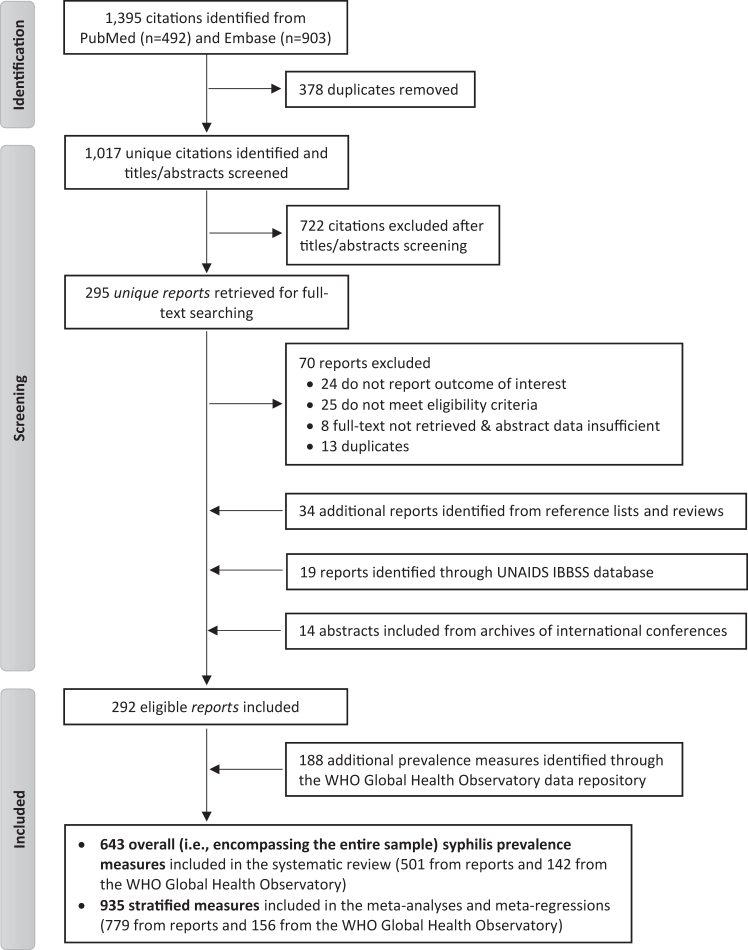
PRISMA flowchart depicting the study selection process for the systematic review of syphilis in the Middle East and North Africa. IBBSS: integrated biobehavioral surveillance survey, UNAIDS: United Nations Programme on HIV/AIDS, WHO: World Health Organization.

As depicted in the PRISMA flowchart (Fig. 1), the search identified a total of 1395 citations: 492 through PubMed and 903 through Embase. Of these citations, 295 unique reports were retrieved for full-text screening after removing duplicates and screening titles and abstracts. Upon screening full-texts, 70 reports were found to be non-eligible and were excluded. Additional eligible reports were identified through the search of UNAIDS' IBBSS database (n = 19), international conferences abstracts (n = 14), and hand-searching bibliographies of relevant reports and reviews (n = 34), leading to a total of 292 eligible reports included in the systematic review. In addition, 188 syphilis prevalence measures were extracted from the WHO Global Health Observatory data repository. In sum, the search retrieved, in total, 643 overall (i.e., encompassing the entire sample) prevalence measures (referred to as “studies” hereafter, individually described in Tables S3–S11) that yielded 957 stratified prevalence measures. From the latter, 22 measures from five reports published before 1970 were excluded from further analysis (but these studies are still described in Table S4 and Table S8) because of inconsistent methodologies and outlier data points. As such, a total of 935 stratified syphilis prevalence measures were included in the meta-analyses and meta-regressions. A visual representation of these 935 prevalence measures over time can be found in Figure S12.

There were close to 38 million syphilis test results reported across all population groups in the 24 MENA countries. Pakistan alone contributed 19.4% of all studies included in the systematic review, almost as much as the remaining countries in Eastern MENA (Afghanistan and Iran) combined, and the Horn of Africa contributed 25.7% of studies. Countries in the Gulf and North Africa contributed each about 14%, whereas the Fertile Crescent countries had the smallest number of data points (n = 57, 8.9%) (Table S13). Studies span a wide timeframe, with the earliest published in 1950. The majority of studies (84%) were conducted after the year 2000 and 60% after 2010 (Table S13). The most recent study for each population group was conducted between 2016 and 2023; the median mid-year of data collection was more recent for blood donors (2013), pregnant women (2012), and populations at high risk (2012), compared with the other population groups (Table S14, Figure S15). Out of all studies, 28.1% assessed probable current syphilis infection, 14.0% assessed possible current unspecified infection, 33.9% assessed lifetime syphilis infection, and 24.0% had an unknown or unclear time of infection (Table S13).

### Bias and precision assessment

Table S16 summarizes the precision and risk of bias assessments of syphilis prevalence studies. Two-thirds (65.2%) of prevalence measures were considered to have high precision based on the number of individuals tested. While only 27.1% of studies used probability-based sampling and hence were qualified as having low ROB on this domain, probability-based sampling (mostly respondent-driven sampling) was used in 60.0% of studies on populations at high risk, indicating low ROB in sampling these hard-to-reach populations. The type of assay used to detect syphilis was clearly described in 76.0% of studies, and hence these had low ROB on this domain. Overall, the majority of prevalence measures (80.4%) had low ROB on at least one of the two quality domains and 22.7% had low ROB on both domains (Table S16).

Publication bias assessment is summarized in Table S17. With the exception of special clinical populations, the meta-analyses in all population groups displayed asymmetrical Doi plots and LFK index values exceeding ±1, indicating the presence of publication bias.

### Summary estimates and pooled mean prevalence

Table 1 depicts the pooled mean prevalence estimates of syphilis stratified by population group and infection type. The pooled prevalence of probable current syphilis infection was lowest among blood donors and pregnant women at 0.004% (95% CI: 0.001%–0.025%) and 0.19% (0.06%–0.37%), respectively. It was followed by other general population groups and special clinical populations at 1.67% (95% CI: 0.55%–3.29%) and 1.765% (95% CI: 0.000%–6.154%), respectively, then populations at intermediate risk at 2.76% (95% CI: 1.51%–4.35%) and STI clinic attendees at 4.18% (95% CI: 2.08%–6.89%).

**Table 1:** Results of meta-analyses on studies reporting syphilis prevalence in the Middle East and North Africa stratified by population group and infection type.

	Prevalence Measures	Samples		Reported Prevalence	Pooled Mean Prevalence	Heterogeneity
	N	Tested	Positive	Median (%)	Estimate (95% CI)	I^2^
**Blood Donors**						
Probable current syphilis infection	21	5,080,633	510	0.01	0.004 (0.001–0.025)	99.2%
Current infection, unspecified	57	4,299,544	21,873	0.45	0.24 (0.16–0.37)	99.5%
Lifetime syphilis infection	165	6,244,958	44,231	0.48	0.36 (0.27–0.47)	99.7%
Unclear infection time	38	10,886,417	25,924	0.15	0.15 (0.08–0.27)	99.8%
**Pregnant Women**						
Probable current syphilis infection	55	5,072,647	4353	0.03	0.19 (0.06–0.37)	99.6%
Current infection, unspecified	30	1,199,523	33,623	0.48	2.07 (0.70–4.05)	100.0%
Lifetime syphilis infection	73	1,323,650	13,112	1.33	2.08 (1.33–2.98)	99.7%
Unclear infection time	32	737,296	9087	0.66	1.07 (0.45–1.94)	99.9%
**Other General Populations**						
Probable current syphilis infection	19	120,544	1627	0.84	1.67 (0.55–3.29)	99.6%
Current infection, unspecified	59	215,2422	29,919	3.30	3.00 (2.35–3.72)	98.8%
Lifetime syphilis infection	8	10,004	102	0.68	2.56 (0.26–6.77)	95.7%
Unclear infection time	3	2178	55	2.80	4.09 (0.05–13.24)	96.1%
**Populations at Intermediate Risk**						
Probable current syphilis infection	41	28,115	857	1.05	2.76 (1.51–4.35)	97.8%
Current infection, unspecified	9	3318	30	0.10	0.394 (0.000–1.470)	87.7%
Lifetime syphilis infection	54	39,214	687	1.72	2.89 (1.77–4.25)	95.9%
Unclear infection time	6	1789	36	3.31	2.51 (0.77–4.97)	63.7%
**Populations at High Risk**						
Probable current syphilis infection						
Overall	55	16,655	2205	11.46	14.83 (10.75–19.42)	97.8%
MSM & transgender people	14	2995	648	21.53	22.52 (12.73–34.06)	97.7%
FSW	41	13,660	1558	10.00	12.58 (8.45–17.35)	97.6%
Current infection, unspecified						
Overall	10	5104	289	4.44	4.50 (2.56–6.91)	91.7%
MSM & transgender people	1	1429	43	3.01	–	–
FSW	9	3675	246	4.92	4.69 (2.52–7.45)	90.3%
Lifetime syphilis infection						
Overall^a^	64	22,923	1778	3.61	6.75 (4.02–10.08)	98.2%
MSM & transgender people	21	6964	1130	4.20	7.95 (3.26–14.42)	98.9%
FSW	40	15,433	646	3.88	6.93 (3.44–11.42)	96.1%
Unclear infection time						
Overall	65	74,834	3054	4.99	7.25 (4.95–9.95)	98.6%
MSM & transgender people	29	42,195	1493	3.30	8.29 (4.33–13.35)	98.9%
FSW	36	32,639	1561	6.54	6.43 (3.91–9.50)	98.3%
**STI clinic attendees**						
Probable current syphilis infection	22	47,802	4137	4.81	4.18 (2.08–6.89)	98.3%
Current infection, unspecified	8	3749	266	6.72	6.03 (3.53–9.09)	82.9%
Lifetime syphilis infection	13	516,505	18,547	4.68	5.22 (3.11–7.82)	99.9%
Unclear infection time	9	3264	173	16.13	11.75 (3.02–24.82)	98.3%
**Special Clinical Populations**						
Probable current syphilis infection	4	937	29	1.80	1.765 (0.000–6.154)	90.0%
Current infection, unspecified	1	96	4	4.17	–	–
Lifetime syphilis infection	6	2234	63	0.98	2.333 (0.000–8.398)	94.6%
Unclear infection time	2	3066	38	2.25	1.90 (0.04–6.10)	–
**Mixed Populations**						
Probable current syphilis infection	5	13,141	124	5.00	2.71 (0.39–6.70)	97.1%
Current infection, unspecified	1	31,174	5127	16.45	–	–
Lifetime syphilis infection	0	–	–	–	–	–
Unclear infection time	0	–	–	–	–	–

CI: Confidence interval, FSW: Female sex workers, MSM: Men who have sex with men.

a Includes three studies with a mix of FSW and MSM/transgender people.

Among populations at intermediate risk, the pooled prevalence probable current syphilis infection was highest among incarcerated people at 12.34% (95% CI: 6.51%–19.63%) followed by people who inject drugs (PWID) and people living with HIV/AIDS (PLHIV) at 7–8% (Table S18). The pooled prevalence was highest among populations at high risk at 14.83% (95% CI: 10.75%–19.42%). Here, the pooled prevalence was higher in MSM and transgender people (22.52%, 95% CI: 12.73%–34.06%) compared with female sex workers (FSW) (12.58%, 95% CI: 8.45%–17.35%). The pooled prevalence among pregnant women and other general population groups combined, i.e., among the general population, was 0.48% (95% CI: 0.22%–0.82%).

The pooled prevalence of lifetime syphilis infection was lowest among blood donors at 0.36% (95% CI: 0.27%–0.47%). It was followed by pregnant women at 2.08% (95% CI: 1.33%–2.98%), special clinical populations at 2.333% (95% CI: 0.000%–8.398%), other general population groups at 2.56% (95% CI: 0.26%–6.77%), and populations at intermediate risk at 2.89% (95% CI: 1.77%–4.25%). It was highest among STI clinic attendees and populations at high risk at 5.22% (95% CI: 3.11%–7.82%) and 6.75% (95% CI: 4.02%–10.08%), respectively (Table 1). The pooled prevalence among pregnant women and other general population groups combined, i.e., among the general population, was 2.12% (95% CI: 1.39%–2.99%).

Forest plots of key meta-analyses can be found in Figure S19. There was strong evidence for heterogeneity in syphilis prevalence estimates, as shown by I^2^ being greater than 90% in most meta-analyses. This indicates true differences in prevalence estimates across studies rather than sampling variation (Table 1).

### Associations with prevalence and sources of between-study heterogeneity

Tables 2 and 3 summarize the results of the meta-regression analyses for all population groups and for only blood donors, pregnant women, and other general populations, respectively. Each of the two multivariable models explained 62% of the variability in syphilis prevalence.

**Table 2:** Results of meta-regression analysis to identify associations and sources of between-study heterogeneity in syphilis prevalence in the Middle East and North Africa, including all population groups (n = 935).

	Prevalence measures	Samples	Univariable analysis		Variance explained	Multivariable analysis^a^	
	N	Tested	RR (95% CI)	LR test p-value	R^2^	ARR (95% CI)	LR test p-value
**Population characteristics**							
**Population Type**							
Blood donors	281	26,511,552	Ref	<0.001	36.25%	Ref	<0.001
Pregnant women	190	8,333,116	1.95 (1.39–2.74)			1.56 (0.98–2.48)	
Other general populations	89	2,285,148	8.21 (5.39–12.52)			2.51 (1.67–3.77)	
Populations at intermediate risk	110	72,436	7.47 (4.97–11.22)			5.30 (3.39–8.27)	
Populations at high risk	194	119,516	22.82 (16.49–31.56)			15.75 (10.10–24.56)	
STI clinic attendees	52	571,320	18.14 (10.64–30.94)			8.07 (4.68–13.94)	
Special clinical populations	13	6333	9.23 (2.95–28.85)			5.60 (2.25–13.94)	
Mixed populations	6	44,315	10.28 (2.60–40.69)			6.29 (2.05–19.34)	
**Subregion/country**^b^							
Fertile Crescent	78	3,586,219	Ref	<0.001	28.25%	Ref	<0.001
Eastern MENA	138	16,745,891	1.90 (1.08–3.33)			0.63 (0.40–0.99)	
Pakistan	227	10,242,587	10.69 (6.46–17.69)			5.27 (3.53–7.86)	
Gulf	137	3,766,116	1.10 (0.64–1.90)			0.88 (0.57–1.36)	
North Africa	103	2,456,936	13.12 (7.44–23.15)			3.76 (2.37–5.96)	
Horn of Africa	252	1,145,987	16.81 (10.15–27.84)			4.06 (2.62–6.28)	
**Year of data collection**							
As a linear term	935	37,943,736	0.94 (0.93–0.95)	<0.001	8.72%	0.97 (0.96–0.98)	<0.001
**Sex at birth**							
Men	229	1,705,142	Ref	<0.001	13.86%	Ref	0.007
Women	379	8,464,436	0.50 (0.35–0.7)			0.75 (0.54–1.03)	
Mix of men and women	258	26,007,723	0.13 (0.09–0.19)			0.63 (0.46–0.86)	
Missing	69	1,766,435	0.23 (0.14–0.40)			0.67 (0.43–1.05)	
**Study methodology characteristics**							
**Infection type**^c^							
Probable current syphilis infection	222	10,380,474	Ref	0.901	0.00%	Ref	<0.001
Current infection, unspecified	175	7,694,930	0.91 (0.59–1.43)			1.97 (1.41–2.74)	
Lifetime syphilis infection	383	8,159,488	1.00 (0.69–1.46)			2.29 (1.75–3.01)	
Unclear infection time	155	11,708,844	1.10 (0.69–1.75)			1.96 (1.40–2.74)	
**Syphilis ascertainment**							
Assay not explicitly described	155	11,708,844	Ref	0.553	0.00%	–	
Assay explicitly described	780	26,234,892	0.89 (0.61–1.31)			–	
**Sampling method**							
Non-probability based	543	16,402,298	Ref	<0.001	10.13%	Ref	0.016
Probability based	186	79,278	3.47 (2.43–4.96)			0.62 (0.45–0.87)	
Missing	206	21,462,160	0.44 (0.31–0.62)			0.81 (0.61–1.10)	
**Precision**							
Low precision	325	65,325	Ref	<0.001	15.60%	Ref	<0.001
High precision	610	37,878,410	0.17 (0.13–0.23)			0.41 (0.32–0.53)	

ARR: Adjusted risk ratio, CI: Confidence interval, LR: Likelihood ratio, MENA: Middle East and North Africa, RR: Risk ratio, R^2^: Coefficient of determination, STI: sexually transmitted infection.

The RR represents the exponentiated beta coefficient calculated by the meta-regression model. Given that prevalence can be interpreted as the probability of an individual in the population being infected, a prevalence ratio can be interpreted as a risk ratio. Hence, we have opted to use the term “risk ratio” rather than “prevalence ratio” for epidemiological relevance.

a R^2^ = 62.1%.

b These were ordered according to the hierarchy of prevalence of STIs as commonly observed in previous studies in MENA.^20^^,^^23^

c Included in the multivariable model despite non-significance in the univariable analysis because of epidemiological relevance. Fertile Crescent includes Egypt, Iraq, Jordan, Lebanon, Palestine, Syria; North Africa includes Algeria, Libya, Morocco, Tunisia; Gulf includes: Bahrain, Kuwait, Oman, Qatar, Saudi Arabia, and United Arab Emirates; Horn of Africa includes: Djibouti, Somalia, Sudan, recently independent South Sudan, and Yemen.

**Table 3:** Results of meta-regression analyses to identify associations and sources of between-study heterogeneity in syphilis prevalence in the Middle East and North Africa, including only blood donors, pregnant women, and other general population groups (n = 560).

	Prevalence measures	Samples	Univariable analysis		Variance explained	Multivariable analysis^a^	
	N	Tested	RR (95% CI)	LR test p-value	R^2^	ARR (95% CI)	LR test p-value
**Population characteristics**							
**General Population Sub-type**							
Blood Donors	281	26,511,552	Ref	<0.001	13.18%	Ref	<0.001
Pregnant women	190	8,333,116	1.91 (1.30–2.81)			1.09 (0.51–2.31)	
Other general populations	89	2,285,148	8.22 (5.07–13.33)			2.18 (1.37–3.46)	
**Subregion/country**^b^							
Fertile Crescent	65	3,572,481	Ref	<0.001	50.31%	Ref	<0.001
Eastern MENA	58	16,168,072	0.24 (0.13–0.44)			0.32 (0.18–0.57)	
Pakistan	169	10,219,437	8.28 (5.23–13.12)			4.97 (3.13–7.92)	
Gulf	100	3,685,834	0.69 (0.41–1.14)			0.94 (0.58–1.53)	
North Africa	40	2,397,224	2.52 (1.36–4.66)			2.72 (1.53–4.86)	
Horn of Africa	128	1,086,768	18.88 (11.59–30.75)			13.62 (7.88–23.55)	
**Year of data collection**							
As a linear term	560	37,129,816	0.94 (0.91–0.96)	<0.001	5.64%	0.97 (0.95–0.99)	0.009
**Sex at birth**							
Men	76	1,611,859	Ref	<0.001	7.88%	Ref	0.004
Women	220	8,380,276	0.34 (0.20–0.59)			0.46 (0.22–0.96)	
Mix of men and women	202	25,407,283	0.17 (0.10–0.28)			0.53 (0.36–0.79)	
Missing	62	1,730,399	0.38 (0.19–0.75)			0.56 (0.33–0.94)	
**Study methodology characteristics**							
**Infection type**							
Probable current infection	95	10,273,824	Ref	<0.001	9.42%	Ref	<0.001
Current infection, unspecified	146	7,651,489	7.00 (4.00–12.27)			2.62 (1.70–4.05)	
Lifetime syphilis infection	246	7,578,612	5.22 (3.10–8.80)			2.77 (1.82–4.23)	
Unclear infection time	73	11,625,891	2.19 (1.14–4.21)			2.10 (1.27–3.49)	
**Syphilis ascertainment**^c^							
Assay not explicitly described	73	11,625,891	Ref	0.013	1.06%	–	
Assay explicitly described	487	25,503,925	1.97 (1.15–3.36)			–	
**Sampling method**							
Non-probability based	405	15,727,994	Ref	<0.001	7.31%	Ref	0.434
Probability based	13	14,854	1.53 (0.43–5.46)			0.56 (0.23–1.36)	
Missing	142	21,386,968	0.27 (0.18–0.41)			0.97 (0.66–1.42)	
**Precision**							
Low precision	140	33,993	Ref	<0.001	19.41%	Ref	<0.001
High precision	420	37,095,823	0.11 (0.08–0.17)			0.40 (0.27–0.57)	

ARR: Adjusted risk ratio, CI: Confidence interval, LR: Likelihood ratio, MENA: Middle East and North Africa, RR: Risk ratio, R^2^: Coefficient of determination.

The RR represents the exponentiated beta coefficient calculated by the meta-regression model. Given that prevalence can be interpreted as the probability of an individual in the population being infected, a prevalence ratio can be interpreted as a risk ratio. Hence, we have opted to use the term “risk ratio” rather than “prevalence ratio” for epidemiological relevance.

a R^2^ = 61.9%.

b These were ordered according to the hierarchy of prevalence of STIs as commonly observed in previous studies in MENA.^20^^,^^23^

c Not included in the multivariable model because of identified collinearity with infection type. Fertile Crescent includes Egypt, Iraq, Jordan, Lebanon, Palestine, Syria; North Africa includes Algeria, Libya, Morocco, Tunisia; Gulf includes: Bahrain, Kuwait, Oman, Qatar, Saudi Arabia, and United Arab Emirates; Horn of Africa includes: Djibouti, Somalia, Sudan, recently independent South Sudan, and Yemen.

Syphilis prevalence exhibited strong association with population group which, alone, explained 36.3% of the variation in prevalence (Table 2). Relative to blood donors, the prevalence was highest among populations at high risk (ARR = 15.75, 95% CI: 10.10–24.56) followed by STI clinic attendees (ARR = 8.07, 95% CI: 4.68–13.94). Regional differences were observed, with Pakistan, North Africa, and the Horn of Africa each exhibiting about 4–5 times higher prevalence than the Fertile Crescent. The prevalence was lower among women compared with men (ARR = 0.75, 95% CI: 0.54–1.03) and higher for lifetime versus probable current syphilis infection (ARR = 2.29, 95% CI: 1.75–3.01).

Studies with larger sample sizes and those using probability-based sampling methods were more likely to detect lower syphilis prevalence than their counterparts (ARR = 0.41, 95% CI: 0.32–0.53 and ARR = 0.62, 95% CI: 0.45–0.87, respectively). There was evidence for decreasing prevalence over time at an average rate of 3% per year (ARR = 0.97, 95% CI: 0.96–0.98) (Table 2). This trend was consistent when the year of data collection was also modeled as a categorical variable (Table S20). There was a steady decrease in syphilis prevalence by decade, with prevalence being 67% lower after 2020 compared with before 2000 (ARR = 0.33, 95% CI: 0.20–0.57) (Table S20). Meta-regression analysis for only probable current syphilis infection showed similar time trends, with a 4% decrease in prevalence per year (ARR = 0.96, 95% CI: 0.94–0.99) (data not shown).

The meta-regression analysis conducted among only general population groups showed similar results, summarized in Table 3. Compared with blood donors, pregnant women had similar syphilis prevalence (ARR = 1.09, 95% CI: 0.51–2.31) while other general population groups had significantly higher prevalence (ARR = 2.18, 95% CI: 1.37–3.46). Substantial heterogeneity in prevalence was observed across different subregions. The Horn of Africa exhibited the highest prevalence, followed by Pakistan and North Africa. In contrast, lower prevalence was observed in Afghanistan and Iran when compared to the Fertile Crescent. The prevalence was lower among women compared with men (ARR = 0.46, 95% CI: 0.22–0.96) and higher for lifetime versus probable current syphilis infection (ARR = 2.77, 95% CI: 1.82–4.23). There was evidence for decreasing prevalence over time among general population groups at an average rate of 3% per year (ARR = 0.97, 95% CI: 0.95–0.99) (Table 3). Syphilis prevalence after 2020 was 60% lower compared with before 2000 (ARR = 0.40, 95% CI: 0.20–0.78) (Table S21). Meta-regression analysis for only probable current syphilis infection showed similar time trends, with a 4% decrease in prevalence per year (ARR = 0.96, 95% CI: 0.92–1.01) (data not shown).

In a separate meta-regression analysis specifically conducted among populations at high risk, comparable results were observed (Table S22), but the rate of prevalence decrease was higher at 8% per year (ARR = 0.92, 95% CI: 0.89–0.94). The meta-regression analysis explained 52% of the variation in syphilis prevalence. A consistent and steady trend of decreasing prevalence was observed over time when the year of data collection was modeled categorically (Table S23). There was evidence for lower prevalence among FSW compared to MSM and transgender people (ARR = 0.69, 95% CI: 0.49–0.96) (Table S23). Meta-regression analysis for only probable current syphilis infection showed similar time trends, with a 9% decrease in prevalence per year (ARR = 0.91, 95% CI: 0.86–0.97) (data not shown).

## Discussion

A comprehensive examination of syphilis infection epidemiology in MENA was undertaken. The pooled prevalence of probable current syphilis infection among the general population was 0.48%. Given the sexually conservative norms and relatively low levels of various viral STIs in MENA,^30^^,^^32^^,^^59^ this substantial level of prevalence is unexpected. While it suggests the existence of active transmission networks for this infection, it may not strictly indicate high levels of risky sexual behaviors in the population at large.^20^ This level of prevalence could be partly attributed to inadequate access to and utilization of STI screening and treatment services in the region,^19^, ^20^, ^21^ especially that a large fraction of syphilis infections are asymptomatic and hence would not present for testing and subsequent treatment. MENA faces inadequate capacity in terms of programs for STI prevention and treatment.^19^, ^20^, ^21^ Limited screening will result in a large number of undiagnosed infections which, when left untreated, will persist for an extended duration, thereby increasing the potential for transmission within the population. Similar observations in other regions have indicated that the limited availability of curable STI diagnosis and specific treatment can lead to unusually high prevalence rates.^60^, ^61^, ^62^ It is worth noting several countries in MENA have faced challenges in procuring benzathine penicillin over the past few years. However, they have generally succeeded in securing sufficient supplies to meet the demands, often by maintaining reserves.

The estimated syphilis prevalence of 0.48% is comparable, albeit slightly lower than the syphilis prevalence in the global population in 2020, which was estimated at 0.6%.^7^ It is also similar to the WHO model estimate of 0.62% in 2020 for the Eastern Mediterranean Region,^7^ and a meta-analysis by Smolak et al., which estimated a prevalence of 0.63% for this region for the period 1990–2016.^12^ Differences in estimates could be attributed to variations in infection definition and diagnostics used, with current infection defined using both stringent and less stringent criteria, such as in relation to the rapid plasma reagin test titers,^63^ differences in the time periods considered, data sources, and estimation methodologies. The WHO estimate relied mainly on the SPECTRUM-STI statistical trend-fitting model database,^64^ supplemented with antenatal surveys and routine programmatic screening data reported by countries through the Global AIDS Monitoring (GAM) system. Smolak et al. primarily extracted antenatal care prevalence data from repositories such as the Global AIDS Response Progress Reporting (GARP) system,^65^ (now GAM), and the Global Burden of Disease Study database.^66^ In contrast, this study employed a region-specific systematic review of all published data and incorporated all available routine testing data for the various population groups.

Syphilis prevalence in MENA demonstrated a hierarchical pattern, with higher levels observed among higher-risk populations, including MSM and FSW, similar to patterns observed in other STIs.^20^^,^^30^^,^^32^^,^^46^^,^^67^ These findings underscore the imperative to address this infection among key populations where syphilis transmission remains sustained at significant levels. This is further substantiated by behavioral data from MENA, indicating the frequent occurrence of transactional sex among attendees of STI clinics,^68^^,^^69^ as well as considerable levels of sexual risk behavior within key populations.^32^^,^^34^, ^35^, ^36^ These populations are also experiencing growing HIV epidemics,^32^^,^^34^, ^35^, ^36^^,^^70^ emphasizing the necessity for tailored treatment and prevention interventions to meet the specific needs of these groups.

There was substantial regional heterogeneity in syphilis prevalence within MENA, similar to the patterns observed for other STIs in MENA, with the Horn of Africa typically experiencing the highest levels.^20^^,^^28^^,^^30^^,^^32^^,^^34^ Subregion alone explained 50% of the variation in syphilis prevalence among general population groups. Syphilis prevalence was higher among men compared to women, potentially due to higher levels of engagement in sexual risk behavior among men and potential underreporting of engagement in MSM networks.^35^^,^^71^ Conversely, syphilis prevalence was lowest among blood donors, as expected due to donor selection protocols and blood safety regulations.^72^ Despite the variation in blood donor selection protocols and criteria in the region, the vast majority of blood donations are unpaid (both voluntary and family/replacement donations), which are considered the safest and typically have the lowest prevalence of bloodborne pathogens.^29^^,^^72^ The prevalence of syphilis among pregnant women and other general populations combined (i.e., among the general population) was about two-fold higher than that among blood donors (ARR = 1.89, 95% CI: 1.22–2.92, data not shown), highlighting the selection of lower-risk individuals for blood donation. This difference in prevalence between the general population and blood donors at 1.9-fold was comparable to the 1.7-fold observed for hepatitis C virus prevalence in the region.^29^

The analyses indicated a trend of decreasing syphilis prevalence in both general populations and populations at high risk. However, populations at high risk exhibited a higher rate of decrease, with an 8% decline compared to a 3% decline in general populations. This finding aligns with the global trend observed over the past three decades, as documented in previous studies.^11^^,^^12^ A decline of 7% per year in prevalence was also observed among FSW in MENA.^23^ In contrast, recent increasing trends have been predominantly observed among MSM and transgender people in several countries with adequate syphilis surveillance systems such as the USA and China.^11^ These increases in other regions may be attributed to increased sexual risk behavior following the introduction of ART and PrEP, interventions that have limited coverage in MENA's relatively nascent HIV epidemics.^32^ Alternatively, any small and recent increase in syphilis prevalence among populations at high risk in MENA may not have been captured by available data, given the relatively small number of studies conducted in the last few years (Figure S15).

The declining syphilis prevalence in MENA can be attributed to several factors. One contributing factor may be the practice of safer sex following the HIV epidemic,^73^ coupled with increased condom use for the prevention of both HIV transmission and unwanted pregnancies.^34^ Another potential factor is a shorter duration of active syphilis infection in the population,^15^^,^^74^ possibly resulting from progressive improvements in syphilis screening and treatment coverage, especially in antenatal care (ANC) settings, advancements in syphilis diagnostics and treatment methods, or the widespread and increasing use of antibiotics.^75^ The high prevalence of self-medication with antibiotics, over-prescription of antibiotics in health-care facilities and hospitalized settings, and the overall poor regulatory enforcement in the region lead to extensive use of antibiotics which, even if intended for non-STI infections, may inadvertently cure concurrent, often asymptomatic syphilis infections.^12^^,^^76^^,^^77^

While it is possible that the COVID-19 pandemic may have contributed to the observed decline in syphilis prevalence in the most recent years due to changes in sexual activity, sexual risk behavior, and access to testing services,^78^^,^^79^ only 5% of the studies in this review were conducted after 2020. A sensitivity analysis excluding these studies confirmed the steady declining syphilis prevalence over time (data not shown).

This study has limitations. Although data were collected from all 24 MENA countries, there was variability in data availability across different countries and for various population groups (Table S13). The number and sample size of prevalence measures among certain population groups–namely MSM and transgender people, PWID, PLHIV, and incarcerated people–were relatively small, potentially compromising the representativeness of the estimates for these groups. Moreover, all of these key and vulnerable populations are highly stigmatized in the region, even in healthcare settings.^80^ Out of the 24 MENA countries, nine have laws prohibiting possession of drugs for personal use, 21 have punitive laws against sex work, and 19 have punitive laws against same-sex sex including death penalty in 7 of the countries.^81^ Populations with the highest risk of exposure to STIs, such as male sex workers, tend to be more visible and may be overrepresented in research studies because of ease of recruitment.^35^^,^^82^ This could lead to an overestimation of STI prevalence in these studies. However, most studies among key populations in the region use state-of-the-art, probability-based sampling methodologies, such as respondent-driven sampling,^47^ which are specifically designed for hard-to-reach populations and may help reduce this bias. Our review did not identify any studies that reported on the prevalence of syphilis among sexual partners of individuals with syphilis, unlike other regions where such STI studies are common.^83^

Furthermore, limited recent data were available for specific population groups, namely STI clinic attendees and special clinical populations (Table S14 and Figure S15), suggesting that the pooled syphilis prevalence estimates for these groups may not accurately reflect current prevalence. Nevertheless, most data for the primary population groups, such as general populations and populations at high risk, were recent and collected after 2010 (Tables S13 and S14 and Figure S15). Heterogeneity in syphilis prevalence was noted among the included studies; however, the meta-regression analyses explained over 60% of this heterogeneity by considering various epidemiological factors and study methods.

While our categorization of syphilis is strictly based on serological test results rather than infection type, we assigned each category a general interpretation for infection, for epidemiological relevance. However, accurately inferring a history of syphilis diagnosis from serological test results alone is challenging and comes with several caveats (Table S2). For example, in individuals with a history of adequately treated syphilis infection, non-treponemal tests may remain reactive for many years.^41^ Therefore, without using antibody titers cutoffs, it is possible that some individuals who tested positive on both treponemal and non-treponemal tests, and were thus classified as having a probable current syphilis infection, may actually have a past, adequately treated infection rather than an current infection. This potential misclassification may result in an overestimation of the prevalence of probable current syphilis infection.

In a recent global systematic review of syphilis prevalence among MSM, only 31 out of 345 data points were based on quantitative syphilis diagnostic methods with titers cutoffs.^24^ While these showed slightly lower prevalence estimates than those from qualitative methods, the sensitivity analysis conducted by the authors showed that this bias did not appreciably influence the overall estimates of current syphilis infection.^24^ It is, therefore, possible that the impact of this misclassification bias on our prevalence estimates of probable current syphilis infection may not be appreciable. In the absence of a practical gold standard for syphilis diagnosis, a positive result on both a treponemal and non-treponemal test, even without antibody titers cutoff, remains the standard diagnostic strategy for probable current syphilis infection.^12^^,^^22^^,^^42^^,^^44^

Misclassification bias can also affect the other infection type categories. Individuals classified as having a possible current, unspecified infection based on only a reactive non-treponemal test may include biological false positives caused by febrile illnesses, connective tissue diseases, pregnancy, malaria, tuberculosis, and others, given there was no confirmation of syphilis infection with a treponemal test in this category.^41^ This can lead to an overestimation of the prevalence of current infection. Similarly, individuals classified as having a lifetime syphilis infection based on only a reactive treponemal test may include both current and past infections, and would need further serological testing using quantitative non-treponemal tests. Cross-reactivity with antibodies against other non-venereal treponematoses, particularly in Horn of Africa countries, may also exist, as current serological diagnostics may not distinguish between syphilis and other endemic treponematoses such as yaws, bejel, and pinta^84^ (Table S2). As such, while our categorization of syphilis based on test results is unambiguous, it is the interpretation of these test results in terms of infection type and history that should be considered within the context of these limitations.

Another limitation lies in the diverse diagnostic tests used in the identified studies, which may exhibit variations in sensitivity and specificity. Although our analyses were stratified based on broad testing modalities (non-treponemal, treponemal, or a combination of both), we were unable to account for potential individual-test diagnostic biases within each category. Some studies did not explicitly specify the particular biological assay employed for infection ascertainment, and therefore a characterization of the syphilis diagnosis cannot be made. These studies were included for completeness but were analyzed separately. We noted an increase in the use of point-of-care tests in the last decade, which are increasingly recognized as reliable, accessible, and convenient options, particularly in resource-limited settings and in underserved and key populations.^41^

A strong small-study effect was evident and confirmed by the analysis that demonstrated the presence of publication bias. Studies featuring a sample size of at least 200 for populations at high risk and STI clinic attendees, and at least 500 for all other population groups, reporting 60% lower prevalence levels (Table 2). This observation remained consistent in a sensitivity analysis investigating the small study effect separately for programmatic data (ANC and blood donor data) versus all other epidemiological studies among the other population groups (data not shown). However, the majority of studies included in the analyses had large sample sizes (Table S16), enhancing the precision of the estimates. The sampling methods of available studies varied, with many relying on convenience sampling methods that may introduce selection bias. Convenience samples, particularly in facility-based studies, might over-represent higher-risk individuals, aligning with the higher prevalence observed in studies utilizing non-probability-based sampling methodologies (Table 2).

Studies employing less rigorous methods overall tended to report higher syphilis prevalence, whereas higher-quality studies tended to report lower prevalence. Notably, some studies reported unexpectedly high prevalence values even among populations presumed to have a low risk of infection, indicating potential unreported biases such as in sample recruitment. The median of prevalence measures frequently diverged considerably from the pooled mean due to skewed distributions in the available prevalence measures (Table 1). It is important to acknowledge these limitations when interpreting the findings, recognizing the potential biases inherent in the available studies.

This study has several strengths. The review successfully identified a substantial and recent volume of data from both published and programmatic sources. A large fraction of studies in the region remain unpublished in the scientific literature, including some of the best quality studies. This review accessed the UNAIDS IBBSS database and the WHO Global Health Observatory database, and, as such, presents a large volume of data that will appear in the scientific literature for the first time. Notably, the review identified a significant amount of data on populations at high risk, representing nearly 30% of prevalence measures (Table S13). These populations are often underrepresented in investigations of other STIs in MENA.^20^^,^^28^^,^^30^^,^^32^^,^^85^ This comprehensive data collection effort provides a detailed understanding of syphilis epidemiology in MENA for the first time, filling critical knowledge gaps on STIs in this region. Historically, STIs in MENA, including syphilis, have been neglected in terms of research and public health response.^19^, ^20^, ^21^ The study's diverse data and analytical approaches shed light on syphilis epidemiology across different populations and settings. Consequently, the study meaningfully contributes to filling knowledge gaps on syphilis in the global context, facilitating evidence-based decision-making and the implementation of effective strategies to address the burden of syphilis and improve sexual health outcomes in the region.

In conclusion, the prevalence of probable current syphilis infection in MENA is approximately 0.5%, comparable to syphilis prevalence in the global population. However, there is evidence of a decreasing trend over the last few decades, with a 3% annual decline among general populations and a more substantial 8% decline among populations at high risk. Despite this progress, the rate of decline remains well below the 17% annual reduction required to achieve the ambitious target of a 90% reduction in syphilis incidence by 2030.^18^ Syphilis prevalence exhibited significant variability across MENA subregions and has elevated levels among populations at high risk, including MSM, transgender people, and FSW. These findings underscore a significant yet often overlooked disease burden with potential implications for reproductive health and social well-being.

Substantial challenges persist in controlling syphilis and eliminating congenital syphilis, with the current response falling short of the targets set by the WHO's Global Health Sector Strategy on STIs. Control efforts are further complicated by obstacles such as STI stigma, socio-cultural sensitivities, and ongoing conflict and humanitarian crises, which hinder the establishment of an inclusive public health agenda and a supportive environment for sexual health. To address these challenges, actions are needed, including increasing antenatal screening coverage across the region, integrating syphilis testing into testing for key populations, combining STI services with HIV and other health program areas, and promoting partner services which remain limited in the region. Critical steps involve the development of targeted, culturally sensitive, and gender-specific programs to expand prevention and treatment services. Ideally, STI programs and strategic plans should be integrated with HIV and viral hepatitis programs for optimal effectiveness. Maximizing the availability and utilization of affordable, practical, and highly sensitive rapid syphilis tests is critical. Finally, expanding STI surveillance and research efforts is essential to monitor trends, inform public health responses, and allocate resources effectively.

## Contributors

MEJ conducted systematic searches, screening of records, and data extraction, conducted meta-analytics, and contributed to writing the first draft of the manuscript. BA, AAT, MH, and SA conducted systematic searches, screening of records, and data extraction. IF contributed to double screening of reports, double extraction of data, and the conduct of the meta-analyses. FD and ES contributed to double screening of reports and double extraction of data. MSJ, ASA, JH, and JR facilitated access to unpublished sources of data. LJA co-conceptualized the study, co-designed the methodology, and co-wrote the first draft of the manuscript. GRM co-conceptualized the study, co-designed the methodology, supervised the systematic review and meta-analytics, and co-wrote the first draft of the manuscript. MEJ, JR, and GRM accessed and verified the data. All authors contributed to discussion and interpretation of the results and to writing of the manuscript. All authors have read and approved the final manuscript and were responsible for the decision to submit the manuscript.

## Data sharing statement

The dataset supporting the conclusions of this article is included within the article and its Supplementary Material.

## Declaration of interests

The authors declare no competing interests.
