# Supplementary material for: Syphilis infection prevalence in the Middle East and North Africa: a systematic review and meta-analysis
Source: eClinicalMedicine. 2024 Jul 29;75:102746. doi: 10.1016/j.eclinm.2024.102746 (PMC11701444; doi:10.1016/j.eclinm.2024.102746)
Supplement: Supplementary Tables and Figures [file mmc1.docx]

**SUPPLEMENTARY MATERIAL**

**Table of Content**

[**Table S1.** Search criteria for the systematic search of syphilis prevalence in the Middle East and North Africa 3](#_Toc172403510)

[**Table S2.** Interpretation and limitations in the interpretation of syphilis serological test results in the diagnosis of syphilis infection 4](#_Toc172403511)

[**Table S3.** Syphilis studies among blood donors in the Middle East and North Africa, stratified by infection type 6](#_Toc172403512)

[**Table S4.** Syphilis studies among pregnant women in the Middle East and North Africa, stratified by infection type 10](#_Toc172403513)

[**Table S5.** Syphilis studies among other general population groups in the Middle East and North Africa, stratified by infection type 12](#_Toc172403514)

[**Table S6.** Syphilis studies among populations at intermediate risk in the Middle East and North Africa, stratified by infection type 15](#_Toc172403515)

[**Table S7.** Syphilis studies among populations at high risk in the Middle East and North Africa, stratified by infection type 20](#_Toc172403516)

[**Table S8.** Syphilis studies among STI clinic attendees in the Middle East and North Africa, stratified by infection type 26](#_Toc172403517)

[**Table S9.** Syphilis studies among special clinical populations in the Middle East and North Africa, stratified by infection type 29](#_Toc172403518)

[**Table S10.** Syphilis studies among mixed populations in the Middle East and North Africa, stratified by infection type 31](#_Toc172403519)

[**Table S11.** Syphilis studies in the Middle East and North Africa as extracted from the WHO Global Health Observatory data repository 32](#_Toc172403520)

[**Figure S12.** Trend of syphilis prevalence over time, all prevalence measures included in the meta-analytics. 35](#_Toc172403521)

[**Table S13.** Distribution of syphilis prevalence studies by key characteristics in the Middle East and North Africa. 36](#_Toc172403522)

[**Table S14.** Time span of syphilis prevalence studies by population type in the Middle East and North Africa. 37](#_Toc172403523)

[**Figure S15.** Number of studies reporting probable current syphilis infection prevalence measures by year and type of population in the Middle East and North Africa. 38](#_Toc172403524)

[**Table S16.** Summary of precision and risk of bias of syphilis prevalence measures in the Middle East and North Africa. 39](#_Toc172403525)

[**Table S17.** Assessment of publication bias using Doi plots and the LFK index. 40](#_Toc172403526)

[**Table S18.** Results of meta-analyses on studies reporting probable syphilis infection prevalence among people who inject drugs, people living with HIV/AIDS, and incarcerated people in the Middle East and North Africa. 41](#_Toc172403527)

[**Figure S19.** Forest plots presenting outcomes of the pooled mean probable current syphilis prevalence among different populations in the Middle East and North Africa. 42](#_Toc172403528)

[A. Blood donors 42](#_Toc172403529)

[B. Pregnant women 43](#_Toc172403530)

[C. Other general population groups 44](#_Toc172403531)

[D. Populations at intermediate risk 45](#_Toc172403532)

[E. Populations at high risk (overall) 46](#_Toc172403533)

[F. Populations at high risk (men who have sex with men & transgender people) 47](#_Toc172403534)

[G. Populations at high risk (female sex workers) 48](#_Toc172403535)

[H. STI clinic attendees 49](#_Toc172403536)

[I. Special clinical populations 50](#_Toc172403537)

[J. Mixed populations 51](#_Toc172403538)

[**Table S20*.*** Results of meta-regression analysis to identify associations and sources of between-study heterogeneity in syphilis prevalence in the Middle East and North Africa, including all population groups and using year as a categorical variable. 52](#_Toc172403539)

[**Table S21.** Results of meta-regression analysis to identify associations and sources of between-study heterogeneity in syphilis prevalence in the Middle East and North Africa, including only blood donors, pregnant women, and other general population groups and using year as a categorical variable. 54](#_Toc172403540)

[**Table S22.** Results of meta-regression analysis to identify associations and sources of between-study heterogeneity in syphilis prevalence in the Middle East and North Africa, including only populations at high risk. 56](#_Toc172403541)

[**Table S23.** Results of meta-regression analysis to identify associations and sources of between-study heterogeneity in syphilis prevalence in the Middle East and North Africa, including only populations at high risk and using year as a categorical variable. 58](#_Toc172403542)

[**References** 60](#_Toc172403543)

# Table S1. Search criteria for the systematic search of syphilis prevalence in the Middle East and North Africa.

| **PUBMED** |
| --- |
| (“Arabs”[Mesh] OR “Arab world”[Mesh] OR “Islam”[Mesh] OR “Africa, Northern”[Mesh] OR “Middle East”[Mesh] OR “Djibouti”[Mesh] OR “Pakistan”[Mesh] OR “Somalia”[Mesh] OR “South Sudan”[Mesh] OR “Sudan”[Mesh] OR Arabs[Text] OR Arab[Text] OR Islam[Text] OR North Africa[Text] OR Northern Africa[Text] OR Middle East[Text] OR Eastern Mediterranean[Text] OR Afghanistan[Text] OR Algeria[Text] OR Bahrain[Text] OR Djibouti[Text] OR Egypt[Text] OR Iran[Text] OR Iraq[Text] OR Jordan[Text] OR Kuwait[Text] OR Lebanon[Text] OR Libya[Text] OR Morocco[Text] OR Oman[Text] OR Pakistan[Text] OR Qatar[Text] OR Saudi Arabia[Text] OR Somalia[Text] OR Sudan[Text] OR Syria[Text] OR Syrian Arab Republic[Text] OR Tunisia[Text] OR United Arab Emirates[Text] OR Abu Dhabi[Text] OR Ajman[Text] OR Al Ain[Text] OR Dubai[Text] OR Ras Al Khaimah[Text] OR Sharjah[Text] OR West Bank[Text] OR Gaza[Text] OR Palestine[Text] OR Yemen[Text]) AND (“Syphilis”[Mesh] OR “Treponema pallidum”[Mesh] OR Syphilis[Text] OR Treponema pallidum[Text] OR Great Pox[Text]) |
| **EMBASE** |
| (exp Arab/ OR exp Arab world/ OR exp Islam/ OR exp north africa/ OR exp middle east/ OR exp Afghanistan/ OR exp Djibouti/ OR exp Pakistan/ OR exp Somalia/ OR exp South Sudan/ OR exp Sudan/ OR (arab OR arabs OR Islam OR north africa OR northern Africa OR eastern mediterranean OR middle east OR afghanistan OR algeria OR bahrain OR djibouti OR egypt OR iran OR iraq OR jordan OR kuwait OR lebanon OR libya OR morocco OR oman OR pakistan OR qatar OR saudi arabia OR somalia OR sudan OR Syria OR syrian arab republic OR tunisia OR united arab emirates OR abu dhabi OR ajman OR al Ain OR dubai OR ras al khaimah OR sharjah OR west bank OR gaza OR palestine OR yemen).mp.) AND (exp syphilis/ OR exp treponema pallidum/ OR (great pox OR syphilis OR treponema pallidum).mp.) |
| **International Conferences** |
| Free text search using only each individual country name of each country in the Middle East and North Africa (The search did not include the term 'syphilis') |

# Table S2. Interpretation and limitations in the interpretation of syphilis serological test results in the diagnosis of syphilis infection.^1-3^

| **Category** | **Serological test results** | **Interpretation** | **Limitations in interpretation** |
| --- | --- | --- | --- |
| *Probable current (active) syphilis infection* | Positive both treponemal and non-treponemal tests (irrespective of antibody titers^*^) | - There is a probable active syphilis infection. - Non-treponemal antibody titers are needed to confirm whether the infection is currently active. | - May include past, adequately treated infections. - May miss very recent infections. - May include non-treponemal biological false positives caused by febrile illnesses, connective tissue diseases, pregnancy, malaria, tuberculosis, and others. - May include treponemal biological false positives caused by reactivity to endemic rather than venereal treponematoses such as yaws, bejel and pinta. |
| *Possible current infection, unspecified* | Positive non-treponemal test only, irrespective of treponemal test result if done | - Reactivity is suggestive of an active infection which, however, could be a false positive. - Non-treponemal antibody titers are needed to indicate whether the infection is currently active. - Further serological testing using treponemal tests is needed to confirm syphilis infection. | - May miss very recent infections. - May include non-treponemal biological false positives caused by febrile illnesses, connective tissue diseases, pregnancy, malaria, tuberculosis, and others. |
| *Lifetime syphilis infection* | Positive treponemal test only, irrespective of non-treponemal test result if done | - Evidence of ever infection with syphilis. - Includes both current and past infections. - Further serological testing using non-treponemal tests is needed to determine if the infection is active. | - May miss recent infections. - May include treponemal biological false positives caused by reactivity to endemic rather than venereal treponematoses such as yaws, bejel and pinta. |

^*^ Not used in the majority of studies identified

# Table S3. Syphilis studies among blood donors in the Middle East and North Africa, stratified by infection type.^*^

| **Country** | **Citation** | **Collection year** | **City** | **Sampling** | **Sex** | **Assay^✝^** | **Tested**  **(N)** | **Prevalence**  **(%)** |
| --- | --- | --- | --- | --- | --- | --- | --- | --- |
| Probable Current Syphilis Infection | |  |  |  |  |  |  |  |
| Iran | Keshvari, 2015^4^ | 2013 | Tehran | Convenience | Mix | RPR & FTA-ABS | 269,602 | 0.00 |
| Iran | Keshvari, 2015^4^ | 2012 | Tehran | Convenience | Mix | RPR & FTA-ABS | 284,429 | 0.00 |
| Iran | Keshvari, 2015^4^ | 2011 | Tehran | Convenience | Mix | RPR & FTA-ABS | 290,493 | 0.00 |
| Iran | Keshvari, 2015^4^ | 2010 | Tehran | Convenience | Mix | RPR & FTA-ABS | 288,963 | 0.00 |
| Iran | Keshvari, 2015^4^ | 2009 | Tehran | Convenience | Mix | RPR & FTA-ABS | 271,605 | 0.00 |
| Iran | Keshvari, 2015^4^ | 2008 | Tehran | Convenience | Mix | RPR & FTA-ABS | 275,511 | 0.00 |
| Iran | Khedmat, 2007^5^ | 2005-2006 | Tehran | Convenience | Mix | VDRL & FTA-ABS | 318,029 | 0.01 |
| Iran | Khedmat, 2009^6^ | 2003-2005 | Tehran | Convenience | Mix | VDRL & FTA-ABS | 1,004,889 | 0.00 |
| Iran | Khoda, 2011^7^ | 2006 | Tehran | Convenience | Mix | RPR & FTA-ABS | 14,320 | 0.00 |
| Iran | Mohammadali, 2014^8^ | 2005-2011 | Tehran | Convenience | Mix | RPR & FTA-ABS | 2,026,628 | 0.01 |
| Iraq | A. L-Erjan, 2018^9^ | 2016-2017 | Baghdad | Convenience | Mix | VDRL & (TPHA or FTA-ABS or EIA) | 28,287 | 0.67 |
| KSA | Alaidarous, 2018^10^ | 2015-2017 | Majmaah | Convenience | Mix | RPR & TPHA | 3,028 | 0.53 |
| KSA | Alshehri, 2021^11^ | NR | Najran | Convenience | Mix | POCT & RPR & ELISA | 955 | 0.20 |
| KSA | Hossain, 1986^12^ | NR | NR | Convenience | Mix | VDRL & TPHA & FTA-ABS | 1,263 | 1.74 |
| Sudan | Omer, 1982^13^ | 1978 | Khartoum | Convenience | Male | VDRL & FTA-ABS | 2,201 | 0.00 |
| Possible Current Infection, Unspecified | |  |  |  |  |  |  |  |
| Egypt | El-Gilany, 2006^14^ | 2002-2003 | Mansoura | Convenience | Mix | Wassermann Reaction Assay | 2,157 | 0.05 |
| Egypt | Nada, 2013^15^ | 1996-2011 | Ismailia | Convenience | Mix | VDRL | 148,381 | 0.00 |
| Iran | Emadi, 2021^16^ | 2009-2019 | Western Azerbaijan Province | Convenience | Mix | RPR | 682,171 | 0.04 |
| Iraq | Al-Alwani, 2018^17^ | 2017-2018 | Al-Anbar | Convenience | Mix | VDRL | 10,697 | 1.09 |
| KSA | Alabdulmonem, 2020^18^ | 2017-2018 | Buraidah | Convenience | Mix | VDRL | 4,590 | 0.04 |
| KSA | Alcantara, 2018^19^ | 2013-2015 | Hail | Convenience | Mix | VDRL | 9,000 | 0.20 |
| KSA | Bashawri, 2002^20^ | 1996-2000 | Khobar | Convenience | NR | RPR | 17,422 | 1.03 |
| KSA | Sarah, 2016^21^ | 2014-2015 | NR | Convenience | Mix | RPR | 361 | 1.66 |
| Pakistan | Attaullah, 2012^22^ | 2008-2011 | Peshawar | Convenience | Mix | VDRL | 127,828 | 0.43 |
| Pakistan | Karim, 2018^23^ | 2005-2016 | Karachi | Convenience | Mix | VDRL or RPR | 289,066 | 0.40 |
| Pakistan | Khan, 2012^24^ | 2009-2012 | Peshawar | Convenience | Mix | VDRL | 6,513 | 0.86 |
| Pakistan | Manzoor, 2009^25^ | 2008 | Lahore | Convenience | Mix | VDRL | 6,659 | 0.53 |
| Pakistan | Nepal, 2016^26^ | 2013-2014 | Karachi | Convenience | Mix | VDRL | 6,996 | 2.43 |
| Pakistan | Qadeer, 2013^27^ | 2007-2012 | Lahore | Convenience | NR | RPR | 5,000 | 0.42 |
| Pakistan | Sultan Sadia, 2016^28^ | 2005-2014 | Karachi | Convenience | Mix | RPR | 148,268 | 0.92 |
| Pakistan | Sultan, 2007^29^ | 1996-2005 | Lahore | Convenience | NR | RPR | 41,498 | 0.43 |
| Pakistan | Waheed, 2012^30^ | 2010-2011 | Islamabad | Convenience | NR | RPR | 10,145 | 1.20 |
| Pakistan | Zehra, 2013^31^ | NR | NR | Convenience | Male | RPR | 6,585 | 1.15 |
| Pakistan | Zehra, 2013^31^ | NR | NR | Convenience | Male | RPR | 6,585 | 0.68 |
| Lifetime Syphilis Infection | |  |  |  |  |  |  |  |
| Algeria | Djoudi, 2023^32^ | 2010-2019 | Bejaija | Convenience | Mix | ELISA | 140,168 | 0.13 |
| Afghanistan | Hashemi, 2022^33^ | 2015-2020 | NR | Convenience | Male | ICT/ ICS | 956,509 | 0.55 |
| Afghanistan | Mansoor, 2013^34^ | 2010-2011 | NR | Convenience | NR | POCT & TPPA | 1,612 | 0.19 |
| Egypt | Abdel Messih, 2014^35^ | 2010-2011 | Cairo | Convenience | Female | ELISA | 3,200 | 0.19 |
| Egypt | Abdel Messih, 2014^35^ | 2010-2011 | Cairo | Convenience | Male | ELISA | 13,918 | 0.27 |
| Egypt | Ashour, 2009^36^ | 2006-2008 | Cairo | Convenience | NR | EIA | 515,758 | 0.13 |
| Egypt | Farouk, 2015^37^ | 2013-2014 | Cairo | Convenience | NR | ELISA | 33,921 | 0.06 |
| Iran | Niazkar, 2020^38^ | 2004-2014 | Kbtc | Convenience | Mix | EIA | 198,501 | 0.00 |
| Iraq | Abdullah, 2016^39^ | 2016 | Basra | Convenience | Mix | TPHA | 6,536 | 0.28 |
| Iraq | Hassan, 2020^40^ | 2018 | Baghdad | Convenience | Mix | ELISA | 178,966 | 0.69 |
| Iraq | Hassan, 2020^41^ | 2018 | Karbala | Convenience | Mix | ELISA & TPHA | 30,716 | 0.76 |
| Iraq | Saleh, 2010^42^ | 2009-2010 | Baghdad | Convenience | Mix | TPHA | 8,147 | 1.31 |
| Iraq | Mohammed, 2023^43^ | 2019-2021 | Basra | Convenience | Mix | ELISA | 197,898 | 0.4 |
| Jordan | Hroob, 2020^44^ | 2013-2018 | Amman | Convenience | Mix | ICT/ ICS | 365,029 | 0.02 |
| Jordan | Souan, 2016^45^ | 2009-2013 | Amman | Convenience | NR | ICT/ ICS | 62,933 | 0.00 |
| KSA | Al-Ahmadi, 2023^46^ | 2020-2022 | Madinah | Convenience | Mix | CMIA & TPHA | 104,538 | 0.05 |
| KSA | Almaiman, 2018^47^ | 2013-2016 | NR | Convenience | Mix | ELISA | 9,460 | 0.21 |
| KSA | Alsughayyir, 2022^48^ | 2020 | NR | Convenience | Mix | CIA | 375,218 | 0.13 |
| KSA | Elyamany, 2016^49^ | 2006-2015 | Riyadh | Convenience | Mix | ELISA | 239,330 | 0.03 |
| KSA | Hossain, 1986^12^ | NR | NR | Convenience | Mix | TPHA | 1,263 | 3.01 |
| KSA | Kabrah, 2023^50^ | 2015-2018 | Mecca | Convenience | Mix | CMIA | 5,473 | 0.38 |
| KSA | Kilany, 2015^51^ | 2012-2013 | NR | Convenience | Mix | ELISA | 7,267 | 0.03 |
| KSA | Sarah, 2016^21^ | 2014-2015 | NR | Convenience | Mix | TPHA | 361 | 0.83 |
| KSA | Shaikh, 2024^52^ | 2020-2022 | Abha | Convenience | Mix | CMIA | 10,095 | 0.45 |
| Libya | Ali, 2014^53^ | 2008-2015 | Northeast Of Libya | Convenience | Mix | ELISA | 78,987 | 0.01 |
| Morocco | Laouina, 2016^54^ | 2015 | Rabat | Convenience | Mix | TPHA | 57,711 | 1.50 |
| Pakistan | Ahmad, 2019^55^ | 2012-2016 | Lahore | Convenience | Mix | ICT/ ICS | 79,764 | 1.14 |
| Pakistan | Ahmad, 2020^56^ | 2019 | Lahore | Convenience | Mix | EIA | 45,127 | 12.50 |
| Pakistan | Ali, 2023^57^ | 2020-2022 | Karachi | Convenience | Mix | CIA | 59,708 | 1.20 |
| Pakistan | Amin, 2019^58^ | 2019 | NR | Convenience | Mix | CMIA | 9,152 | 0.68 |
| Pakistan | Arshad, 2016^59^ | 2013-2015 | NR | Convenience | Female | CMIA | 45 | 0.00 |
| Pakistan | Arshad, 2016^59^ | 2013-2015 | NR | Convenience | Male | CMIA | 16,543 | 2.16 |
| Pakistan | Awan, 2018^60^ | 2015-2017 | NR | Convenience | NR | EIA | 30,470 | 0.75 |
| Pakistan | Batool, 2017^61^ | 2008-2014 | Peshawar | Convenience | Mix | CIA | 41,033 | 0.88 |
| Pakistan | Bhatti, 2007^62^ | 2003-2005 | Rawalpindi | Convenience | NR | TPHA | 94,177 | 0.75 |
| Pakistan | Bhatti, 2022^63^ | 2015-2019 | Islamabad | Convenience | Mix | CIA | 51,695 | 0.83 |
| Pakistan | Butt, 2018^64^ | 2012-2016 | NR | Convenience | Mix | ICT/ ICS | 79,774 | 1.10 |
| Pakistan | Ghazanfar, 2022^65^ | 2015-2018 | Rawalpindi | Convenience | Mix | ICT/ ICS | 216,190 | 0.93 |
| Pakistan | Hussain, 2015^66^ | 2013 | NR | Convenience | Mix | ICT/ ICS | 48,020 | 0.07 |
| Pakistan | Jamal, 2023^67^ | 2017-2021 | Urban Sindh (Karachi) | Convenience | Mix | CMIA | 72,501 | 0.8 |
| Pakistan | Jamal, 2023^67^ | 2017-2021 | Semi-urban Sindh | Convenience | Mix | CMIA | 113,730 | 3.36 |
| Pakistan | Jamal, 2023^67^ | 2017-2021 | Urban Oubjab (Lahore) | Convenience | Mix | CMIA | 6,841 | 0.06 |
| Pakistan | Jamal, 2023^67^ | 2017-2021 | Semi-Urban Punjab | Convenience | Mix | CMIA | 399,198 | 2.06 |
| Pakistan | Jiskani, 2019^68^ | 2016-2018 | NR | Convenience | Mix | POCT, unspecified | 2,002 | 2.45 |
| Pakistan | Mudasar, 2020^69^ | 2017 | Faisalabad | Convenience | Mix | EIA | 29,167 | 2.43 |
| Pakistan | Nawaz, 2021^70^ | 2018-2019 | NR | Convenience | Mix | ICT/ ICS | 1,200 | 3.92 |
| Pakistan | Naz, 2018^71^ | 2013-2014 | NR | Convenience | Mix | CIA | 14,652 | 0.01 |
| Pakistan | Nazir, 2013^72^ | 2012 | Lahore | Convenience | Mix | EIA | 14,352 | 3.13 |
| Pakistan | Niazi, 2015^73^ | 2012-2013 | Rawalpandi | Convenience | Mix | POCT, unspecified | 56,772 | 0.98 |
| Pakistan | Noorulamin, 2016^74^ | 2014-2015 | Rawalpandi | Convenience | Mix | ICT/ ICS | 6,053 | 1.47 |
| Pakistan | Rahat, 2022^75^ | NR | Karachi | Convenience | Male | ELISA | 515 | 1.70 |
| Pakistan | Rauf, 2019^76^ | 2018-2019 | NR | Convenience | Mix | EIA | 6,594 | 1.11 |
| Pakistan | Saba, 2021^77^ | 2016-2020 | Khyber Pakhtunkhwa | Convenience | Mix | CIA | 41,817 | 0.91 |
| Pakistan | Sabir, 2023^78^ | 2017-2021 | Rawalpindi | Convenience | Mix | CMIA/NAAT | 308,767 | 0.9 |
| Pakistan | Saeed, 2017^79^ | 2014-2015 | Lahore | Convenience | Mix | ICT/ ICS | 18,274 | 1.55 |
| Pakistan | Shah, 2023^80^ | 2020-2021 | Khyber Pakhtunkhwa | Convenience | Mix | ELISA | 6,311 | 0.3 |
| Pakistan | Shah, 2023^81^ | 2020-2021 | Khyber Pakhtunkhwa | Convenience | Mix | ELISA | 6,311 | 0.32 |
| Pakistan | Siddiqui, 2019^82^ | 2016-2017 | Islamabad | Systematic random sampling | Mix | POCT, unspecified | 847 | 0.35 |
| Pakistan | Sultan Sadia, 2017^83^ | 2015-2016 | Karachi | Convenience | Mix | CIA | 16,957 | 1.69 |
| Pakistan | Zafar, 2020^84^ | 2017-2018 | Lahore | Convenience | Mix | EIA | 11,839 | 0.55 |
| Pakistan | Zameer, 2017^85^ | 2015-2016 | NR | Convenience | Male | ICT/ ICS | 10,048 | 2.08 |
| Qatar | Aabdien, 2020^86^ | 2013-2017 | Doha | Convenience | Mix | CMIA | 190,509 | 0.43 |
| Somalia | Nur, 2000^87^ | 1995 | Mogadishu | Convenience | Mix | EIA | 54 | 1.85 |
| Sudan | Ahmed, 2020^88^ | 2017 | White Nile State | Convenience | Male | ICT/ ICS | 10,897 | 5.73 |
| Sudan | Mohammed, 2019^89^ | 2017 | NR | Convenience | Mix | ICT/ ICS | 513 | 6.60 |
| Tunisia | Ben Jemia, 2014^90^ | 2010 | NR | Convenience | Mix | TPHA | 19,783 | 0.13 |
| UAE | Raouf, 2010^91^ | 2009 | NR | Convenience | Mix | ELISA | 12,195 | 0.20 |
| UAE | Raouf, 2010^91^ | 2005 | NR | Convenience | Mix | ELISA | 6,839 | 0.40 |
| Yemen | Alharazi, 2022^92^ | NR | NR | Convenience | Mix | ICT/ ICS | 16,367 | 2.40 |
| Yemen | Ghaleb, 2019^93^ | 2018 | Sana’A | Convenience | Male | ICT/ ICS | 17,913 | 1.00 |
| Yemen | Saghir, 2012^94^ | 2008-2010 | Hodeidah | Convenience | Male | ICT/ ICS | 1,483 | 0.34 |
| Unclear Infection Type | |  |  |  |  |  |  |  |
| Algeria | Talbi, 2022^95^ | 2008-2017 | NR | Convenience | Mix | NR | 10,300 | 0.23 |
| Egypt | Ahmed, 2009^96^ | NR | NR | Convenience | Mix | NR | 760 | 0.13 |
| Egypt | Hussein, 2014^97^ | 2006-2012 | Cairo | Convenience | NR | NR | 308,762 | 0.13 |
| KSA | Anwar, 2014^98^ | 2010-2013 | NR | Convenience | Mix | NR | 39,198 | 0.06 |
| Oman | Al-Harthy, 2016^99^ | 2015 | NR | Convenience | Mix | NR | 26,173 | 0.14 |
| Pakistan | Hafeez, 2016^100^ | 2015 | Islamabad | Convenience | NR | NR | 3,988 | 1.78 |
| Pakistan | Kamran, 2014^101^ | NR | Rawalpindi | Convenience | Female | NR | 50 | 0.00 |
| Pakistan | Kamran, 2014^101^ | NR | Rawalpindi | Convenience | Male | NR | 250 | 2.40 |
| Pakistan | Rasheed, 2020^102^ | 2017-2019 | Punjab | Convenience | Mix | NR | 3,010 | 4.02 |
| Pakistan | Soomro, 2012^103^ | 2008-2010 | Sukkur | Convenience | NR | NR | 7,085 | 0.51 |
| Pakistan | Wazeer, 2020^104^ | 2018 | NR | Convenience | Mix | Not applicable | 2,449,308 | 0.72 |
| Pakistan | Zaffar, 2013^105^ | 2007-2011 | NR | Convenience | NR | NR | 246,611 | 0.97 |
| Sudan | Sayedahmed, 2023^106^ | 2021-2022 | Several Cities | Convenience | Mix | NR | 2,981 | 3.70 |
| Sudan | Yousif, 2015^107^ | 2012-2014 | Khartoum | Convenience | NR | NR | 126,861 | 2.60 |

^*^ The table is sorted by country then by year(s) of data collection.

**^✝^** Indicates a positive reaction on the assay described.

CIA: Chemiluminescence immunoassay, CMIA: Chemiluminescent microparticle immunoassay, EIA: Enzyme immunoassay, ELISA: Enzyme-linked immunosorbent assay, FTA-ABS: Fluorescent treponemal antibody absorption assay, ICS: Immunochromatographic strip, ICT: Immunochromatographic test, KSA: Kingdom of Saudi Arabia, NR: Not reported, POCT: Point of care test, RPR: Rapid plasma reagin, TPHA: *T. pallidum* hemagglutination assay, TPPA: *T. pallidum* particle agglutination, UAE: United Arab Emirates, VDRL: Venereal disease research laboratory.

# Table S4. Syphilis studies among pregnant women in the Middle East and North Africa, stratified by infection type.^*^

| **Country** | **Citation** | **Collection year** | **City** | **Sampling** | **Setting** | **Assay^✝^** | **Tested (N)** | **Prevalence**  **(%)** |
| --- | --- | --- | --- | --- | --- | --- | --- | --- |
| Probable Current Syphilis Infection | |  |  |  |  |  |  |  |
| Afghanistan | Todd, 2008^108^ | 2006 | Kabul | Convenience | Hospital | POCT & RPR & TPPA | 4,452 | 0.00 |
| Bahrain | Yousif, 1991^109^ | 1989-1990 | Salmaniya | Convenience | ANC clinic | VDRL & TPHA | 1,000 | 0.90 |
| Iran | Attary, 2014^110^ | NR | Tehran | Convenience | GYN clinic | VDRL & FTA-ABS | 605 | 0.00 |
| Iraq | Hassan, 2015^111^ | 2012-2013 | Baghdad | Convenience | Hospital | VDRL & TPHA | 400 | 2.25 |
| KSA | Hossain, 1986^12^ | NR | NR | Convenience | NR | VDRL & TPHA & FTA-ABS | 4,361 | 0.62 |
| KSA | Lumbiganon, 2002^112^ | NR | Jeddah | Convenience | ANC clinic | (RPR or VDRL) &FTA-ABS | 24,269 | 0.89 |
| KSA | Shakoor, 2004^113^ | 2002-2003 | Riyadh | Convenience | ANC clinic | RPR & TPHA | 3,270 | 0.03 |
| Pakistan | Shah, 2002^114^ | NR | NR | Convenience | ANC clinic | RPR & TPHA | 1,500 | 0.53 |
| Pakistan | Shah, 2011^115^ | 2007 | Karachi | Convenience | ANC clinic | RPR & TPHA | 800 | 0.88 |
| Somalia | Abdalla, 2010^116^ | 2004 | Hargeisa, Borama, Berbera | Convenience | Hospital | RPR & TPHA | 1,559 | 1.35 |
| Somalia | Jama, 1987^117^ | 1985-1986 | Mogadishu | Convenience | Mix | VDRL & TPHA | 67 | 2.99 |
| South Sudan | Lodiongo, 2018^118^ | 2018 | Juba | Convenience | Primary health care | TPHA | 442 | 4.98 |
| Sudan | Nagi, 2008^119^ | 2005 | NR | Convenience | ANC clinic | TPHA | 100 | 9.00 |
| Sudan | Ortashi, 2004^120^ | 1999 | Khartoum | Convenience | ANC clinic | RPR & TPHA | 151 | 7.28 |
| Tunisia | Znazen, 2013^121^ | NR | Sfax | Convenience | Family planning clinic | RPR & TPHA | 220 | 0.00 |
| Tunisia | Ayachi, 1997^122^ | 1992-1994 | NR | Convenience | NR | VDRL & TPHA | 111 | 0.90 |
| Possible Current Infection, Unspecified | | |  |  |  |  |  |  |
| Iran | Ayat, 2006^123^ | 2004-2005 | Yazd | Convenience | ANC clinic | RPR | 1,484 | 0.00 |
| Iran | Josheghani, 2015^124^ | 2010-2012 | Kashan | Convenience | Prenatal screening laboratory | RPR | 80 | 0.00 |
| Iran | Motamedifar, 2013^125^ | 2011-2012 | Shiraz | NR | ANC clinic | RPR | 1,100 | 1.36 |
| South Sudan | Lodiongo, 2019^118^ | 2018 | Juba | Convenience | Primary health care center | RPR | 442 | 3.17 |
| Sudan | Emmanuel, 2010^126^ | 2009 | Juba | Convenience | ANC clinic | RPR | 231 | 22.08 |
| Sudan | Nagi, 2008^119^ | 2005 | NR | Convenience | ANC clinic | RPR | 100 | 12.00 |
| Lifetime Syphilis Infection | |  |  |  |  |  |  |  |
| Algeria | Aidaoui, 2008^127^ | 2003-2004 | NR | Convenience | Hospital | TPHA | 3,032 | 0.26 |
| KSA | Hossain, 1986^12^ | NR | NR | Convenience | NR | TPHA | 4,361 | 0.89 |
| Pakistan | Chaudry, 2021^128^ | 2019 | Rawalpindi | Convenience | NR | ICT/ ICS | 503 | 0.00 |
| Pakistan | Mccauley, 2018^129^ | 2014-2015 | Punjab | Simple random sampling | Hospital | ICT/ ICS | 3,287 | 0.00 |
| Somalia | Jama, 1987^117^ | 1985-1986 | Mogadishu | Convenience | Mix | TPHA | 67 | 0.00 |
| Sudan | Abdelrahim, 2017^130^ | NR | Khartoum | Convenience | ANC clinic | POCT, unspecified | 377 | 5.04 |
| Sudan | Elkheir, 2018^131^ | 2016-2017 | El Obeid | Convenience | ANC clinic | ICT/ ICS | 444 | 7.43 |
| Sudan | Sudan NAP, 2014^132^ | 2010 | Several cities | Convenience | ANC clinic | ICT/ ICS | 11,889 | 2.35 |
| Sudan | Sudan NAP, 2010^133^ | 2009 | Several cities | Convenience | ANC clinic | ICT/ ICS | 12,231 | 1.32 |
| Sudan | Emmanuel, 2010^126^ | 2009 | Juba | Convenience | ANC clinic | TPHA | 231 | 34.20 |
| Tunisia | Jebara, 1993^134^ | NR | Monastir | Convenience | Maternity ward | TPHA | 1,975 | 0.56 |
| Unclear Infection Type | |  |  |  |  |  |  |  |
| Djibouti | WHO, 2011^135^ | 2009 | NR | Unclear | ANC clinic | NR | NR | 0.20 |
| Djibouti | WHO, 2011^135^ | 2007 | NR | Unclear | ANC clinic | NR | NR | 0.80 |
| Iraq | WHO, 2011^135^ | 2010 | NR | Unclear | ANC clinic | NR | 57,134 | 0.00 |
| Iraq | WHO, 2011^135^ | 2009 | NR | Unclear | ANC clinic | NR | 22,486 | 0.00 |
| Iraq | WHO, 2011^135^ | 2008 | NR | Unclear | ANC clinic | NR | 17,871 | 0.00 |
| Iraq | WHO, 2011^135^ | 2007 | NR | Unclear | ANC clinic | NR | 17,677 | 0.00 |
| Morocco | WHO, 2011^135^ | 2010 | NR | Unclear | ANC clinic | NR | NR | 1.11 |
| Morocco | WHO, 2011^135^ | 2009 | NR | Unclear | ANC clinic | NR | 15,547 | 1.10 |
| Morocco | WHO, 2011^135^ | 2007 | NR | Unclear | ANC clinic | NR | 16,442 | 1.01 |
| Morocco | Safir, 2012^136^ | 2006-2011 | Rabat | Convenience | ANC clinic | NR | 3,670 | 0.60 |
| Oman | WHO, 2011^135^ | 2008 | NR | Unclear | ANC clinic | NR | NR | 0.10 |
| Oman | WHO, 2011^135^ | 2007 | NR | Unclear | ANC clinic | NR | NR | 0.20 |
| Palestine | WHO, 2011^135^ | 2010 | NR | Unclear | ANC clinic | NR | NR | 0.00 |
| Palestine | WHO, 2011^135^ | 2009 | NR | Unclear | ANC clinic | NR | NR | 0.00 |
| Pakistan | Gul, 2005^137^ | NR | Peshawar | Simple random sampling | ANC clinic | NR | 400 | 0.75 |
| Somalia | WHO, 2011^135^ | 2010 | 9 sites | Unclear | ANC clinic | NR | 192 | 1.40 |
| Somalia | WHO, 2011^135^ | 2007 | Puntland | Unclear | ANC clinic | NR | NR | 1.90 |
| Somalia | WHO, 2011^135^ | 2007 | Somaliland | Unclear | ANC clinic | NR | NR | 2.00 |
| South Sudan | WHO, 2011^135^ | 2009 | 10 states | Unclear | ANC clinic | NR | NR | 9.90 |
| South Sudan | WHO, 2011^135^ | 2007 | 6 states | Unclear | ANC clinic | NR | NR | 11.20 |
| Yemen | WHO, 2011^135^ | 2010 | Several cities | Unclear | ANC clinic | NR | NR | 0.40 |
| Yemen | WHO, 2011^135^ | 2007 | 2 cities | Unclear | ANC clinic | NR | NR | 2.00 |

^*^ The table is sorted by country then by year(s) of data collection.

**^✝^** Indicates a positive reaction on the assay described.

ANC: Antenatal care, FTA-ABS: Fluorescent treponemal antibody absorption assay, GYN: Gynecology, ICS: Immunochromatographic strip, ICT: Immunochromatographic test, KSA: Kingdom of Saudi Arabia, NAP: National Aids Control Program, NR: Not reported, POCT: Point of care test, RPR: Rapid plasma reagin, TPHA: *T. pallidum* hemagglutination assay, TPPA: *T. pallidum* particle agglutination, VDRL: Venereal disease research laboratory, WHO: World Health Organization.

# Table S5. Syphilis studies among other general population groups in the Middle East and North Africa, stratified by infection type.^*^

| **Country** | **Citation** | **Collection year** | **City** | **Sampling** | **Setting** | **Population** | **Sex** | **Assay^✝^** | **Tested (N)** | **Prevalence**  **(%)** |
| --- | --- | --- | --- | --- | --- | --- | --- | --- | --- | --- |
| Probable Current Syphilis Infection | |  |  |  |  |  |  |  |  |  |
| Iran | Orang, 2002^138^ | NR | Mazandaran | Targeted | Disability center | Disabled | Mix | RPR & VDRL & FTA-ABS | 325 | 6.15 |
| Jordan | Mahafzah, 2008^139^ | 2003-2005 | NR | Non-probability based | Family planning clinic | Sexually active women | Female | RPR & TPHA | 186 | 0.00 |
| Lebanon | Zaki, 2021^140^ | 2015-2019 | Beirut | Convenience | Sexual health clinic | Sexually active women | Female | ICT & VDRL | 1,498 | 0.07 |
| Lebanon | Deeb, 2003^141^ | 1998 | Nabi Sheet | Simple random sampling | Home | General population | Female | VDRL & FTA-ABS | 495 | 0.00 |
| Morocco | Khallouk, 2012^142^ | 2008-2011 | Casablanca | Convenience | Hospital | Patients | Mix | VDRL & TPHA | 12,884 | 10.80 |
| Pakistan | Mir, 2009^143^ | 2007 | Several Cities | Stratified sampling | Community | General population | Male | RPR & TPHA | 2,383 | 1.34 |
| Qatar | Nasrallah, 2023^144^ | 2019 | NR | Convenience | NR | General population | Mix | RPR & CIA | 97,298 | 0.07 |
| Somalia | Ismail, 1990^145^ | 1987 | Jambaluul | Convenience | Community | General population | Female | VDRL & TPHA | 200 | 16.00 |
| Somalia | Ismail, 1990^145^ | 1987 | Jambaluul | Convenience | Community | General population | Male | VDRL & TPHA | 187 | 4.28 |
| Somalia | Jama, 1987^117^ | 1985-1986 | Mogadishu | Convenience | Mix | Educated women | Female | VDRL & TPHA | 71 | 0.00 |
| Sudan | Kafi, 2000^146^ | NR | Khartom | Systematic random sampling | Primary health care center | General population | Female | VDRL & TPHA | 338 | 0.89 |
| Tunisia | Ayachi, 1997^122^ | 1992-1994 | NR | Convenience | NR | Sexually active women | Female | VDRL & TPHA | 456 | 0.66 |
| Tunisia | Zribi, 2008^147^ | 2003-2004 | NR | Convenience | Primary health care | General population | Female | RPR & TPHA | 116 | 1.72 |
| UAE | Nsanze, 1996^148^ | 1993-1994 | Al Ain | Convenience | Outpatient clinic | Patients | Mix | RPR & TPHA | 1,084 | 4.34 |
| Possible Current Infection, Unspecified | | |  |  |  |  |  |  |  |  |
| Afghanistan | Nassery, 1971^149^ | 1970 | Waras | Convenience | NGO clinic | Patients | Mix | VDRL | 170 | 21.76 |
| Algeria | Cirera, 1967^150^ | 1965 | NR | NR | Community | Nomades | Mix | Kolmer test & Kline test | 61 | 8.20 |
| Algeria | Cirera, 1967^150^ | 1965 | NR | NR | Community | Sedentary black people | Mix | Kolmer test & Kline test | 375 | 4.50 |
| Algeria | Cirera, 1967^150^ | 1965 | NR | NR | Community | Semi nomade population | Mix | Kolmer test & Kline test | 54 | 1.85 |
| Djibouti | Rodier, 1993^151^ | 1990 | NR | Stratified sampling | Educational institute | Students | Mix | RPR | 294 | 0.00 |
| Egypt | El-Sayed, 1996^152^ | 1994 | NR | Mix | Tourist area | Tourism workers | Mix | RPR | 740 | 0.27 |
| Egypt | Van Peenen, 1963^153^ | 1962 | West Desert of Egypt | Convenience | Informal settlements | Arab Bedouin Tribes | Mix | VDRL | 572 | 0.70 |
| Iran | Hodgson, 1951^154^ | 1949-1950 | Abadan | Convenience | Work place | Artisan recruits | Male | Berger & Harrison-Wyler test | 217 | 11.06 |
| Iran | Hodgson, 1951^154^ | 1949-1950 | Abadan | Convenience | Work place | Artisan recruits | Male | Berger & Harrison-Wyler test | 219 | 8.22 |
| Iran | Hodgson, 1951^154^ | 1949-1950 | Abadan | Convenience | Work place | Artisan recruits | Male | Berger & Harrison-Wyler test | 432 | 2.55 |
| Iran | Hodgson, 1951^154^ | 1949-1950 | Abadan | Convenience | Work place | Artisan recruits | Male | Berger & Harrison-Wyler test | 857 | 7.58 |
| KSA | El Ghoroury, 1954^155^ | 1952-1953 | Asir Province | Convenience | NR | General population | Mix | VDRL& Meinicke | 2,359 | 22.17 |
| Morocco | Zahraoui-Mehadji, 2004^156^ | 2001 | Casablanca | Simple random sampling | Work place | Street barber | Male | VDRL | 150 | 4.00 |
| Pakistan | Hyder, 2010^157^ | 2007-2009 | NR | Convenience | Private laboratory | General population | Male | VDRL | 32,420 | 3.20 |
| Pakistan | Khan, 2010^158^ | 1991-2008 | NR | Convenience | Community | Blood Donors | Mix | RPR | 91,335 | 3.26 |
| Pakistan | Khan, 2004^159^ | 1998-2002 | Islamabad | Targeted | Hospital | General population | Mix | RPR | 47,538 | 0.67 |
| Pakistan | Qadeer, 2013^27^ | 2007-2012 | Lahore | Convenience | Prison | Students | NR | RPR | 5,000 | 10.70 |
| South Sudan | Ahmed, 1985^160^ | NR | Juba | Convenience | Hospital | General population | Female | RPR | NR | 37.52 |
| South Sudan | Ahmed, 1985^160^ | NR | Juba | Convenience | Hospital | General population | Male | RPR | NR | 35.20 |
| Lifetime Syphilis Infection | |  |  |  |  |  |  |  |  |  |
| Egypt | Younis, 1993^161^ | 1989-1990 | Giza | Stratified sampling | Community | Ever married, nonpregnant women | Female | TPHA | 496 | 0.81 |
| KSA | Memish, 2015^162^ | 2012-2013 | NR | Convenience | Primary health care | General population | Mix | CMIA | 4,906 | 0.55 |
| KSA | Filemban, 2015^163^ | 2013-2014 | Several Cities | Multi-stage sampling | Hospital | Patients | Mix | POCT, unspecified | 3,994 | 0.30 |
| Morocco | Zahraoui-Mehadji, 2004^156^ | 2001 | Casablanca | Simple random sampling | Work place | Street barber | Male | TPHA | 150 | 7.00 |
| Somalia | Ismail, 1990^145^ | 1987 | Jambaluul | Convenience | Community | General population | Female | TPHA | 200 | 6.50 |
| Somalia | Ismail, 1990^145^ | 1987 | Jambaluul | Convenience | Community | General population | Male | TPHA | 187 | 19.25 |
| Somalia | Jama, 1987^117^ | 1985-1986 | Mogadishu | Convenience | Mix | Educated women | Female | TPHA | 71 | 0.00 |
| Unclear Infection Type | |  |  |  |  |  |  |  |  |  |
| South Sudan | Kaiser, 2004^164^ | 2002-2003 | Rumbek | Multi-stage cluster sampling | Home | General population | Mix | NR | 966 | 2.80 |
| South Sudan | Kaiser, 2004^164^ | 2002-2003 | Yei | Multi-stage cluster sampling | Home | General population | Mix | NR | 1,054 | 0.66 |
| South Sudan | Sube, 2020^165^ | 2017 | Juba | Unclear | NR | Students | Mix | NR | 158 | 13.30 |

^*^ The table is sorted by country then by year(s) of data collection.

**^✝^** Indicates a positive reaction on the assay described.

CIA: Chemiluminescence immunoassay, CMIA: Chemiluminescent microparticle immunoassay, ELISA: Enzyme-linked immunosorbent assay, FTA-ABS: Fluorescent treponemal antibody absorption assay, ICS: Immunochromatographic strip, ICT: Immunochromatographic test, IDP: Internally displaced populations, KSA: Kingdom of Saudi Arabia, NGO: Non-governmental organization, NR: Not reported, POCT: Point of care test, RPR: Rapid plasma reagin, TPHA: T. pallidum hemagglutination assay, TPPA: *T. pallidum* particle agglutination, UAE: United Arab Emirates, VDRL: Venereal disease research laboratory.

# Table S6. Syphilis studies among populations at intermediate risk in the Middle East and North Africa, stratified by infection type.^*^

| **Country** | **Citation** | **Collection year** | **City** | **Sampling** | **Setting** | **Population** | **Sex** | **Assay^✝^** | **Tested (N)** | **Prevalence**  **(%)** |
| --- | --- | --- | --- | --- | --- | --- | --- | --- | --- | --- |
| Probable Current Syphilis Infection | |  |  |  |  |  |  |  |  |  |
| Afghanistan | Todd, 2012^166^ | 2010-2011 | Kabul | Convenience | Military camp | Military recruits/ personnel | Male | RPR & TPPA | 4,729 | 0.65 |
| Bahrain | Yousif, 1994^167^ | NR | NR | Convenience | Community | HIV patients | NR | VDRL & TPHA | 76 | 3.95 |
| Iran | Hashemi-Shahri, 2016^168^ | 2000-2015 | Zahedan | Convenience | Hospital | HIV patients | Mix | VDRL & FTA-ABS | 41 | 12.20 |
| Iran | Vahdani, 2006^169^ | NR | Tehran | Targeted | Center for street children | Street children | Mix | RPR & FTA-ABS | 102 | 0.00 |
| Jordan | Yanni, 2013^170^ | 2007-2009 | NR | Convenience | NGO clinic | Refugees/IDPs | Mix | RPR & (FTA-ABS or TPPA) | 9,518 | 0.15 |
| KSA | Chowdhury, 1982^171^ | 1978-1980 | Riyadh | Unclear/missing | GYN clinic | Women seeking gynecological consultation | Female | VDRL & TPHA &  FTA-ABS | 299 | 1.00 |
| KSA | Hamdi, 1997^172^ | 1987-1994 | Jeddah | Convenience | Work place | Migrant workers | Mix | RPR & VDRL & FTA-ABS | 1,648 | 23.80 |
| Morocco | Bourouache, 2019^173^ | 2011-2016 | Agadir | NR | Hospital | HIV patients | Mix | VDRL & TPHA | 2,808 | 17.13 |
| Morocco | El Ghrari, 2007^174^ | 2004 | Casablanca | Convenience | Prison | Prisoners | Female | VDRL & TPHA | 217 | 18.89 |
| Pakistan | Altaf, 2007^175^ | 2003 | Karachi | Convenience | Rehabilitation center | PWID | NR | RPR & TPHA | 161 | 13.04 |
| Pakistan | Baqi, 1998^176^ | 1994 | Sindh | Convenience | Medical center | PWID | Male | VDRL & FTA-ABS | 272 | 6.62 |
| Pakistan | Faisel, 2006^177^ | NR | Lahore | Stratified sampling | Community | Migrant workers | Male | RPR & TPHA | 188 | 1.60 |
| Pakistan | Laghari, 2014^178^ | 2012-2013 | Sukkar | Targeted | GYN clinic | Women seeking gynecological consultation | Female | VDRL & FTA-ABS | 256 | 1.95 |
| Pakistan | Pakistan NAP, 2005^179^ | 2004 | Karachi | Systematic random sampling | Community | Truck drivers | Male | RPR & TPHA | 396 | 4.04 |
| Pakistan | Pakistan NAP, 2005^179^ | 2004 | Lahore | Systematic random sampling | Community | Truck drivers | Male | RPR & TPHA | 380 | 1.05 |
| Pakistan | Pakistan NAP, 2005^179^ | 2004 | Karachi | Time-location sampling | Community | PWID | Male | RPR & TPHA | 395 | 18.23 |
| Pakistan | Pakistan NAP, 2005^179^ | 2004 | Lahore | Time-location sampling | Community | PWID | Male | RPR & TPHA | 381 | 3.94 |
| Pakistan | Kazi, 2010^180^ | 2007-2008 | Karachi | Probability-based | Prison | Hospitalized- inmates | Male | RPR & TPHA | 53 | 9.40 |
| Pakistan | Kazi, 2010^180^ | 2007-2008 | Karachi | Probability-based | Prison | Prisoners | Male | RPR & TPHA | 303 | 8.90 |
| Pakistan | Platt, 2009^181^ | 2007 | Abbottabad | RDS | Community | PWID | Mix | RPR & TPHA | 102 | 3.92 |
| Pakistan | Platt, 2009^181^ | 2007 | Rawalpindi | RDS | Community | PWID | Mix | RPR & TPHA | 302 | 7.62 |
| Somalia | IOM & WHO, 2017^182^ | 2017 | Bossaso | Time-location sampling | Community | Port workers | Male | RPR & ICS | 289 | 0.69 |
| Somalia | IOM & WHO, 2017^183^ | 2017 | Mogadishu | Time-location sampling | Community | Port workers | Male | RPR & ICS | 286 | 0.70 |
| Somalia | IOM & WHO, 2017^182^ | 2017 | Bossaso | Time-location sampling | Community | Uniformed personnel (military, police) | Male | RPR & ICS | 287 | 0.70 |
| Somalia | IOM & WHO, 2017^182^ | 2017 | Mogadishu | Time-location sampling | Community | Uniformed personnel (military, police) | Male | RPR & ICS | 287 | 0.00 |
| Somalia | IOM & WHO, 2017^183^ | 2017 | Hargeisa | Time-location sampling | Community | Uniformed personnel (military, police) | Male | RPR & ICS | 288 | 0.00 |
| Somalia | IOM & WHO, 2017^182^ | 2017 | Bossaso | Time-location sampling | Community | Truck drivers | Male | RPR & ICS | 287 | 0.35 |
| Somalia | IOM & WHO, 2017^182^ | 2017 | Mogadishu | Time-location sampling | Community | Truck drivers | Male | RPR & ICS | 286 | 0.00 |
| Somalia | IOM & WHO, 2017^183^ | 2017 | Hargeisa | Time-location sampling | Community | Truck drivers | Male | RPR & ICS | 286 | 1.40 |
| Somalia | Watts, 1994^184^ | 1990-1990 | Several Cities | Non-probability based | Mix | Military recruits/ personnel | Male | RPR & FTA-ABS | 79 | 3.80 |
| South Sudan | Kaiser, 2006^185^ | 2002-2003 | Rumbek Town | Multi-stage cluster sampling | Home | Refugees/IDPs | Mix | RPR & TPHA | 966 | 2.80 |
| South Sudan | Kaiser, 2006^185^ | 2002-2003 | Yei Outside Town | Multi-stage cluster sampling | Home | Refugees/IDPs | Mix | RPR & TPHA | 438 | 0.23 |
| South Sudan | Kaiser, 2006^185^ | 2002-2003 | Yei Town | Multi-stage cluster sampling | Home | Refugees/IDPs | Mix | RPR & TPHA | 616 | 0.97 |
| Tunisia | Ayachi, 1997^122^ | 1992-1994 | NR | Convenience | NR | Women seeking gynecological consultation | Female | VDRL & TPHA | 345 | 0.58 |
| Possible Current Infection, Unspecified | | |  |  |  |  |  |  |  |  |
| Iran | Badie, 2013^186^ | 2004-2008 | Tehran | Convenience | Hospital | HIV patients | Mix | VDRL | 450 | 5.33 |
| Iran | Ghassabi, 2018^187^ | 2004-2013 | Shiraz | NR | Educational institute | HIV patients | NR | RPR | 806 | 0.00 |
| Iran | Mohamadi, 2009^188^ | 2008 | Tehran | NR | NR | Military recruits/ personnel | NR | VDRL | 1,041 | 0.10 |
| Iran | Hajiabdolbaghi, 2007^189^ | NR | Tehran | NR | NGO clinic | Individual at risk | Female | RPR | 35 | 0.00 |
| Iran | Jahanbaksh, 2017^190^ | 2012 | Tehran | Convenience | Shelter/  Homeless center | Homeless | Mix | RPR | 542 | 0.55 |
| Iran | Nokhodian, 2012^191^ | 2009 | Isfahan | Convenience | Prison | Prisoners | Female | RPR | 163 | 0.00 |
| Iran | Vahdani, 2009^192^ | 2007 | Tehran | Targeted | Street | Homeless | Male | RPR | 202 | 0.50 |
| KSA | Al-Mughales, 2016^193^ | 2013-2014 | Jeddah | Convenience | HIV clinic | HIV patients | Mix | VDRL | 79 | 1.27 |
| Lifetime Syphilis Infection | |  |  |  |  |  |  |  |  |  |
| Afghanistan | NAP, 2012^194^ | 2012 | Charikar | RDS | Community | PWID | Male | ICT/ ICS | 117 | 2.56 |
| Afghanistan | NAP, 2012^194^ | 2012 | Herat | RDS | Community | PWID | Male | ICT/ ICS | 185 | 2.70 |
| Afghanistan | NAP, 2012^194^ | 2012 | Jalalabad | RDS | Community | PWID | Male | ICT/ ICS | 236 | 7.63 |
| Afghanistan | NAP, 2012^194^ | 2012 | Kabul | RDS | Community | PWID | Male | ICT/ ICS | 269 | 7.81 |
| Afghanistan | NAP, 2012^194^ | 2012 | Mazar | RDS | Community | PWID | Male | ICT/ ICS | 254 | 9.06 |
| Afghanistan | NAP, 2012^194^ | 2012 | Herat | Systematic random sampling | Prison | Prisoners | Male | ICT/ ICS | 351 | 0.85 |
| Afghanistan | NAP, 2012^194^ | 2012 | Kabul | Systematic random sampling | Prison | Prisoners | Male | ICT/ ICS | 368 | 0.82 |
| Afghanistan | NAP, 2012^194^ | 2012 | Torkham | Systematic random sampling | Community | Truck drivers | Male | ICT/ ICS | 378 | 0.26 |
| Afghanistan | NAP, 2010^195^ | 2009 | Herat | RDS | Community | PWID | Male | POCT, unspecified | 159 | 1.89 |
| Afghanistan | NAP, 2010^195^ | 2009 | Kabul | RDS | Community | PWID | Male | POCT, unspecified | 286 | 3.50 |
| Afghanistan | NAP, 2010^195^ | 2009 | Mazar | RDS | Community | PWID | Male | POCT, unspecified | 102 | 16.67 |
| Afghanistan | NAP, 2010^195^ | 2009 | Herat | Systematic random sampling | Prison | Prisoners | Male | POCT, unspecified | 317 | 0.63 |
| Afghanistan | NAP, 2010^195^ | 2009 | Kabul | Systematic random sampling | Prison | Prisoners | Male | POCT, unspecified | 352 | 1.70 |
| Afghanistan | NAP, 2010^195^ | 2009 | Torkham | Systematic random sampling | Community | Truck drivers | Male | POCT, unspecified | 365 | 1.10 |
| Afghanistan | Nasir, 2008^196^ | 2008 | Hirat | Unclear | NR | PWID | Mix | POCT, unspecified | 195 | 3.10 |
| Afghanistan | Ruisenor, 2014^197^ | 2009 | Mazar-I-Sharif, Kabul, Herat | RDS | Community | PWID | Male | POCT, unspecified | 548 | 5.47 |
| Afghanistan | Todd, 2010^198^ | 2005-2008 | Several Cities | Convenience | Rehabilitation center | PWID | Male | CIA & TPPA | 1,036 | 3.86 |
| Afghanistan | Todd, 2011^199^ | 2007-2009 | Kabul | Time-location sampling | Mix | PWID | Male | CIA & TPPA | 483 | 1.20 |
| Afghanistan | Todd, 2012^200^ | 2010-2012 | NR | Unclear | NR | Military recruits/ personnel | Male | POCT, unspecified | 6,849 | 0.48 |
| Djibouti | Djibouti MOPH, 2014^201^ | 2014 | Several areas | Time-location sampling | Community | Truck drivers | Male | TPHA | 503 | 1.60 |
| Iran | Badie, 2013^186^ | 2004-2008 | Tehran | Convenience | Hospital | HIV patients | Mix | FTA-Abs | 450 | 0.44 |
| Lebanon | Abi Zaki, 2020^202^ | 2019 | Beirut | Unclear | Community | Refugees/IDPs | Female | POCT, unspecified | 116 | 0.00 |
| Morocco | El Ghrari, 2007^174^ | 2004 | Casablanca | Convenience | Prison | Prisoners | Female | TPHA | 217 | 23.50 |
| Pakistan | Rehan, 2009^203^ | 2004 | Karachi | Time-location sampling | Shooting galleries | PWID | Male | ELISA | 395 | 18.23 |
| Pakistan | Rehan, 2009^203^ | 2004 | Lahore | Time-location sampling | Shooting galleries | PWID | Male | ELISA | 381 | 13.91 |
| Pakistan | Rehan, 2009^203^ | 2004 | Karachi | Random cluster | Major truck-stops | Truck drivers | Male | ELISA | 396 | 4.04 |
| Pakistan | Rehan, 2009^203^ | 2004 | Lahore | Random cluster | Major truck-stops | Truck drivers | Male | ELISA | 380 | 6.84 |
| Pakistan | Rehman Khan, 2017^204^ | NR | Islamabad | Convenience | Shelter/Homeless center | Refugees/IDPs | NR | CMIA | 240 | 37.50 |
| Somalia | IOM & WHO, 2017^182^ | 2017 | Bossaso | Time-location sampling | Community | Port workers | Male | ICT/ ICS | 289 | 1.73 |
| Somalia | IOM & WHO, 2017^182^ | 2017 | Mogadishu | Time-location sampling | Community | Port workers | Male | ICT/ ICS | 286 | 1.40 |
| Somalia | IOM & WHO, 2017^182^ | 2017 | Bossaso | Time-location sampling | Community | Uniformed personnel (military, police) | Male | ICT/ ICS | 287 | 0.70 |
| Somalia | IOM & WHO, 2017^183^ | 2017 | Hargeisa | Time-location sampling | Community | Uniformed personnel (military, police) | Male | ICT/ ICS | 288 | 0.00 |
| Somalia | IOM & WHO, 2017^182^ | 2017 | Mogadishu | Time-location sampling | Community | Uniformed personnel (military, police) | Male | ICT/ ICS | 287 | 1.74 |
| Somalia | IOM & WHO, 2017^182^ | 2017 | Bossaso | Time-location sampling | Community | Truck drivers | Male | ICT/ ICS | 287 | 0.35 |
| Somalia | IOM & WHO, 2017^183^ | 2017 | Hargeisa | Time-location sampling | Community | Truck drivers | Male | ICT/ ICS | 286 | 1.40 |
| Somalia | IOM & WHO, 2017^182^ | 2017 | Mogadishu | Time-location sampling | Community | Truck drivers | Male | ICT/ ICS | 286 | 1.40 |
| Somalia | Sahiner, 2022^205^ | 2015-2019 | Mogadishu | Convenience | NR | HIV patients | Mix | POCT, unspecified | 58 | 3.45 |
| Tunisia | Teyeb, 2013^206^ | 2011-2012 | NR | Convenience | Hospital | Refugees/IDPs | Mix | POCT, unspecified | 670 | 0.75 |
| UAE | Eihab, 2016^207^ | 2014 | Sharjah | Convenience | Primary health care | Migrant workers | Mix | ELISA & TPHA | 20,670 | 0.51 |
| Unclear Infection Type | |  |  |  |  |  |  |  |  |  |
| Iran | Beheshti, 2014^208^ | NR | Shiraz | NR | Prison | Prisoners | Female | NR | 129 | 6.20 |
| Iran | Faramarzi, 2010^209^ | 2004-2009 | NR | NR | VCT center | HIV patients | Mix | NR | 1,384 | 1.55 |
| KSA | Ledward, 1980^210^ | 1978 | Riyadh | Convenience | GYN clinic | Women seeking gynecological consultation | Female | NR | 41 | 4.88 |
| Morocco | Belglaiaa, 2018^211^ | 2017 | Laayoune | NR | HIV clinic | HIV patients | Female | NR | 115 | 1.74 |
| Pakistan | WHO, 2011^135^ | 2008 | NR | Unclear | Community | PWID | Male | NR | NR | 7.60 |
| Qatar | Al Soub, 2018^212^ | 1984-2014 | NR | Convenience | Hospital | HIV patients | Mix | NR | 120 | 2.50 |

^*^ The table is sorted by country then by year(s) of data collection.

**^✝^** Indicates a positive reaction on the assay described.

CIA: Chemiluminescence immunoassay, ELISA: Enzyme-linked immunosorbent assay, FTA-ABS: Fluorescent treponemal antibody absorption assay, GYN: Gynecology, HIV: Human Immunodeficiency Virus, ICS: Immunochromatographic strip, ICT: Immunochromatographic test, IOM: International Organization for Migration, KSA: Kingdom of Saudi Arabia, MOPH: Ministry of Public Health, NAP: National Aids Control Program, NR: Not reported, POCT: Point of care test, PWID: People who inject drugs, RDS: Respondent driven sampling, RPR: Rapid plasma reagin, TPHA: *T. pallidum* hemagglutination assay, TPPA: *T. pallidum* particle agglutination, VCT : Voluntary counselling and testing, VDRL: Venereal disease research laboratory, WHO: World Health Organization.

# Table S7. Syphilis studies among populations at high risk in the Middle East and North Africa, stratified by infection type.^*^

| **Country** | **Citation** | **Collection year** | **City** | **Sampling** | **Setting** | **Population** | **Assay^✝^** | **Tested (N)** | **Prevalence**  **(%)** |
| --- | --- | --- | --- | --- | --- | --- | --- | --- | --- |
| Probable Current Syphilis Infection | |  |  |  |  |  |  |  |  |
| Iran | Kazerooni, 2014^213^ | 2010-2011 | Shiraz | RDS | Community | FSW | RPR & FTA-ABS | 278 | 0.00 |
| Iran | Shahesmaeili, 2018^214^ | 2015 | 13 Cities | Convenience | Mix | FSW | POCT & RPR & ELISA | 1,318 | 0.46 |
| Morocco | Hancali, 2014^215^ | 2011 | Agadir | RDS | NR | FSW | POCT & VDRL & TPHA | 372 | 21.40 |
| Morocco | Hancali, 2014^215^ | 2007 | Agadir | NR | NR | FSW | POCT & VDRL & TPHA | NR | 13.80 |
| Morocco | Morocco MOPH, 2012^216^ | 2011-2012 | Agadir | RDS | Community | FSW | VDRL & TPHA | 372 | 22.04 |
| Morocco | Morocco MOPH, 2012^216^ | 2011-2012 | Fes | RDS | Community | FSW | VDRL & TPHA | 359 | 19.22 |
| Morocco | Morocco MOPH, 2012^216^ | 2011-2012 | Rabat | RDS | Community | FSW | VDRL & TPHA | 392 | 14.29 |
| Morocco | Morocco MOPH, 2012^216^ | 2011-2012 | Tanger | RDS | Community | FSW | VDRL & TPHA | 318 | 14.47 |
| Morocco | Johnston, 2013^217^ | 2010-2011 | Agadir | RDS | Community | HIV positive MSM | VDRL & TPHA | 19 | 31.60 |
| Morocco | Johnston, 2013^217^ | 2010-2011 | Marrakesh | RDS | Community | HIV positive MSM | VDRL & TPHA | 14 | 56.40 |
| Morocco | Johnston, 2013^217^ | 2010-2011 | Agadir | RDS | Community | MSM | VDRL & TPHA | 323 | 11.46 |
| Morocco | Johnston, 2013^217^ | 2010-2011 | Marrakesh | RDS | Community | MSM | VDRL & TPHA | 346 | 10.40 |
| Morocco | Oukouchoud, 2017^218^ | 2011-2012 | NR | RDS | Sex-work site | FSW | VDRL & TPHA | 1,447 | 17.68 |
| Pakistan | Hawkes, 2009^219^ | 2007 | Abborttabad | RDS | Mix | FSW | RPR & TPHA | 107 | 2.80 |
| Pakistan | Hawkes, 2009^219^ | 2007 | Rawalpindi | RDS | Mix | FSW | RPR & TPHA | 426 | 1.20 |
| Pakistan | Hawkes, 2009^219^ | 2007 | Abborttabad | RDS | Mix | MSW (Bantha) | RPR & TPHA | 83 | 4.90 |
| Pakistan | Hawkes, 2009^219^ | 2007 | Rawalpindi | RDS | Mix | MSW (Bantha) | RPR & TPHA | 195 | 4.70 |
| Pakistan | Hawkes, 2009^219^ | 2007 | Rawalpindi | RDS | Mix | MSW (Khotki) | RPR & TPHA | 364 | 9.60 |
| Pakistan | Hawkes, 2009^219^ | 2007 | Abborttabad | RDS | Mix | MSW (Khusra) | RPR & TPHA | 16 | 37.50 |
| Pakistan | Hawkes, 2009^219^ | 2007 | Rawalpindi | RDS | Mix | MSW (Khusra) | RPR & TPHA | 253 | 48.80 |
| Pakistan | Khan, 2011^220^ | 2007 | Lahore | RDS | Community | FSW | RPR & TPHA | 730 | 4.52 |
| Pakistan | Pakistan NAP, 2005^179^ | 2004 | Karachi | RDS | Community | MSW | RPR & TPHA | 399 | 36.09 |
| Pakistan | Pakistan NAP, 2005^179^ | 2004 | Lahore | RDS | Community | MSW | RPR & TPHA | 387 | 5.68 |
| Pakistan | Pakistan NAP, 2005^179^ | 2004 | Karachi | Systematic random sampling | Community | FSW | RPR & TPHA | 421 | 3.56 |
| Pakistan | Pakistan NAP, 2005^179^ | 2004 | Lahore | Systematic random sampling | Community | FSW | RPR & TPHA | 387 | 6.98 |
| Pakistan | Pakistan NAP, 2005^179^ | 2004 | Karachi | Systematic random sampling | Community | Transgender | RPR & TPHA | 196 | 60.20 |
| Pakistan | Pakistan NAP, 2005^179^ | 2004 | Lahore | Systematic random sampling | Community | Transgender | RPR & TPHA | 192 | 11.46 |
| Pakistan | Baqi, 1998^176^ | 1994 | Sindh | Convenience | Medical institute | FSW | VDRL & FTA-ABS | 81 | 4.94 |
| Pakistan | Baqi, 1999^221^ | 1998 | Karachi | Targeted | Sex-work site | MSW | RPR & TPHA | 208 | 37.02 |
| Pakistan | Shah, 2004^222^ | NR | NR | NR | NR | FSW | VDRL & TPHA | 157 | 11.46 |
| Somalia | Bchir, 1988^223^ | 1987 | Sousse | Convenience | NR | FSW | VDRL & (TPHA or FTA-ABS or EIA) | 42 | 28.57 |
| Somalia | Corwin, 1991^224^ | 1990 | Mogadishu, Merca, Chismayu | Convenience | NR | FSW | VDRL & FTA-ABS | 302 | 35.43 |
| Somalia | IOM & WHO, 2017^182^ | 2017 | Bossaso | RDS | Community | FSW | RPR & ICS | 286 | 1.75 |
| Somalia | IOM & WHO, 2017^183^ | 2017 | Hargeisa | RDS | Community | FSW | RPR & ICS | 287 | 3.48 |
| Somalia | IOM & WHO, 2017^182^ | 2017 | Mogadishu | RDS | Community | FSW | RPR & ICS | 286 | 2.80 |
| Somalia | IOM & WHO, 2017^182^ | 2017 | Mogadishu | RDS | Community | FSW | RPR & ICS | 286 | 6.99 |
| Somalia | IOM, 2017^225^ | 2014 | Hargeisa | RDS | Community | FSW | RPR & ICS | 96 | 2.08 |
| Somalia | Watts, 1994^184^ | 1990-1990 | Several Cities | Non-probability based | Mix | Female | RPR & FTA-ABS | 236 | 30.93 |
| Somalia | Jama Ahmed, 1991^226^ | 1988-1989 | Mogadishu | Targeted | ANC clinic | FSW | (RPR or VDRL) & TPHA | 155 | 47.74 |
| Somalia | Jama, 1987^117^ | 1985-1986 | Mogadishu | Convenience | Military camp | FSW | VDRL & TPHA | 85 | 44.71 |
| South Sudan | Okiria, 2023^227^ | 2017 | NR | RDS | NR | FSW | RPR & ICS | 408 | 9.80 |
| South Sudan | MOPH, 2016^228^ | 2015-2016 | Juba | RDS | Community | FSW | RPR & ICS | 832 | 7.81 |
| South Sudan | MOPH, 2020^229^ | 2019 | Wau | RDS | Community | FSW | NR | 679 | 2.80 |
| South Sudan | MOPH, 2020^230^ | 2019 | Yambio | RDS | Community | FSW | NR | 605 | 1.16 |
| Tunisia | Ayachi, 1997^122^ | 1992-1994 | NR | Convenience | NR | FSW | VDRL & TPHA | 79 | 24.05 |
| Possible Current Infection, Unspecified | | |  |  |  |  |  |  |  |
| Iran | WHO, 2011^135^ | 2010 | Kerman | RDS | Community | FSW | VDRL | 144 | 7.10 |
| Iran | Bashmaq, 2024^231^ | 2019-2020 | Sanandaj | Snow-ball | Mix | FSW | RPR | 100 | 1 |
| Iran | Kassaian, 2012^232^ | 2009-2010 | Isfahan | Snow-ball sampling | Prison | FSW | RPR | 91 | 0.00 |
| Iran | Navadeh, 2012^233^ | 2010 | Kerman | RDS | Community | FSW | VDRL | 139 | 5.76 |
| Lebanon | Assi, 2019^234^ | 2015-2018 | Beirut | Convenience | STD Clinic | MSM | VDRL | 1,429 | 3.01 |
| South Sudan | MOPH, 2020^229^ | 2019 | Wau | RDS | Community | FSW | NR | 679 | 3.53 |
| South Sudan | MOPH, 2020^230^ | 2019 | Yambio | RDS | Community | FSW | NR | 605 | 3.97 |
| South Sudan | MOPH, 2016^228^ | 2015-2016 | Juba | RDS | Community | FSW | RPR | 835 | 12.69 |
| Sudan | Hakim, 2020^235^ | 2015-2016 | Juba | Snow-ball sampling | Sex-work site | FSW | RPR | 838 | 7.30 |
| Yemen | Štulhofer, 2008^236^ | 2008 | Aden | RDS | Hospital | FSW | VDRL | 244 | 4.92 |
| Lifetime Syphilis Infection | |  |  |  |  |  |  |  |  |
| Afghanistan | NAP, 2012^194^ | 2012 | Herat | RDS | Community | FSW | ICT/ ICS | 344 | 0.87 |
| Afghanistan | NAP, 2012 ^194^ | 2012 | Kabul | RDS | Community | FSW | ICT/ ICS | 333 | 0.00 |
| Afghanistan | NAP, 2012 ^194^ | 2012 | Kabul | RDS | Community | MSM | ICT/ ICS | 207 | 10.20 |
| Afghanistan | NAP, 2012 ^194^ | 2012 | Mazar | RDS | Community | FSW | ICT/ ICS | 355 | 1.97 |
| Afghanistan | NAP, 2010 ^194^ | 2009 | Kabul | RDS | Community | FSW | POCT, unspecified | 368 | 5.43 |
| Afghanistan | Todd, 2008^237^ | 2008 | Several Cities | Unclear | NR | Male & FSW | ELISA | 180 | 0.00 |
| Afghanistan | Todd, 2010^238^ | 2006-2008 | Jalalabad, Kabul, Mazar-I-Sharif | Targeted | Sex-work site | FSW | CIA & TPPA | 520 | 0.00 |
| Djibouti | MOPH, 2014^201^ | 2014 | Several areas | Convenience | Community | FSW | TPHA | 361 | 4.99 |
| Iran | Izadi, 2023^239^ | 2019-2020 | Several Cities | RDS | NR | FSW | ICT/ ICS | 1,275 | 0.00 |
| Iran | Khezri, 2020^240^ | 2015 | NR | Convenience | Harm reduction facility | FSW | EIA | 1,290 | 0.47 |
| Lebanon | NAP, 2019^241^ | 2018 | 3 Cities | Time-location sampling | Mix | MSW & FSW | POCT, unspecified | 171 | 1.17 |
| Lebanon | NAP, 2019^241^ | 2018 | 3 Cities | Time-location sampling | Mix | MSW & FSW | POCT, unspecified | 175 | 0.00 |
| Lebanon | NAP, 2019^241^ | 2018 | 3 Cities | Time-location sampling | Mix | MSM | POCT, unspecified | 224 | 3.57 |
| Lebanon | NAP, 2019^241^ | 2018 | 3 Cities | Time-location sampling | Mix | MSM | POCT, unspecified | 245 | 0.41 |
| Pakistan | Bibi, 2010^242^ | 2003 | Hyderabad | Convenience | Sex-work site | FSW | TPHA | 50 | 44.00 |
| Pakistan | Javed, 2023^243^ | 2019-2021 | Punjab | Convenience | Community | Transgender | ELISA | 1,562 | 38.41 |
| Pakistan | Raza, 2015^244^ | 2014 | Rawalpindi | NR | Hospital | Transgender | ICT/ ICS | NR | 76.90 |
| Pakistan | Rehan, 2009^203^ | 2004 | Karachi | Snow-ball | NR | FSW | ELISA | 421 | 3.56 |
| Pakistan | Rehan, 2009^203^ | 2004 | Lahore | Systematic random sampling | Red light district | FSW | ELISA | 387 | 16.02 |
| Pakistan | Rehan, 2009^203^ | 2004 | Karachi | Snow-ball | NR | MSW | ELISA | 399 | 36.09 |
| Pakistan | Rehan, 2009^203^ | 2004 | Lahore | RDS | Community | MSW | ELISA | 387 | 12.92 |
| Pakistan | Rehan, 2009^203^ | 2004 | Karachi | Random cluster sampling | Listing of Gurus (mentors) | Eunuchs | ELISA | 196 | 60.20 |
| Pakistan | Rehan, 2009^203^ | 2004 | Lahore | Random cluster sampling | Listing of Gurus (mentors) | Eunuchs | ELISA | 192 | 35.94 |
| Somalia | Burans, 1990^245^ | NR | Mogadishu | Convenience | STD Clinic | FSW | MHA-TP | 89 | 28.10 |
| Somalia | Jama Ahmed, 1991^226^ | 1988-1989 | Mogadishu | Targeted | ANC clinic | FSW | TPHA | 155 | 69.03 |
| Somalia | Jama, 1987^117^ | 1985-1986 | Mogadishu | Convenience | Military camp | FSW | TPHA | 85 | 12.94 |
| Somalia | Kriitmaa, 2010^246^ | 2008 | Hargeisa | RDS | Community | FSW | CMIA | 219 | 3.65 |
| Somalia | IOM & WHO, 2017^182^ | 2017 | Bossaso | RDS | Community | FSW | ICT/ ICS | 286 | 1.75 |
| Somalia | IOM & WHO, 2017^183^ | 2017 | Hargeisa | RDS | Community | FSW | ICT/ ICS | 287 | 4.88 |
| Somalia | IOM & WHO, 2008^247^ | 2008 | Hargeisa | RDS | Community | FSW | ICT/ ICS | 237 | 3.38 |
| Sudan | Elhadi, 2013^248^; NAP, 2012^249^ | 2011-2012 | Eastern Sudan,  Site 1 | RDS | NR | FSW | ICT/ ICS | 293 | 8.90 |
| Sudan | Elhadi, 2013^248^; NAP, 2012^249^ | 2011-2012 | Western Sudan,  Site 10 | RDS | NR | FSW | ICT/ ICS | 284 | 1.80 |
| Sudan | Elhadi, 2013^248^; NAP, 2012^249^ | 2011-2012 | Western Sudan,  Site 11 | RDS | NR | FSW | ICT/ ICS | 303 | 5.20 |
| Sudan | Elhadi, 2013^248^; NAP, 2012^249^ | 2011-2012 | Northern Sudan,  Site 12 | RDS | NR | FSW | ICT/ ICS | 305 | 1.50 |
| Sudan | Elhadi, 2013^248^; NAP, 2012^249^ | 2011-2012 | Northern Sudan,  Site 13 | RDS | NR | FSW | ICT/ ICS | 291 | 1.90 |
| Sudan | Elhadi, 2013^248^; NAP, 2012^249^ | 2011-2012 | Northern Sudan,  Site 14 | RDS | NR | FSW | ICT/ ICS | 287 | 1.70 |
| Sudan | Elhadi, 2013^248^; NAP, 2012^249^ | 2011-2012 | Eastern Sudan,  Site 2 | RDS | NR | FSW | ICT/ ICS | 288 | 4.30 |
| Sudan | Elhadi, 2013^248^; NAP, 2012^249^ | 2011-2012 | South-Eastern Sudan, Site 3 | RDS | NR | FSW | ICT/ ICS | 282 | 3.40 |
| Sudan | Elhadi, 2013^248^; NAP, 2012^249^ | 2011-2012 | South-Eastern Sudan, Site 4 | RDS | NR | FSW | ICT/ ICS | 296 | 5.40 |
| Sudan | Elhadi, 2013^248^; NAP, 2012^249^ | 2011-2012 | South-Eastern Sudan, Site 5 | RDS | NR | FSW | ICT/ ICS | 303 | 5.30 |
| Sudan | Elhadi, 2013^248^; NAP, 2012^249^ | 2011-2012 | Southern Sudan,  Site 6 | RDS | NR | FSW | ICT/ ICS | 279 | 3.40 |
| Sudan | Elhadi, 2013^248^; NAP, 2012^249^ | 2011-2012 | Southern Sudan,  Site 7 | RDS | NR | FSW | ICT/ ICS | 288 | 4.20 |
| Sudan | Elhadi, 2013^248^; NAP, 2012^249^ | 2011-2012 | Southern Sudan,  Site 8 | RDS | NR | FSW | ICT/ ICS | 296 | 4.10 |
| Sudan | Elhadi, 2013^248^; NAP, 2012^249^ | 2011-2012 | Western Sudan,  Site 9 | RDS | NR | FSW | ICT/ ICS | 299 | 1.80 |
| Sudan | NAP, 2012^249^ | 2011-2012 | Blue Nile | RDS | Mix | MSM | ICT/ ICS | 299 | 4.20 |
| Sudan | NAP, 2012^249^ | 2011-2012 | Gadarif | RDS | Mix | MSM | ICT/ ICS | 298 | 0.00 |
| Sudan | NAP, 2012^249^ | 2011-2012 | Gezira | RDS | Mix | MSM | ICT/ ICS | 135 | 9.80 |
| Sudan | NAP, 2012^249^ | 2011-2012 | Kassala | RDS | Mix | MSM | ICT/ ICS | 321 | 6.40 |
| Sudan | NAP, 2012^249^ | 2011-2012 | Khartoum | RDS | Mix | MSM | ICT/ ICS | 292 | 1.40 |
| Sudan | NAP, 2012^249^ | 2011-2012 | North Kordofan | RDS | Mix | MSM | ICT/ ICS | 304 | 1.10 |
| Sudan | NAP, 2012^249^ | 2011-2012 | Northen | RDS | Mix | MSM | ICT/ ICS | 305 | 0.90 |
| Sudan | NAP, 2012^249^ | 2011-2012 | Red Sea | RDS | Mix | MSM | ICT/ ICS | 300 | 4.20 |
| Sudan | NAP, 2012^249^ | 2011-2012 | River Nile | RDS | Mix | MSM | ICT/ ICS | 300 | 0.00 |
| Sudan | NAP, 2012^249^ | 2011-2012 | Sinnar | RDS | Mix | MSM | ICT/ ICS | 312 | 2.60 |
| Sudan | NAP, 2012^249^ | 2011-2012 | South Darfur | RDS | Mix | MSM | ICT/ ICS | 172 | 2.10 |
| Sudan | NAP, 2012^249^ | 2011-2012 | White Nile | RDS | Mix | MSM | ICT/ ICS | 307 | 0.80 |
| Tunisia | Ayachi, 1997^122^ | 1992-1994 | NR | Convenience | NR | FSW | TPHA | 79 | 36.71 |
| Tunisia | Znazen, 2010^250^ | 2007 | Tunis, Sousse, Gabes | Convenience | Primary health care | FSW | TPHA | 183 | 2.73 |
| Unclear Infection Type | |  |  |  |  |  |  |  |  |
| Iran | Afsar, 2013^251^ | 2010 | Shiraz | RDS | NR | FSW | NR | 278 | 0.00 |
| Iran | Mirzazadeh, 2020^252^ | 2015 | NR | Convenience | Harm reduction facility | FSW | NR | 1,337 | 0.40 |
| Iran | Moayedi-Nia, 2016^253^ | 2012-2013 | Tehran | RDS | Community | FSW | NR | 161 | 0.00 |
| Morocco | Johnston, 2013^254^ | 2011-2012 | Agadir | RDS | Community | FSW | NR | 372 | 21.40 |
| Morocco | Johnston, 2013^254^ | 2011-2012 | Fes | RDS | Community | FSW | NR | 359 | 18.80 |
| Morocco | Johnston, 2013^254^ | 2011-2012 | Rabat | RDS | Community | FSW | NR | 392 | 13.90 |
| Morocco | Johnston, 2013^254^ | 2011-2012 | Tangier | RDS | Community | FSW | NR | 324 | 13.30 |
| Morocco | Johnston, 2018^255^ | 2016 | Agadir | RDS | NR | FSW | NR | 256 | 21.20 |
| Morocco | Johnston, 2018^255^ | 2016 | Casablanca | RDS | NR | FSW | NR | 276 | 15.70 |
| Morocco | Johnston, 2018^255^ | 2016 | Fes | RDS | NR | FSW | NR | 253 | 21.70 |
| Morocco | Johnston, 2018^255^ | 2016 | Marrakesh | RDS | NR | FSW | NR | 254 | 22.90 |
| Morocco | Johnston, 2018^255^ | 2016 | Rabat | RDS | NR | FSW | NR | 250 | 4.70 |
| Morocco | Johnston, 2018^255^ | 2016 | Tangier | RDS | NR | FSW | NR | 261 | 24.80 |
| Morocco | WHO, 2011^135^ | 2010 | Agadir | RDS | Community | MSM | NR | 323 | 2.80 |
| Morocco | WHO, 2011^135^ | 2010 | Marrakesh | RDS | Community | MSM | NR | 346 | 10.80 |
| Morocco | WHO, 2011^135^ | 2007 | NR | Unclear | Community | FSW | NR | NR | 14.00 |
| Pakistan | WHO, 2011^135^ | 2008 | NR | Unclear | Community | FSW | NR | NR | 2.80 |
| Pakistan | WHO, 2011^135^ | 2008 | NR | Unclear | Community | MSW | NR | NR | 9.60 |
| Pakistan | Khan, 2006^256^ | NR | NR | NR | NR | MSW | NR | 400 | 23.00 |
| Pakistan | Khan, 2006^256^ | NR | NR | NR | NR | Transgender | NR | 200 | 62.00 |
| Pakistan | Khan, 2008^257^ | NR | NR | Convenience | Private clinic | Transgender | NR | 409 | 47.43 |
| Pakistan | Narjis, 2011^258^ | NR | NR | RDS | Community | FSW | NR | NR | 2.00 |
| Pakistan | Osama, 2017^259^ | NR | Several Cities | RDS | NGO clinic | Male | NR | 2,531 | 3.26 |
| South Sudan | Solangon, 2018^260^ | NR | six sites | Convenience | Community | MSM | NR | 152 | 3.30 |
| Tunisia | NAP, 2021^261^ | 2021 | Tunis, Sousse, Sfax | RDS | Community | FSW | NR | 780 | 0.26 |
| Tunisia | NAP, 2021^261^ | 2021 | Tunis, Sousse, Sfax | RDS | Community | MSM | NR | 815 | 3.07 |
| Tunisia | WHO, 2011^135^ | 2008 | NR | Unclear | Community | FSW | NR | NR | 2.70 |
| Yemen | WHO, 2011^135^ | 2010 | NR | RDS | Community | FSW | NR | NR | 0.00 |

^*^ The table is sorted by country then by year(s) of data collection.

**^✝^** Indicates a positive reaction on the assay described.

ANC: Antenatal care, CIA: Chemiluminescence immunoassay, CMIA: Chemiluminescent microparticle immunoassay, EIA: Enzyme immunoassay, ELISA: Enzyme-linked immunosorbent assay, FSW: Female sex workers, FTA-ABS: Fluorescent treponemal antibody absorption assay, ICS: Immunochromatographic strip, ICT: Immunochromatographic test, IOM: International Organization for Migration, KSA: Kingdom of Saudi Arabia, MHA-TP: Microhemagglutination assay for *T. pallidum*, MOPH: Ministry of Public Health, MSM: Men who have sex with men, MSW: Male sex workers, NAP: National Aids Control Program, NGO: Non-governmental organization, NR: Not reported, POCT: Point of care test, RDS: Respondent driven sampling, RPR: Rapid plasma reagin, STD: Sexually transmitted disease, TPHA: *T. pallidum* hemagglutination assay, TPPA: *T. pallidum* particle agglutination, VDRL: Venereal disease research laboratory, WHO: World Health Organization.

# Table S8. Syphilis studies among STI clinic attendees in the Middle East and North Africa, stratified by infection type.^*^

| **Country** | **Citation** | **Collection year** | **City** | **Sampling** | **Setting** | **Population** | **Sex** | **Assay^✝^** | **Tested (N)** | **Prevalence**  **(%)** |
| --- | --- | --- | --- | --- | --- | --- | --- | --- | --- | --- |
| Probable Current Syphilis Infection | |  |  |  |  |  |  |  |  |  |
| Iran | Ghanaat, 2003^262^ | 1998-2000 | Mashad | Convenience | Hospital | STI clinic attendees | Mix | VDRL & FTA-ABS | 1,500 | 4.67 |
| Jordan | Mahafzah, 2008^139^ | 2003-2005 | NR | Convenience | Family planning clinic | Symptomatic patients | Female | RPR & TPHA | 1089 | 0.00 |
| KSA | Madani, 2006^263^ | 1995-1999 | Riyadh | Convenience | Mix | STI clinic attendees | Mix | (RPR or VDRL) & (FTA-ABS or TPHA) | 39,049 | 8.67 |
| KSA | Hossain, 1986^12^ | NR | NR | Convenience | NR | Symptomatic patients | Mix | VDRL & TPHA & FTA-ABS | 1,034 | 6.48 |
| KSA | Pareek, 1981^264^ | 1978-1979 | Riyadh | Convenience | Dermato-venereological clinic | STI clinic attendees | Male | VDRL & TPHA & FTA-ABS | 716 | 5.45 |
| KSA | Chowdhury, 1982^171^ | 1978-1980 | Riyadh | Unclear | Dermato-venereological clinic | STI clinic attendees | Mix | VDRL & TPHA & FTA-ABS | 688 | 10.47 |
| KSA | Chowdhury, 1982^171^ | 1978-1980 | Riyadh | Unclear | ENT clinic | STI clinic attendees | Mix | VDRL & TPHA &  FTA-ABS | 172 | 6.98 |
| KSA | Chowdhury, 1982^171^ | 1978-1980 | Riyadh | Unclear | Internal medicine clinic | STI clinic attendees | Mix | VDRL & TPHA &  FTA-ABS | 345 | 4.06 |
| KSA | Chowdhury, 1982^171^ | 1978-1980 | Riyadh | Unclear | Private clinic | STI clinic attendees | Mix | VDRL & TPHA &  FTA-ABS | 68 | 4.41 |
| Morocco | Ryan, 1998^265^ | NR | Several Cities | Convenience | Mix | STI clinic attendees | Female | VDRL & TPHA | 1,238 | 2.75 |
| Pakistan | Maan, 2011^266^ | 2006-2009 | Faisalabad | Multi-stage sampling | Outpatient clinic | STI clinic attendees | Mix | (RPR or VDRL) & TPHA | 1,532 | 29.50 |
| Somalia | Watts, 1994^184^ | 1990-1990 | Several Cities | Non-probability based | Mix | STI clinic attendees | Mix | RPR & FTA-ABS | 80 | 3.75 |
| Somalia | Ismail, 1990^267^ | 1986 | Mogadishu | Convenience | Dermato-venereological clinic | STI clinic attendees | Male | VDRL & TPHA | 101 | 4.95 |
| Somalia | Ismail, 1990^267^ | 1986 | Mogadishu | Convenience | Hospital | Symptomatic patients | Male | VDRL & TPHA | 103 | 1.94 |
| Sudan | Omer, 1982^13^ | 1978 | Khartoum | Convenience | Blood Bank | STI clinic attendees | Male | VDRL & FTA-ABS | 199 | 0.50 |
| Sudan | Omer, 1982^268^ | 1976-1978 | Khartoum | Convenience | STD Clinic | STI clinic attendees | Male | VDRL & FTA-ABS | 77 | 1.30 |
| Sudan | Omer, 1985^269^ | NR | Khartoum | Convenience | GYN clinic | Patients with vaginal discharge | Female | VDRL & FTA-ABS | 822 | 0.00 |
| Sudan | Taha, 1979^270^ | 1976-1978 | NR | Convenience | Venereal Diseases center | STI clinic attendees | Mix | VDRL & FTA-ABS | 148 | 0.68 |
| Possible Current Infection, Unspecified | | |  |  |  |  |  |  |  |  |
| Bahrain | Shareef, 2021^271^ | 2012-2017 | NR | Convenience | NR | STI clinic attendees | Mix | VDRL | 137 | 4.38 |
| Iraq | Kareem, 2020^272^ | 2019-2020 | Thi-Qar | Convenience | NR | Patients with vaginal discharge | Female | VDRL | 110 | 3.60 |
| Morocco | Heikel, 1999^273^ | 1992-1996 | Casablanca | Convenience | STD Clinic | STI clinic attendees | Mix | VDRL | 2,264 | 8.61 |
| Lifetime Syphilis Infection | |  |  |  |  |  |  |  |  |  |
| Iran | Alemi, 1976^274^ | 1971 | Tehran | Convenience | Venereal Diseases center | Symptomatic patients | Mix | POCT, unspecified | 64,880 | 3.65 |
| Iran | Alemi, 1976^274^ | 1970 | Tehran | Convenience | Venereal Diseases center | Symptomatic patients | Mix | POCT, unspecified | 65,954 | 5.69 |
| Iran | Alemi, 1976^274^ | 1969 | Tehran | Convenience | Venereal Diseases center | Symptomatic patients | Mix | POCT, unspecified | 71,724 | 4.68 |
| Iran | Alemi, 1976^274^ | 1968 | Tehran | Convenience | Venereal Diseases center | Symptomatic patients | Mix | POCT, unspecified | 73,010 | 7.28 |
| Iran | Alemi, 1976^274^ | 1967 | Tehran | Convenience | Venereal Diseases center | Symptomatic patients | Mix | POCT, unspecified | 79,497 | 1.02 |
| Iran | Alemi, 1976^274^ | 1966 | Tehran | Convenience | Venereal Diseases center | Symptomatic patients | Mix | POCT, unspecified | 79,161 | 0.78 |
| Iran | Alemi, 1976^274^ | 1965 | Tehran | Convenience | Venereal Diseases center | Symptomatic patients | Mix | POCT, unspecified | 78,732 | 2.43 |
| KSA | Hossain, 1986^12^ | NR | NR | Convenience | NR | People with Venereal Disease | Mix | TPHA | 1,034 | 8.70 |
| Morocco | Heikel, 1999^273^ | 1992-1996 | Casablanca | Convenience | STD Clinic | STI clinic attendees | Mix | TPHA | 2,264 | 13.16 |
| Somalia | Burans, 1990^245^ | NR | Mogadishu | Convenience | STD Clinic | STI clinic attendees | Male | MHA-TP | 45 | 4.40 |
| Somalia | Ismail, 1990^267^ | 1986 | Mogadishu | Convenience | Dermato-venereological clinic | STI clinic attendees | Male | TPHA | 101 | 9.90 |
| Somalia | Ismail, 1990^267^ | 1986 | Mogadishu | Convenience | Hospital | General population | Male | TPHA | 103 | 2.91 |

| **Country** | **Citation** | **Collection year** | **City** | **Sampling** | **Setting** | **Population** | **Sex** | **Assay^✝^** | **Tested (N)** | **Prevalence**  **(%)** |
| --- | --- | --- | --- | --- | --- | --- | --- | --- | --- | --- |
| Unclear Infection Type | |  |  |  |  |  |  |  |  |  |
| Kuwait | Al-Mutairi, 2013^275^ | 2012 | NR | NR | Hospital | STI clinic attendees | Mix | NR | 1,298 | 0.62 |
| Morocco | Toudou-Daouda, 2019^276^ | 2008-2016 | Fez | Convenience | Hospital | Limbic encephalitis patients | Mix | NR | 31 | 16.13 |
| Morocco | Zouhair, 2002^277^ | 1991-2002 | Casablanca | Convenience | Hospital | Women with vulvar dermatosis | Female | NR | 785 | 0.64 |
| Pakistan | Khan, 1995^278^ | 1990-1995 | NR | Convenience | Mix | STI clinic attendees | Mix | NR | 185 | 4.32 |
| Pakistan | Rehan, 2003^279^ | 1999 | NR | Convenience | Mix | STI clinic attendees | Male | NR | 465 | 31.61 |
| Pakistan | Sami, 2005^280^ | 2004 | Quetta | Convenience | GYN clinic | STI clinic attendees | Female | NR | 500 | 0.00 |

^*^ The table is sorted by country then by year(s) of data collection.

**^✝^** Indicates a positive reaction on the assay described.

FTA-ABS: Fluorescent treponemal antibody absorption assay, GYN: Gynecology, KSA: Kingdom of Saudi Arabia, MHA-TP: Microhemagglutination assay for *T. pallidum*, NR: Not reported, POCT: Point of care test, RPR: Rapid plasma reagin, STD: Sexually transmitted disease, STI: Sexually transmitted infection, TPHA: *T. pallidum* hemagglutination assay, VDRL: Venereal disease research laboratory.

**Table S9.** Syphilis studies among special clinical populations in the Middle East and North Africa, stratified by infection type.^*^

| **Country** | **Citation** | **Collection year** | **City** | **Sampling** | **Setting** | **Population** | **Sex** | **Assay^✝^** | **Tested (N)** | **Prevalence**  **(%)** |
| --- | --- | --- | --- | --- | --- | --- | --- | --- | --- | --- |
| Probable Current Syphilis Infection | |  |  |  |  |  |  |  |  |  |
| KSA | Alsuhaibani, 2015^281^ | 1996-2014 | NR | Convenience | Hospital | Stem cell transplant patients | Mix | VDRL&TPPA | 263 | 0.00 |
| KSA | Saxena, 2002^282^ | 1996-2000 | NR | Convenience | Hospital | End stage renal disease | Mix | RPR&MHA-TP | 187 | 6.95 |
| Morocco | Doufik, 2022^283^ | NR | Fez | Unclear | NR | Patients with schizophrenia | Mix | VDRL & TPHA | 444 | 3.60 |
| Somalia | Watts, 1994^184^ | 1990-1990 | Several Cities | Non-probability based | Mix | Tuberculosis patients | Mix | RPR & FTA-ABS | 43 | 0.00 |
| Possible Current Infection, Unspecified | | |  |  |  |  |  |  |  |  |
| Egypt | Ruge, 1955^284^ | NR | NR | Convenience | Mix | Leprosy patients | Mix | VDRL & Wasserman & Meinicke | 820 | 3.78 |
| Egypt | Ruge, 1955^284^ | NR | NR | Convenience | Mix | Tuberculosis patients | Mix | VDRL & Wasserman & Meinicke | 720 | 5.14 |
| Sudan | Sokrab, 2002^285^ | NR | Khartoum | Convenience | Hospital | Patients with acute stroke | Mix | VDRL | 96 | 4.17 |
| Lifetime Syphilis Infection | |  |  |  |  |  |  |  |  |  |
| Iraq | Hasan, 2023^286^ | 2022-2023 | Kirkuk | Convenience | Private Laboratory | Patients with ocular diseases | Mix | ELISA | 146 | 22.6 |
| KSA | Elyamany, 2016^49^ | 2006-2015 | Riyadh | Convenience | Hospital | Stem cell transplant patients | Mix | ELISA | 202 | 0.00 |
| Pakistan | Batool, 2022^287^ | 2020-2021 | South Punjab | Convenience | NR | Patients with Thalassemia | Mix | CIA | 1,212 | 1.70 |
| Syria | Ali, 2012^288^ | NR | Several Cities | Convenience | Hospital | Individuals with hemophilia | Mix | ELISA | 375 | 0.27 |
| UAE | Abdo, 2024^289^ | 2021 | Abu Dhabi | Convenience | Outpatient Clinic | Infertile subjects | Mix | ELISA | 299 | 3.00 |
| Unclear Infection Type | |  |  |  |  |  |  |  |  |  |
| Pakistan | Waheed, 2017^290^ | 2016 | Islamabad | Convenience | Blood Bank | Blood donors with transfusion transmissible infections | NR | NR | 2,538 | 0.72 |
| Sudan | El-Hassan, 1972^291^ | 1957-1969 | Khartoum | Convenience | Hospital | Patients with acute stroke | Mix | NR | 528 | 3.79 |

^*^ The table is sorted by country then by year(s) of data collection.

**^✝^** Indicates a positive reaction on the assay described.

CIA: Chemiluminescence immunoassay, ELISA: Enzyme-linked immunosorbent assay, KSA: Kingdom of Saudi Arabia, MHA-TP: Microhemagglutination assay for *T. pallidum*, NR: Not reported, RPR: Rapid plasma regain, TPHA: *T. pallidum* hemagglutination assay, TPPA: *T. pallidum* particle agglutination, VDRL: Venereal disease research laboratory.

# Table S10. Syphilis studies among mixed populations in the Middle East and North Africa, stratified by infection type.^*^

| **Country** | **Citation** | **Collection year** | **City** | **Sampling** | **Setting** | **Sex** | **Assay^✝^** | **Tested (N)** | **Prevalence**  **(%)** |
| --- | --- | --- | --- | --- | --- | --- | --- | --- | --- |
| Probable Current Syphilis Infection | |  |  |  |  |  |  |  |  |
| KSA | Wanni, 2021^292^ | 2014-2018 | Eastern Province | Convenience | Tertiary care hospital | Mix | RPR & CIA | 11,832 | 0.46 |
| Somalia | Scott, 1991^293^ | 1989 | Several Cities | Convenience | Mix | Mix | RPR & FTA-ABS | 1,269 | 7.70 |
| Sudan | Mccarthy, 1989^294^ | 1987 | Port Sudan, Suakin | Convenience | Mix | Mix | RPR & FTA-ABS | 536 | 17.35 |
| Possible Current Infection, Unspecified | |  |  |  |  |  |  |  |  |
| Morocco | Mailloux, 1971^295^ | NR | Tangier | Convenience | Mix | NR | VDRL | 31,174 | 16.45 |

^*^ The table is sorted by country then by year(s) of data collection.

**^✝^** Indicates a positive reaction on the assay described.

CIA: Chemiluminescence immunoassay, FTA-ABS: Fluorescent treponemal antibody absorption assay, KSA: Kingdom of Saudi Arabia, NR: Not reported, RPR: Rapid plasma reagin, VDRL: Venereal disease research laboratory.

# Table S11. Syphilis studies in the Middle East and North Africa as extracted from the WHO Global Health Observatory data repository.^296*^

| **Country** | **Midyear data collection** | **Sampling** | **Population** | **Sex** | **Assay^✝^** | **Tested**  **(N)** | **Prevalence**  **(%)** |
| --- | --- | --- | --- | --- | --- | --- | --- |
| Probable Current Syphilis Infection | | |  |  |  |  |  |
| Afghanistan | 2017 | NR | Pregnant women | Female | Any NTT combination & any TT | 1,181 | 0.25 |
| Egypt | 2000 | NR | Tourism workers | Male | RPR & TPHA | 250 | 0.40 |
| Iran | 2022 | NR | Pregnant women | Female | Any NTT combination & any TT | 852,527 | 0.00 |
| Iran | 2021 | NR | Pregnant women | Female | Any NTT combination & any TT | 682,903 | 0.00 |
| Iran | 2020 | NR | Pregnant women | Female | Any NTT combination & any TT | 552,060 | 0.00 |
| Iran | 2019 | NR | Pregnant women | Female | Any NTT combination & any TT | 354,521 | 0.00 |
| Iran | 2018 | NR | Pregnant women | Female | Any NTT combination & any TT | 634,203 | 0.00 |
| Iran | 2017 | NR | Pregnant women | Female | Any NTT combination & any TT | 81,992 | 0.00 |
| Jordan | 2003 | NR | Patients | Female | RPR & TPHA | 213 | 0.00 |
| KSA | 2022 | NR | Pregnant women | Female | Any NTT combination & any TT | 118,606 | 0.01 |
| KSA | 2018 | NR | Pregnant women | Female | Any NTT combination & any TT | 150,935 | 0.00 |
| KSA | 2017 | NR | Pregnant women | Female | Any NTT combination & any TT | 147,264 | 0.00 |
| KSA | 2016 | NR | Pregnant women | Female | Any NTT combination & any TT | 159,143 | 0.00 |
| Morocco | 2012 | NR | FSW | Female | VDRL& TPHA | 1,431 | 17.68 |
| Morocco | 2007 | NR | FSW | Female | VDRL& TPHA | 141 | 13.48 |
| Morocco | 2021 | NR | Pregnant women | Female | Any NTT combination & any TT | 122,019 | 0.39 |
| Morocco | 2020 | NR | Pregnant women | Female | Any NTT combination & any TT | 142,746 | 0.63 |
| Morocco | 2019 | NR | Pregnant women | Female | Any NTT combination & any TT | 214,268 | 0.34 |
| Morocco | 2018 | NR | Pregnant women | Female | Any NTT combination & any TT | 201,419 | 0.88 |
| Oman | 2018 | NR | Pregnant women | Female | Any NTT combination & any TT | 88,124 | 0.02 |
| Oman | 2017 | NR | Pregnant women | Female | Any NTT combination & any TT | 87,707 | 0.03 |
| Oman | 2016 | NR | Pregnant women | Female | Any NTT combination & any TT | 91,014 | 0.01 |
| Oman | 2015 | NR | Pregnant women | Female | Any NTT combination & any TT | 83,002 | 0.01 |
| Oman | 2014 | NR | Pregnant women | Female | Any NTT combination & any TT | 82,855 | 0.03 |
| Oman | 2013 | NR | Pregnant women | Female | Any NTT combination & any TT | 77,934 | 0.01 |
| Somalia | 2017 | NR | Low risk population | Male | RPR & TPHA | 2,296 | 0.48 |
| South Sudan | 2022 | NR | Pregnant women | Female | Any NTT combination & any TT | 128,501 | 5.48 |
| Syria | 2014 | NR | Refugees/IDPs | Mix | RPR & TPHA | 140 | 0.71 |
| Tunisia | 2001 | NR | Low risk population | Female | RPR & TPHA | 477 | 0.84 |
| UAE | 2014 | NR | Pregnant women | Female | Any NTT combination & any TT | 35,411 | 0.03 |
| UAE | 2012 | NR | Pregnant women | Female | Any NTT combination & any TT | 67,198 | 0.03 |
| Possible current Infection, Unspecified | | NR |  |  |  |  |  |
| Afghanistan | 2019 | NR | Pregnant women | Female | Any NTT combination | 293 | 0.34 |
| Afghanistan | 2015 | NR | Pregnant women | Female | Any NTT combination | 1,611 | 0.60 |
| Iran | 2011 | NR | Pregnant women | Female | Any NTT combination | 5,261 | 0.04 |
| KSA | 2014 | NR | Pregnant women | Female | Any NTT combination | 214,739 | 0.00 |
| KSA | 2013 | NR | Pregnant women | Female | Any NTT combination | 243,862 | 0.00 |
| Pakistan | 2013 | NR | Blood Donors | Mix | VDRL or RPR | 1,569,995 | 0.26 |
| Pakistan | 2012 | NR | Blood Donors | Mix | VDRL or RPR | 1,202,278 | 1.16 |
| Pakistan | 2017 | NR | Low risk population | Mix | VDRL or RPR | 48,022 | 0.83 |
| Pakistan | 2016 | NR | Low risk population | Mix | VDRL or RPR | 1,229,795 | 1.38 |
| Pakistan | 2015 | NR | Low risk population | Mix | VDRL or RPR | 697,275 | 1.09 |
| Somalia | 2016 | NR | Pregnant women | Female | Any NTT combination | 31,555 | 1.28 |
| Somalia | 2015 | NR | Pregnant women | Female | Any NTT combination | 8,156 | 5.90 |
| South Sudan | 2021 | NR | Pregnant women | Female | Any NTT combination | 234,934 | 7.19 |
| South Sudan | 2020 | NR | Pregnant women | Female | Any NTT combination | 262,645 | 5.73 |
| South Sudan | 2017 | NR | Pregnant women | Female | Any NTT combination | 7,494 | 7.59 |
| UAE | 2021 | NR | Pregnant women | Female | Any NTT combination | 28,859 | 0.13 |
| UAE | 2020 | NR | Pregnant women | Female | Any NTT combination | 16,086 | 0.13 |
| UAE | 2019 | NR | Pregnant women | Female | Any NTT combination | 21,756 | 0.07 |
| UAE | 2018 | NR | Pregnant women | Female | Any NTT combination | 24,045 | 0.02 |
| UAE | 2017 | NR | Pregnant women | Female | Any NTT combination | 25,233 | 0.06 |
| UAE | 2016 | NR | Pregnant women | Female | Any NTT combination | 28,698 | 0.04 |
| UAE | 2015 | NR | Pregnant women | Female | Any NTT combination | 41,299 | 0.04 |
| Lifetime Syphilis Infection | |  |  |  |  |  |  |
| Afghanistan | 2012 | NR | Blood Donors | Male | TPHA | 74,811 | 0.50 |
| Afghanistan | 2012 | RDS | MSM & Bisexual | Male | POCT, unspecified | 207 | 17.39 |
| Algeria | 2014 | NR | Pregnant women | Female | Any TT combination | 910,800 | 0.20 |
| Egypt | 2012 | NR | Blood Donors | Mix | TPHA | 49,142 | 0.05 |
| Egypt | 2011 | NR | Blood Donors | Mix | TPHA | 52,542 | 0.17 |
| Egypt | 2010 | NR | Blood Donors | Mix | TPHA | 49,036 | 0.25 |
| Egypt | 2009 | NR | Blood Donors | Mix | TPHA | 51,852 | 0.16 |
| Egypt | 2008 | NR | Blood Donors | Mix | TPHA | 49,600 | 0.10 |
| Egypt | 2007 | NR | Blood Donors | Mix | TPHA | 56,590 | 0.06 |
| Morocco | 2017 | NR | Pregnant women | Female | Any TT combination | 214,906 | 1.33 |
| Somalia | 2019 | NR | Pregnant women | Female | Any TT combination | 78,912 | 4.07 |
| Somalia | 2014 | NR | Pregnant women | Female | Any TT combination | 8,179 | 1.50 |
| South Sudan | 2019 | NR | Pregnant women | Female | Any TT combination | 16,033 | 10.90 |
| South Sudan | 2018 | NR | Pregnant women | Female | Any TT combination | 43,852 | 5.76 |
| South Sudan | 2013 | NR | Pregnant women | Female | Any TT combination | 252 | 5.60 |
| Sudan | 2010 | RDS | FSW | Female | POCT, unspecified | 4,094 | 4.40 |
| Unclear Infection Type | |  |  |  |  |  |  |
| Afghanistan | 2020 | NR | FSW | Female | NR | 3,826 | 1.20 |
| Afghanistan | 2018 | NR | FSW | Female | NR | 3,904 | 0.44 |
| Afghanistan | 2017 | NR | FSW | Female | NR | 2,457 | 1.34 |
| Afghanistan | 2012 | NR | FSW | Female | NR | 440 | 5.68 |
| Afghanistan | 2012 | NR | FSW | Female | NR | 1,032 | 0.97 |
| Afghanistan | 2010 | NR | FSW | Female | NR | NR | 8.70 |
| Afghanistan | 2021 | NR | MSM | Male | NR | 809 | 1.48 |
| Afghanistan | 2020 | NR | MSM | Male | NR | 6,893 | 0.52 |
| Afghanistan | 2018 | NR | MSM | Male | NR | 8,799 | 0.76 |
| Afghanistan | 2017 | NR | MSM | Male | NR | 1,039 | 10.80 |
| Afghanistan | 2012 | NR | MSM | Male | NR | 206 | 10.19 |
| Afghanistan | 2012 | NR | MSM | Male | NR | 499 | 18.84 |
| Afghanistan | 2016 | NR | Pregnant women | Female | NR | 928 | 0.32 |
| Algeria | 2018 | NR | FSW | Female | NR | 163 | 9.20 |
| Algeria | 2017 | NR | FSW | Female | NR | 81 | 16.05 |
| Algeria | 2016 | NR | FSW | Female | NR | 183 | 14.20 |
| Algeria | 2016 | NR | FSW | Female | NR | 209 | 12.44 |
| Algeria | 2014 | NR | FSW | Female | NR | 24 | 29.17 |
| Algeria | 2013 | NR | FSW | Female | NR | 27 | 7.41 |
| Algeria | 2018 | NR | MSM | Male | NR | 1,127 | 2.22 |
| Algeria | 2017 | NR | MSM | Male | NR | 69 | 2.90 |
| Algeria | 2016 | NR | MSM | Male | NR | 131 | 36.64 |
| Algeria | 2011 | NR | Pregnant women | Female | NR | 433 | 1.85 |
| Bahrain | 2013 | NR | Blood Donors | Mix | NR | NR | 0.10 |
| Bahrain | 2012 | NR | Blood Donors | Mix | NR | 19,484 | 0.09 |
| Bahrain | 2011 | NR | Blood Donors | Mix | NR | NR | 0.33 |
| Bahrain | 1998 | NR | Blood Donors | Mix | NR | NR | 0.24 |
| Djibouti | 2014 | NR | FSW | Female | NR | 361 | 4.99 |
| Egypt | 2013 | NR | Blood Donors | Mix | NR | 424,301 | 0.09 |
| Egypt | 2012 | NR | Blood Donors | Mix | NR | 423,849 | 0.09 |
| Egypt | 2011 | NR | Blood Donors | Mix | NR | 355,111 | 0.06 |
| Iran | 2013 | NR | Blood Donors | Mix | NR | 2,001,791 | 0.00 |
| Iran | 2012 | NR | Blood Donors | Mix | NR | 2,042,315 | 0.00 |
| Iran | 2011 | NR | Blood Donors | Mix | NR | 1,989,867 | 0.00 |
| Iran | 2020 | NR | FSW | Female | NR | 1,275 | 0.00 |
| Iran | 2010 | RDS | FSW | Female | NR | 291 | 0.00 |
| Iran | 2008 | NR | FSW | Female | NR | NR | 1.60 |
| Jordan | 2008 | NR | FSW | Female | NR | NR | 6.70 |
| KSA | 2015 | NR | Pregnant women | Female | NR | 136,272 | 0.00 |
| Kuwait | 2015 | NR | Blood Donors | Male | NR | 77,700 | 0.20 |
| Kuwait | 2011 | NR | Blood Donors | Mix | NR | 68,898 | 0.17 |
| Kuwait | 2016 | NR | MSM | Male | NR | 26 | 3.85 |
| Morocco | 2019 | RDS | FSW | Female | NR | 1,512 | 12.30 |
| Morocco | 2008 | NR | FSW | Female | NR | NR | 16.90 |
| Morocco | 2020 | RDS | MSM | Male | NR | 843 | 8.30 |
| Morocco | 2017 | RDS | MSM | Male | NR | 1,055 | 8.91 |
| Morocco | 2010 | RDS | MSM | Male | NR | 659 | 8.35 |
| Morocco | 2012 | NR | Pregnant women | Female | NR | 8,500 | 0.87 |
| Morocco | 2010 | NR | Pregnant women | Female | NR | 3,147 | 0.60 |
| Oman | 2012 | NR | Pregnant women | Female | NR | 71,330 | 0.02 |
| Oman | 2011 | NR | Pregnant women | Female | NR | 16,895 | 0.12 |
| Qatar | 2013 | NR | Blood Donors | Mix | NR | 23,857 | 0.45 |
| Somalia | 2011 | NR | Pregnant women | Female | NR | 3,030 | 8.70 |
| South Sudan | 2019 | NR | FSW | Female | NR | 1,284 | 2.02 |
| South Sudan | 2018 | NR | FSW | Female | NR | 408 | 9.07 |
| South Sudan | 2017 | NR | FSW | Female | NR | 1,244 | 14.39 |
| South Sudan | 2012 | NR | Pregnant women | Female | NR | 11,018 | 8.30 |
| South Sudan | 2011 | NR | Pregnant women | Female | NR | 6,175 | 9.90 |
| Sudan | 2015 | NR | FSW | Female | NR | 4,123 | 4.12 |
| Sudan | 2011 | RDS | FSW | Female | NR | 4,242 | 4.38 |
| Sudan | 2021 | NR | MSM | Male | NR | 7,155 | 1.84 |
| Sudan | 2015 | NR | MSM | Male | NR | 4,131 | 1.50 |
| Sudan | 2011 | RDS | MSM | Male | NR | 217 | 2.50 |
| Sudan | 2011 | NR | MSM | Male | NR | 3,361 | 2.50 |
| Syria | 2011 | NR | Low risk population | Mix | NR | NR | 1.31 |
| Tunisia | 2013 | NR | Blood Donors | Mix | NR | 216,907 | 0.08 |
| Tunisia | 2012 | NR | Blood Donors | Mix | NR | NR | 0.10 |
| Tunisia | 2011 | NR | Blood Donors | Mix | NR | NR | 0.19 |
| UAE | 2022 | NR | Pregnant women | Female | NR | 91,191 | 0.00 |
| UAE | 2011 | NR | Pregnant women | Female | NR | 54,552 | 0.05 |
| UAE | 2010 | NR | Pregnant women | Female | NR | 50,895 | 0.00 |
| Yemen | 2012 | NR | Blood Donors | Mix | NR | 17,000 | 0.20 |
| Yemen | 2010 | NR | Pregnant women | Female | NR | 1,422 | 0.35 |

^*^ The table is sorted by country then by population then by mid-year of data collection.

**^✝^** Indicates a positive reaction on the assay described.

FSW: Female sex workers, KSA: Kingdom of Saudi Arabia, MSM: Men who have sex with men, NR: Not reported, NTT: Non-treponemal test, POCT: Point of care test, RDS: Respondent driven sampling, RPR: Rapid plasma reagin, TPHA: *T. pallidum* hemagglutination assay, TT: Treponemal test, UAE: United Arab Emirates, VDRL: Venereal disease research laboratory

# Figure S12. Trend of syphilis prevalence over time, all prevalence measures included in the meta-analytics (n=935).

This graph displays all syphilis prevalence measures included in the meta-analyses and meta-regressions for all population groups combined. The dashed line shows the linear trend in syphilis prevalence data over time.


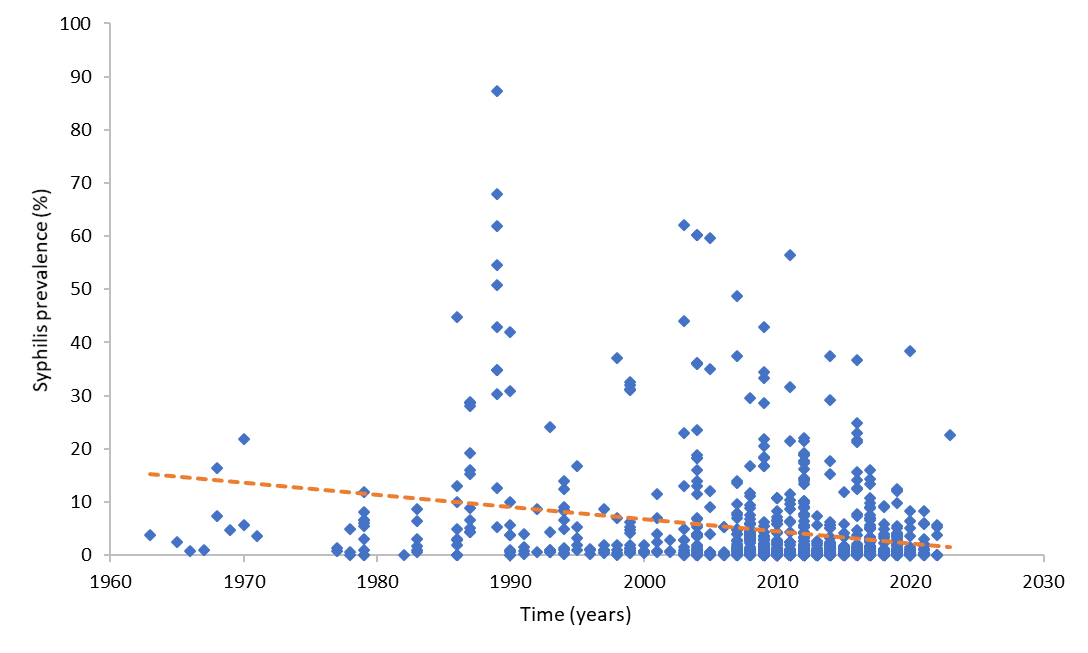


# Table S13. Distribution of syphilis prevalence studies by key characteristics in the Middle East and North Africa (n=643).

|  | **N** | % |
| --- | --- | --- |
| **Subregion/country^*^** |  |  |
| Fertile Crescent | 57 | 8.9 |
| Eastern MENA | 114 | 17.7 |
| Pakistan | 125 | 19.4 |
| Gulf | 90 | 14.0 |
| North Africa | 92 | 14.3 |
| Horn of Africa | 165 | 25.7 |
| **Population Type** |  |  |
| Blood donors | 145 | 22.6 |
| Pregnant women | 117 | 18.2 |
| Other general population groups | 51 | 7.9 |
| Populations at intermediate risk | 88 | 13.7 |
| Populations at high risk | 185 | 28.8 |
| STI clinic attendees | 39 | 6.1 |
| Special clinical populations | 14 | 2.2 |
| Mixed populations | 4 | 0.6 |
| **Sex** |  |  |
| Male | 150 | 23.3 |
| Female | 270 | 42.0 |
| Mix | 200 | 31.1 |
| Not reported | 23 | 3.6 |
| **Year of data collection** |  |  |
| <2000 | 102 | 15.9 |
| 2000-2009 | 153 | 23.8 |
| 2010-2019 | 353 | 54.9 |
| ≥2020 | 35 | 5.4 |
| **Infection type** |  |  |
| Probable current syphilis infection | 181 | 28.1 |
| Possible current infection, unspecified | 90 | 14.0 |
| Lifetime syphilis infection | 218 | 33.9 |
| Unclear infection time | 154 | 24.0 |

^*^ These were ordered according to the hierarchy of prevalence of STIs as commonly observed in previous studies in MENA.^297,298^

MENA: Middle East and North Africa.

# Table S14. Time span of syphilis prevalence studies by population type in the Middle East and North Africa (n=643).

|  | **Mid-year of data collection** | |
| --- | --- | --- |
|  | **Range** | **Median** |
| **Population Type** |  |  |
| Blood donors | 1978-2022 | 2013 |
| Pregnant women | 1983-2022 | 2012 |
| Other general population groups | 1950-2019 | 1999 |
| Populations at intermediate risk | 1978-2019 | 2009 |
| Populations at high risk | 1986-2021 | 2012 |
| STI clinic attendees | 1965-2020 | 1986 |
| Special clinical populations | 1952-2023 | 2007 |
| Mixed populations | 1968-2016 | 1988 |

# Figure S15. Number of studies reporting probable current syphilis infection prevalence measures by year and type of population in the Middle East and North Africa (n=181).


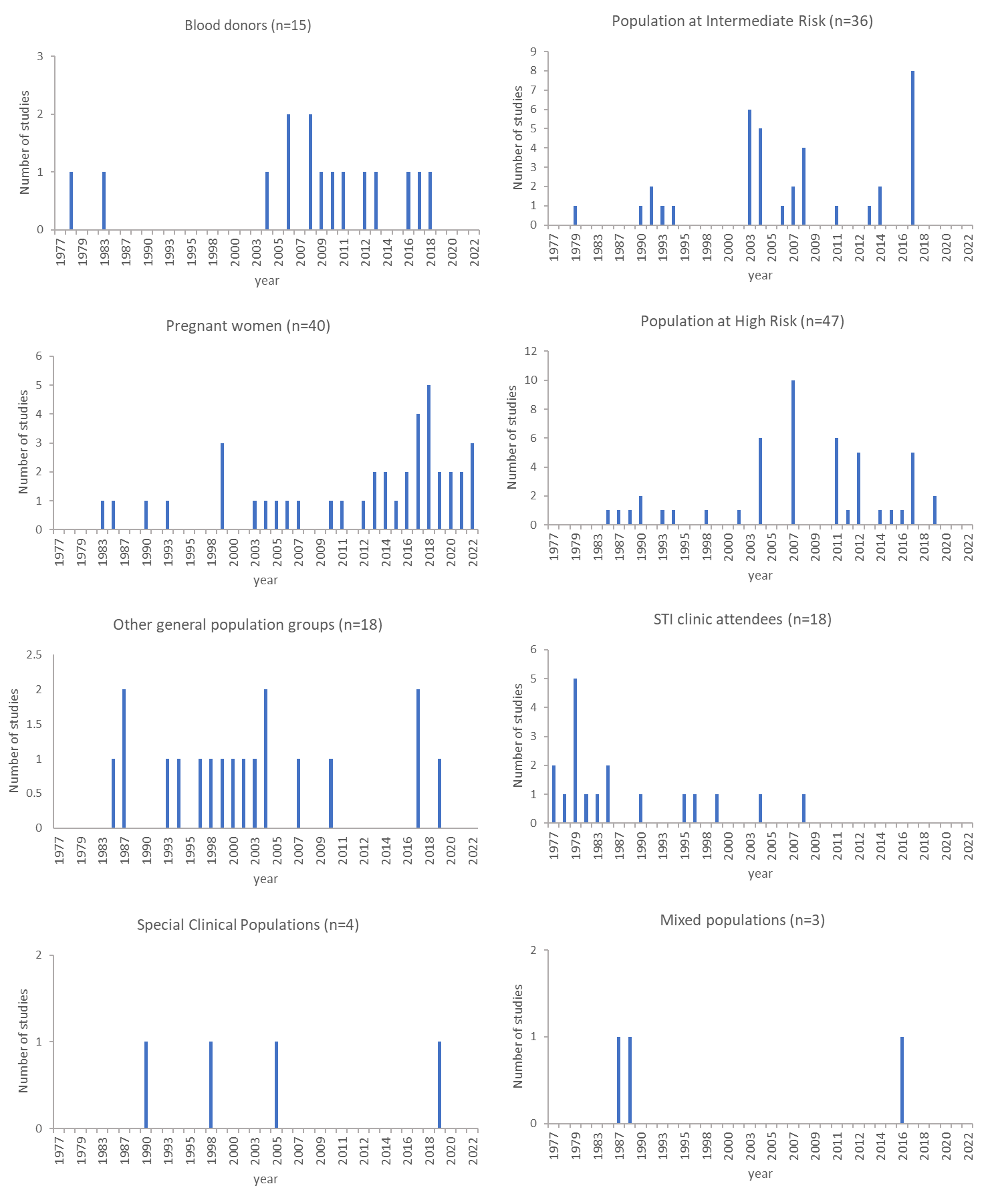


# Table S16. Summary of precision and risk of bias of syphilis prevalence measures in the Middle East and North Africa (n=643).

|  | **Number** | **Percent (%)** |
| --- | --- | --- |
| **Precision**^*^ |  |  |
| High precision | 419 | 65.2 |
| Low precision | 190 | 29.5 |
| Missing | 34 | 5.3 |
| **Risk of bias quality domain**^*^ |  |  |
| **Sampling methodology** |  |  |
| Low risk of bias | 174 | 27.1 |
| High risk of bias | 280 | 43.5 |
| Unclear | 189 | 30.0 |
| **Syphilis ascertainment** |  |  |
| Low risk of bias | 489 | 76.0 |
| Unclear | 154 | 24.5 |
| Total | 643 | 100.0 |
|  |  |  |
|  | **Number** | **Percent (%)** |
| **Low risk of bias** |  |  |
| In at least one quality domain | 517 | 80.4 |
| In both quality domains | 146 | 22.7 |

^*^ Precision and risk of bias were assessed based on the overall sample size (not each stratum subsample size) of the study as reported in the record/publication.

# Table S17. Assessment of publication bias using Doi plots and the LFK index.

| **Population type** | **Outcome measures** | **LFK index** | **Doi plot inspection** | **Interpretation** |
| --- | --- | --- | --- | --- |
|  | **Total n** |  |  |  |
| Blood donors | 281 | -4.86 | Asymmetrical Doi plot | Indicative of publication bias |
| Pregnant women | 190 | 5.27 | Asymmetrical Doi plot | Indicative of publication bias |
| Other general populations | 89 | 4.71 | Asymmetrical Doi plot | Indicative of publication bias |
| Populations at intermediate risk | 110 | 3.72 | Asymmetrical Doi plot | Indicative of publication bias |
| Populations at high risk | 194 | 3.24 | Asymmetrical Doi plot | Indicative of publication bias |
| STI clinic attendees | 52 | 3.16 | Asymmetrical Doi plot | Indicative of publication bias |
| Special clinical populations | 13 | 0.77 | Symmetrical Doi plot | No publication bias |
| Mixed populations | 6 | -3.91 | Asymmetrical Doi plot | Indicative of publication bias |

LFK = Luis Furuya-Kanamori, STI = Sexually transmitted infection.

# Table S18. Results of meta-analyses on studies reporting probable syphilis infection prevalence among people who inject drugs, people living with HIV/AIDS, and incarcerated people in the Middle East and North Africa.

|  | **Prevalence Measures** | **Samples** | | **Reported Prevalence** | **Pooled Mean Prevalence** | **Heterogeneity** |
| --- | --- | --- | --- | --- | --- | --- |
|  | **N** | **Tested** | **Positive** | **Median (%)** | **Estimate (95% CI)** | **I^2^** |
| **PWID** |  |  |  |  |  |  |
| Probable current syphilis infection | 6 | 1,613 | 153 | 7.12 | 8.38 (4.57-13.16) | 90.9% |
| Possible current infection, unspecified | 0 | -- | -- | -- | -- | -- |
| Lifetime syphilis infection | 19 | 4,688 | 307 | 3.50 | 5.46 (3.21-8.24) | 92.8% |
| Unclear infection time | 0 | -- | -- | -- | -- | -- |
| **Incarcerated people** |  |  |  |  |  |  |
| Probable syphilis infection | 3 | 573 | 73 | 9.40 | 12.34 (6.51-19.63) | 82.1% |
| Possible current infection, unspecified | 1 | 163 | 0 | 0.00 | -- | -- |
| Lifetime syphilis infection | 5 | 1,605 | 65 | 0.85 | 3.23 (0.00-11.13) | 96.8% |
| Unclear infection time | 1 | 129 | 8 | 6.20 | -- | -- |
| **PLHIV** |  |  |  |  |  |  |
| Probable syphilis infection | 6 | 3,375 | 471 | 9.61 | 7.32 (1.52-16.18) | 97.4% |
| Possible current infection, unspecified | 3 | 1,335 | 25 | 1.27 | 1.39 (0.00-6.36) | 96.4% |
| Lifetime syphilis infection | 1 | 58 | 2 | 3.45 | -- | -- |
| Unclear infection time | 4 | 1,619 | 26 | 1.64 | 0.99 (0.46-1.65) | 23.9% |

CI: confidence interval; PWID: people who inject drugs; PLHIV: people living with HIV.

# Figure S19. Forest plots presenting outcomes of the pooled mean probable current syphilis prevalence among different populations in the Middle East and North Africa.

## Blood donors


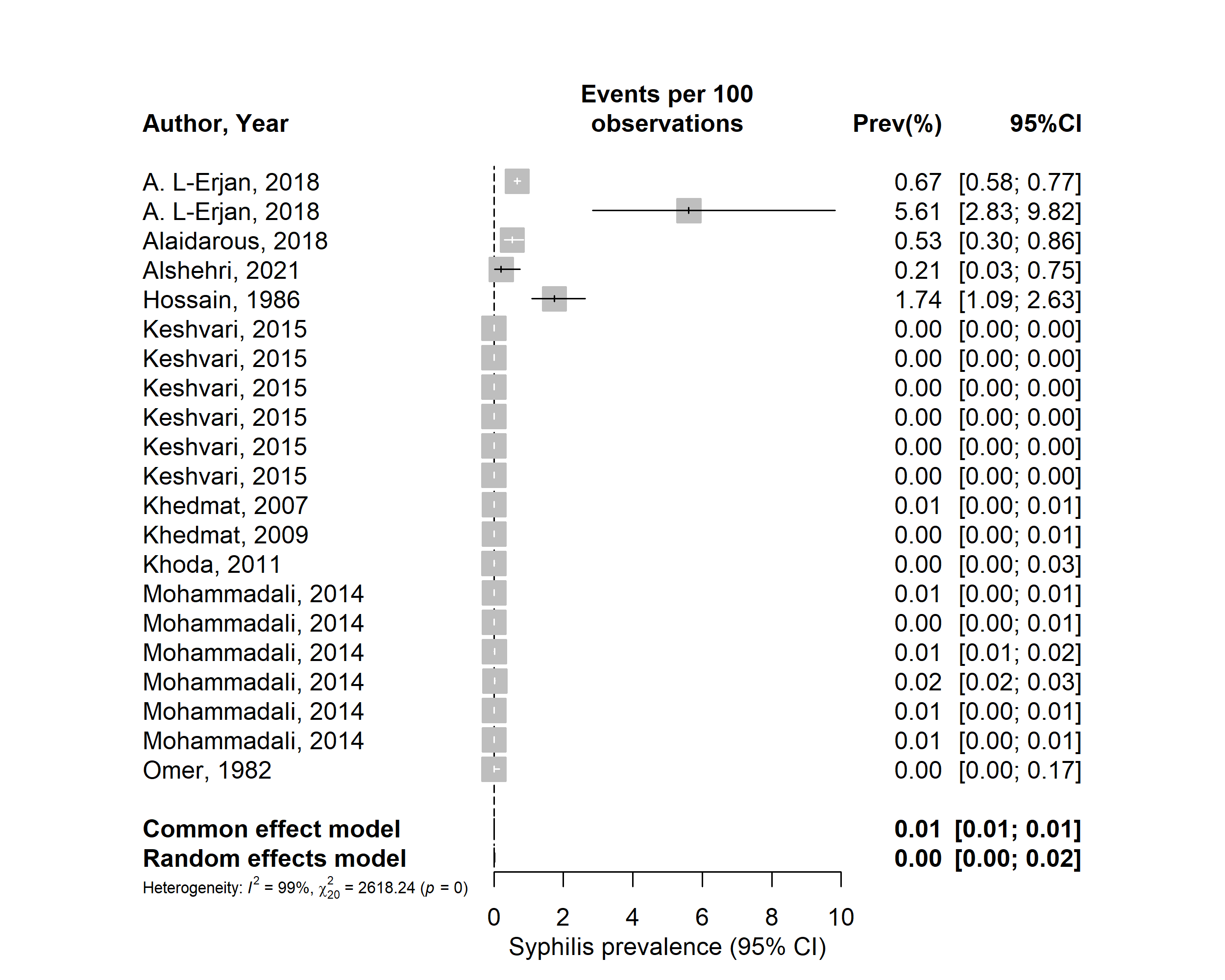


## Pregnant women


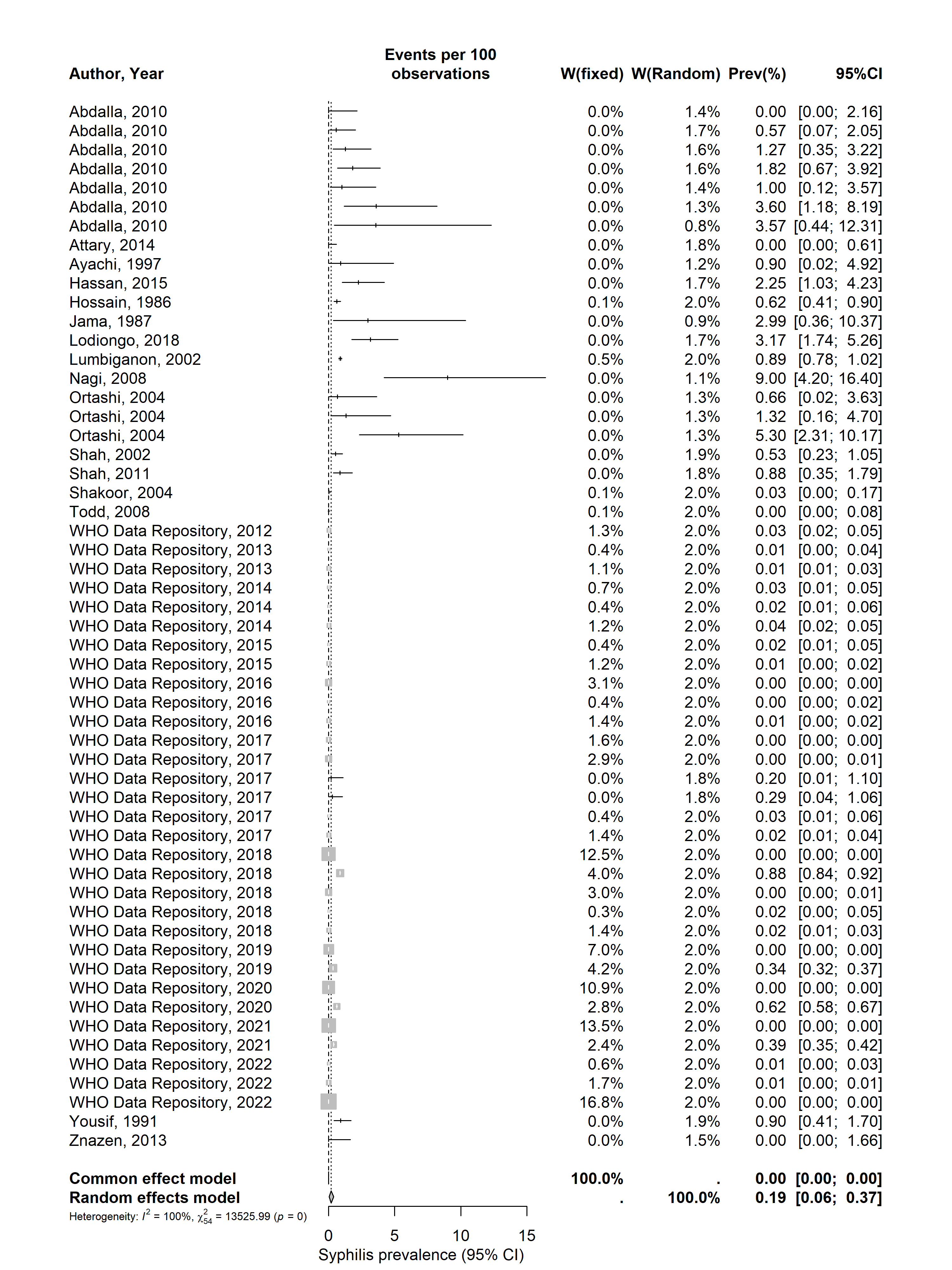


## Other general population groups

**
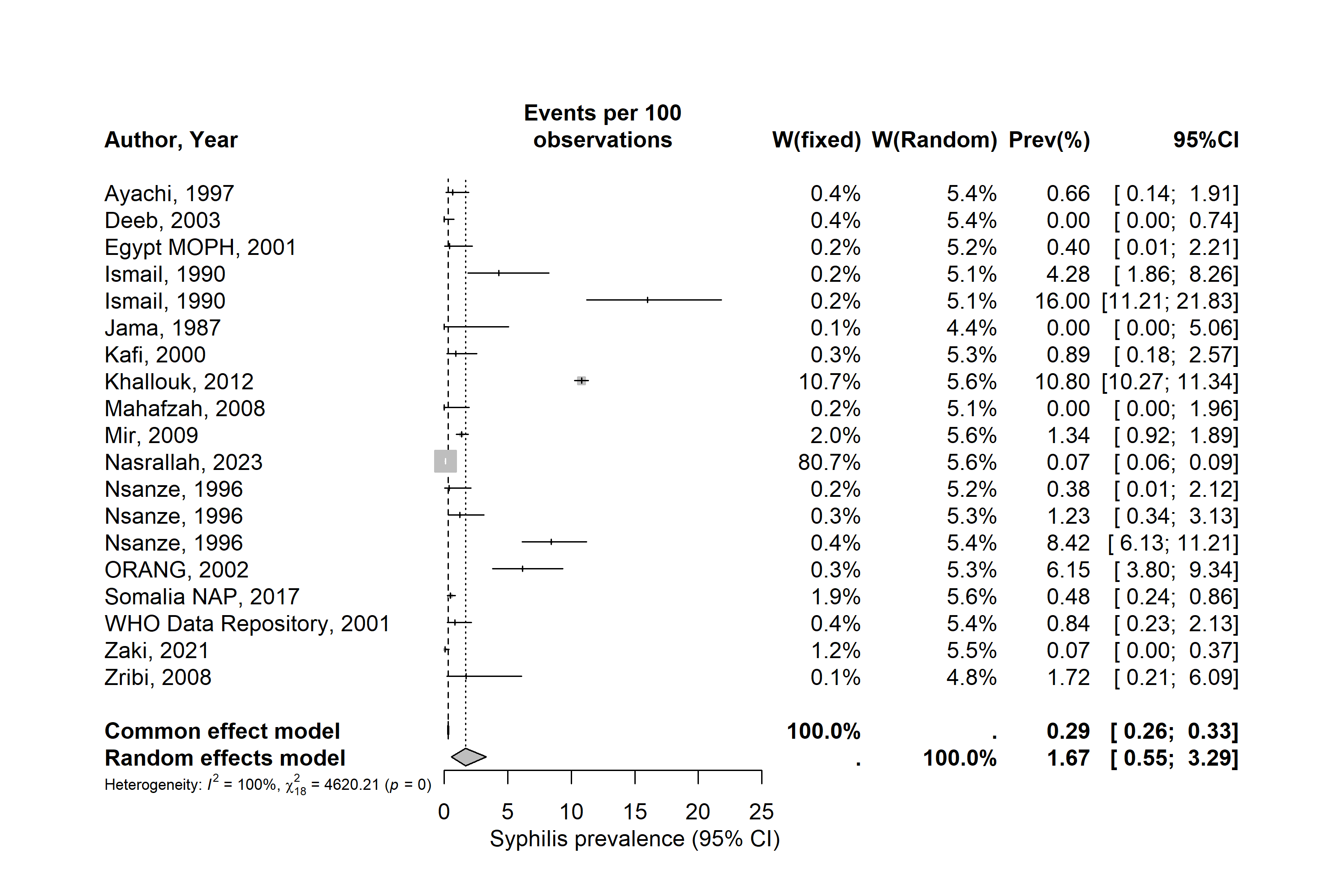
**

## Populations at intermediate risk


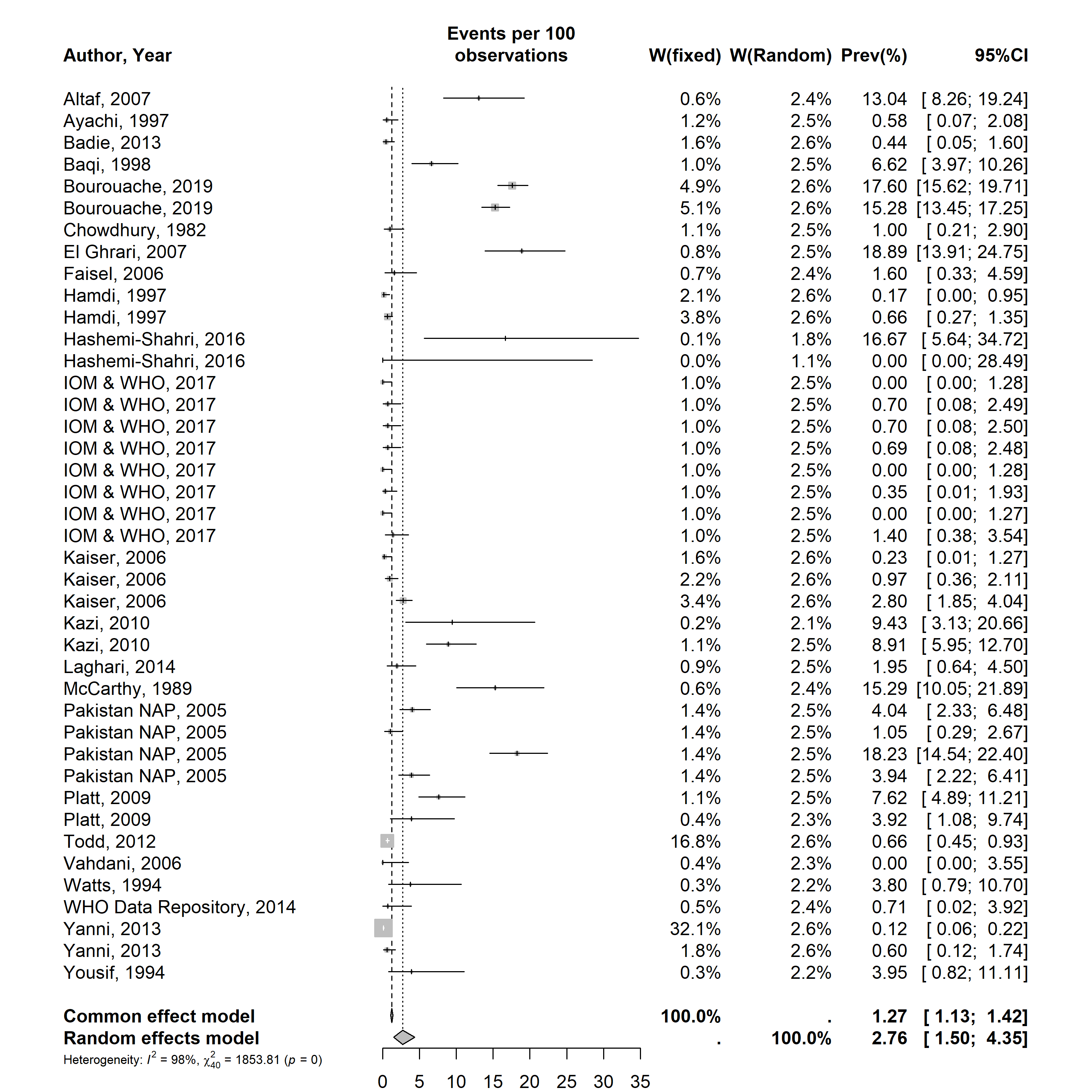


## Populations at high risk (overall)


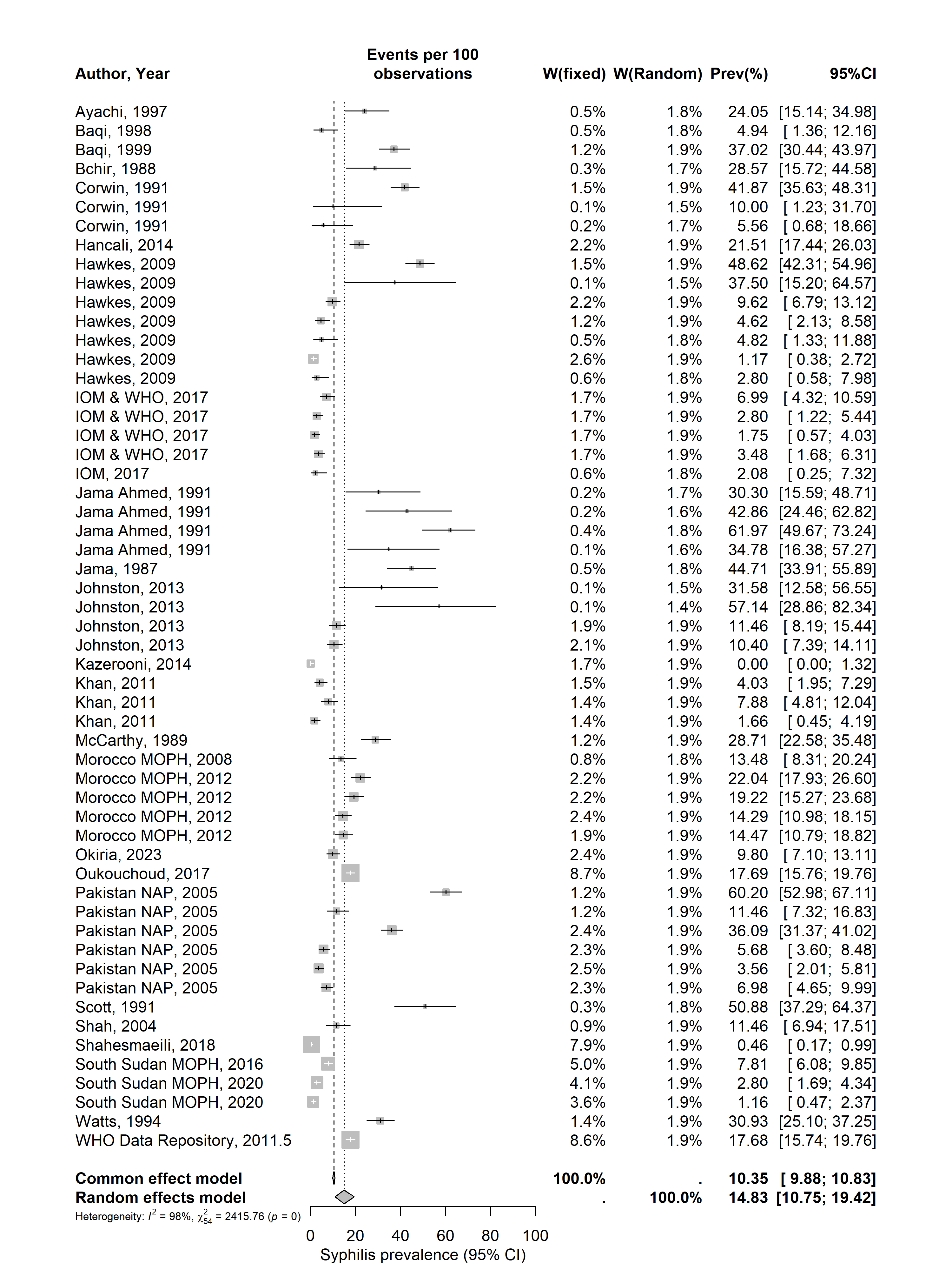


## Populations at high risk (men who have sex with men & transgender people)


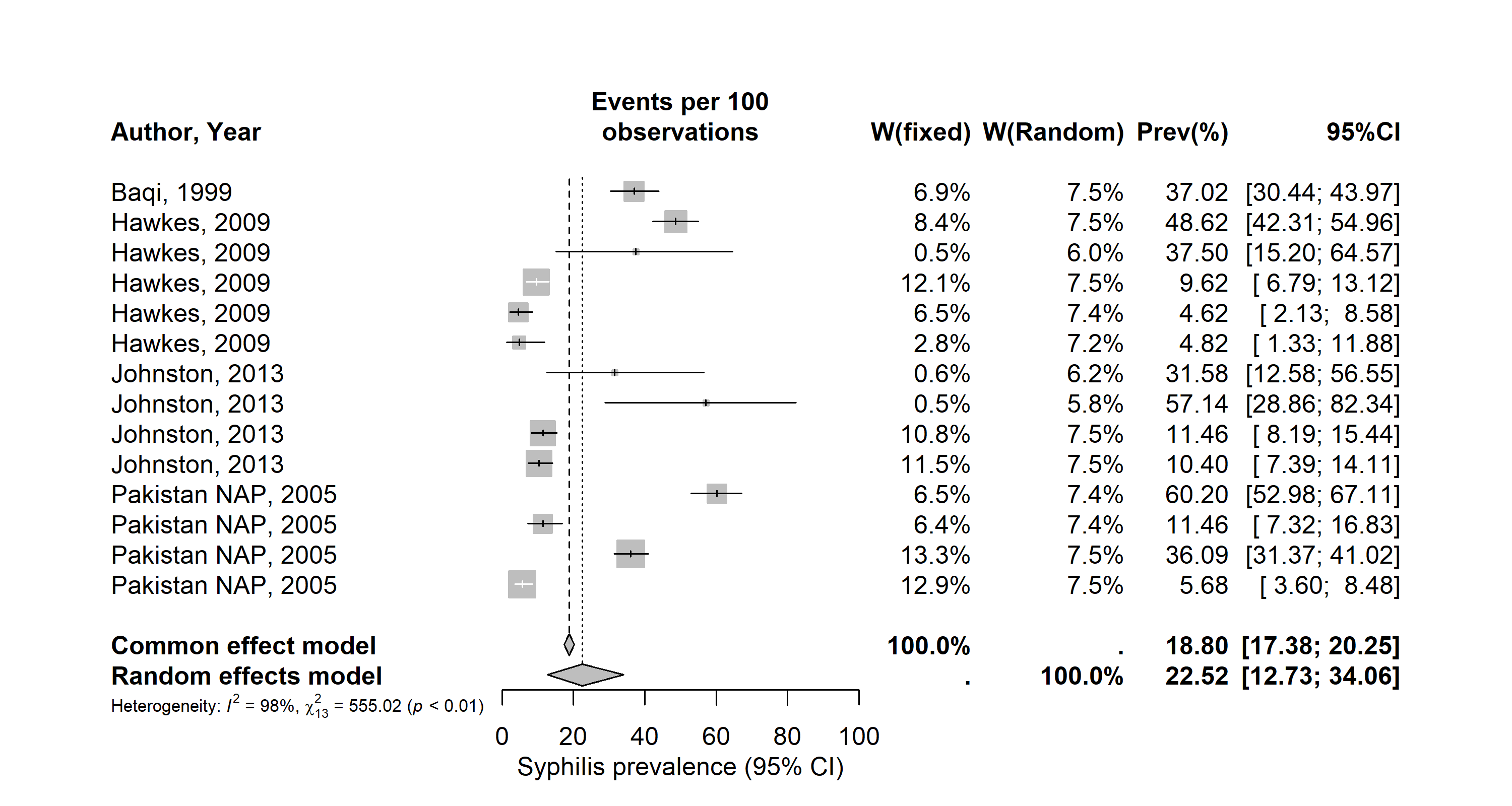


## Populations at high risk (female sex workers)


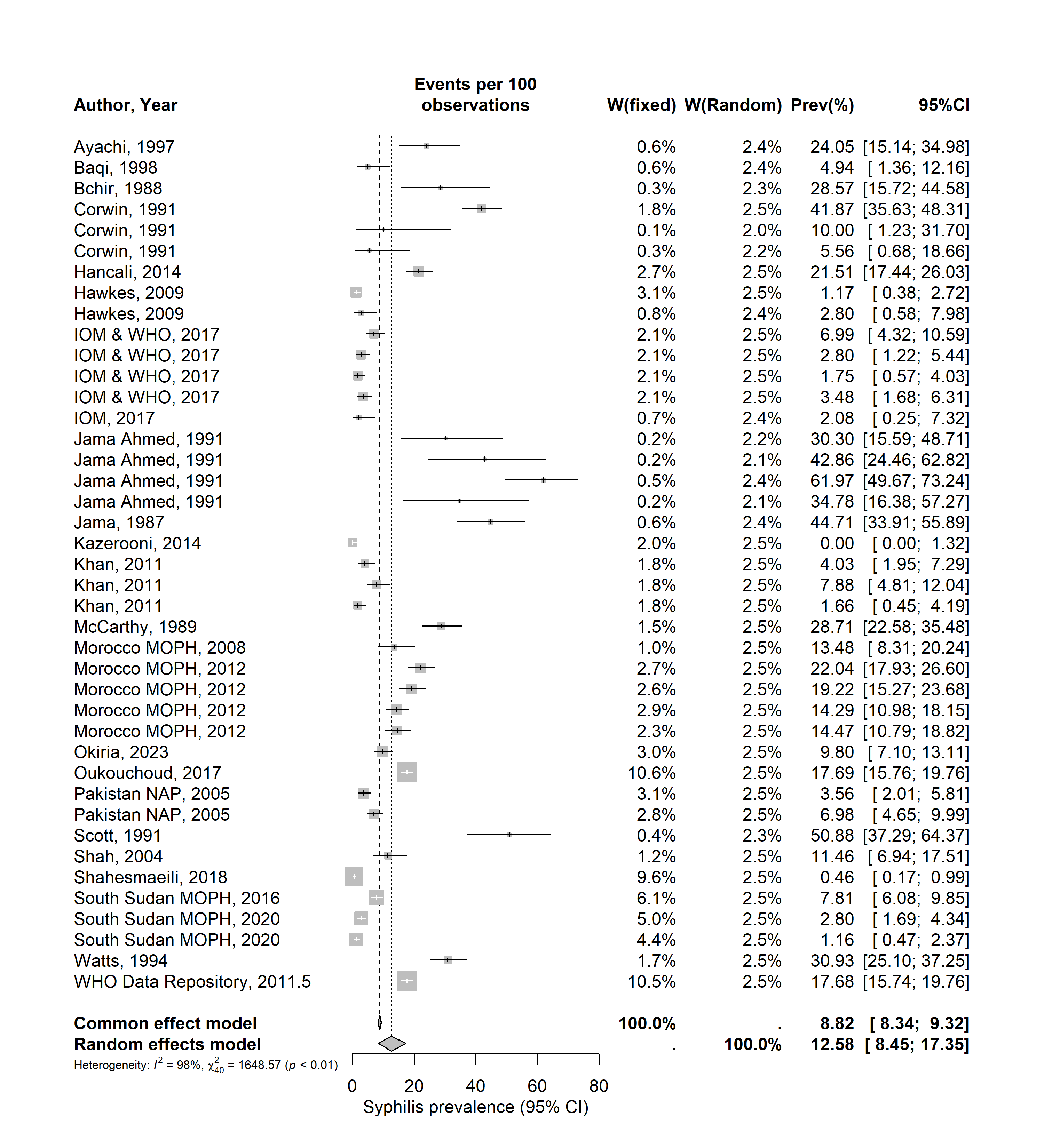


## STI clinic attendees


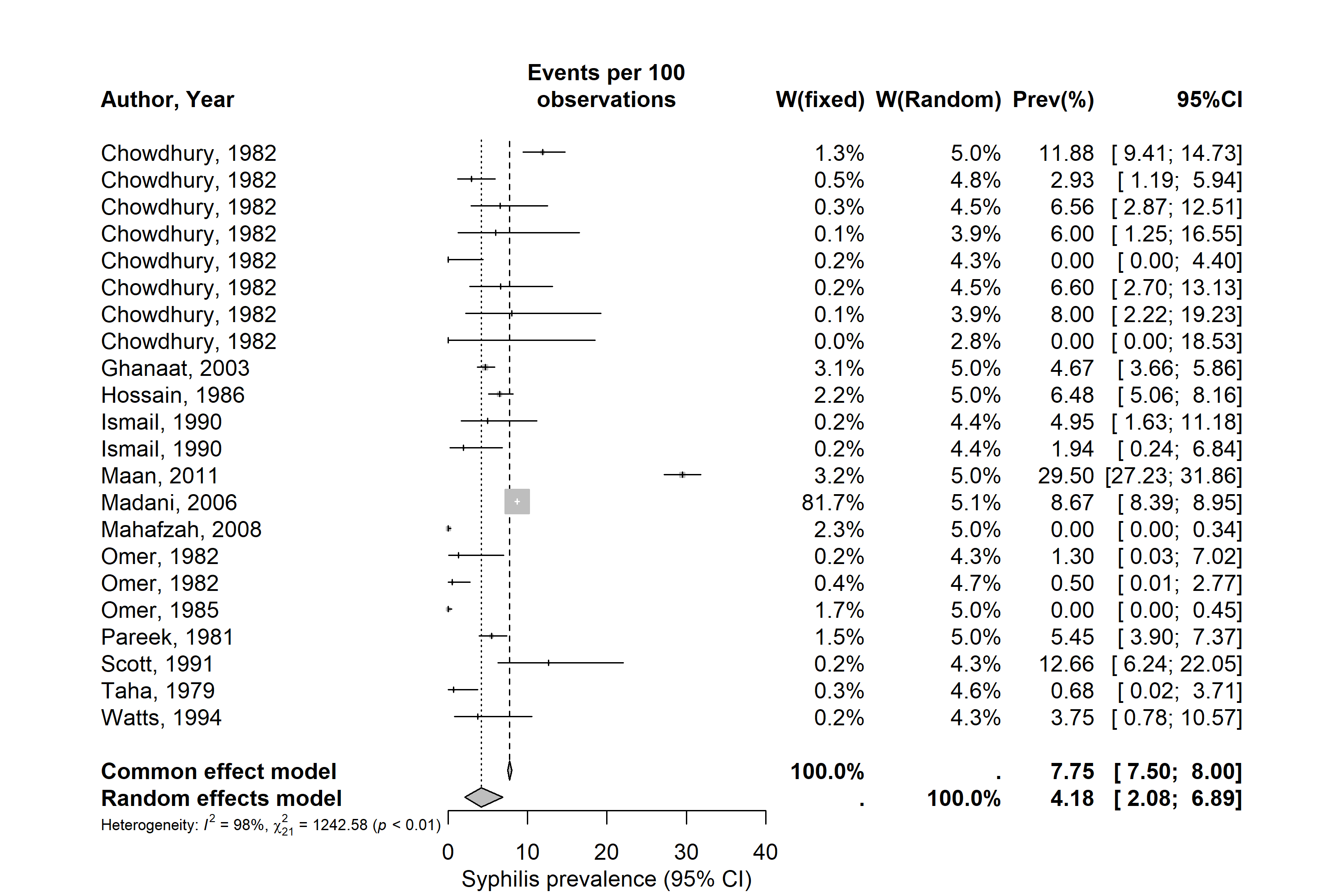


## Special clinical populations


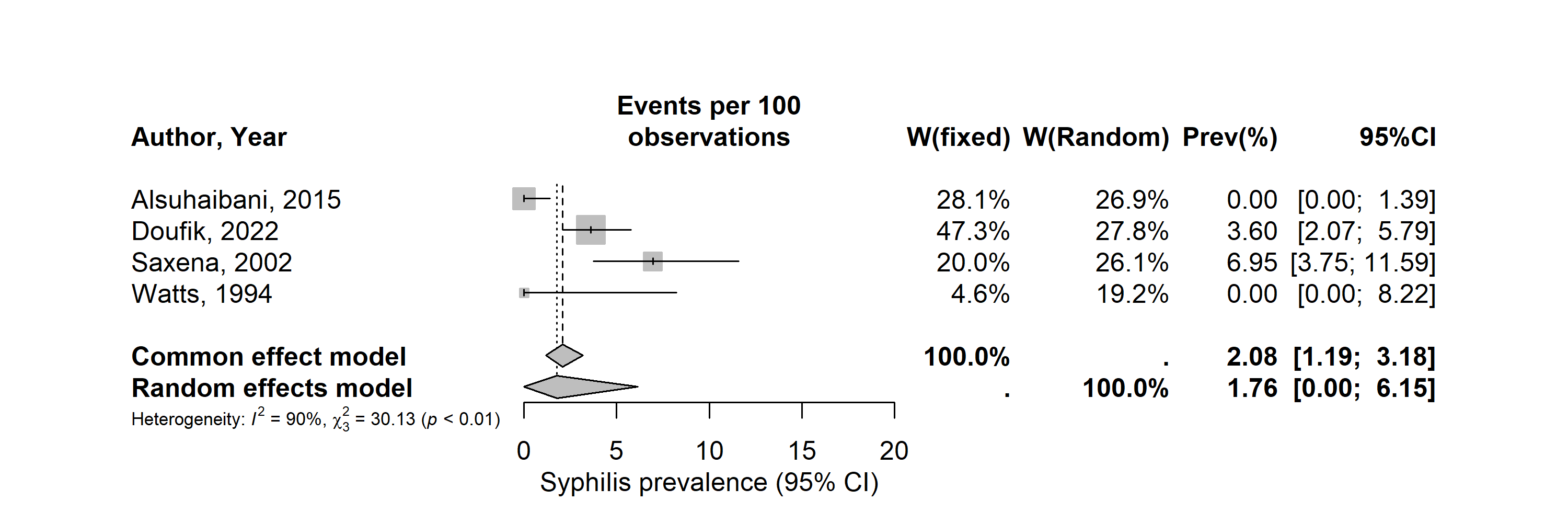


## Mixed populations


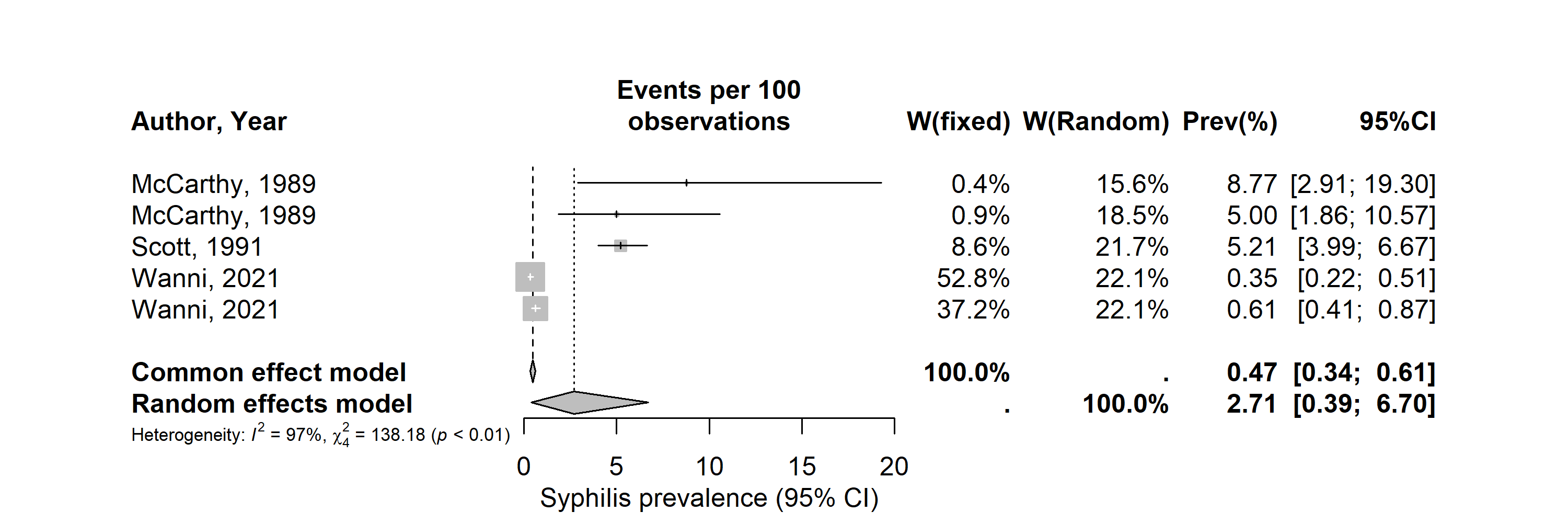


# Table S20*.* Results of meta-regression analysis to identify associations and sources of between-study heterogeneity in syphilis prevalence in the Middle East and North Africa, including all population groups and using year as a categorical variable (n=935).

|  | **Prevalence measures** | **Samples** | **Univariable analysis** | | **Variance explained** | **Multivariable analysis^*^** | |
| --- | --- | --- | --- | --- | --- | --- | --- |
|  | **N** | **Tested** | **RR (95% CI)** | **LR test p-value** | **R^2^** | **ARR (95% CI)** | **LR test p-value** |
| **Population characteristics** |  |  |  |  |  |  |  |
| **Population Type** |  |  |  |  |  |  |  |
| Blood donors | 281 | 26,511,552 | Ref | <0.001 | 36.25% | Ref | <0.001 |
| Pregnant women | 190 | 8,333,116 | 1.95 (1.39-2.74) |  |  | 1.68 (1.04-2.72) |  |
| Other general populations | 89 | 2,285,148 | 8.21 (5.39-12.52) |  |  | 2.61 (1.73-3.93) |  |
| Populations at intermediate risk | 110 | 72,436 | 7.47 (4.97-11.22) |  |  | 5.50 (3.51-8.60) |  |
| Populations at high risk | 194 | 119,516 | 22.82 (16.49-31.56) |  |  | 16.55 (10.60-25.85) |  |
| STI clinic attendees | 52 | 571,320 | 18.14 (10.64-30.94) |  |  | 8.93 (5.19-15.36) |  |
| Special clinical populations | 13 | 6,333 | 9.23 (2.95-28.85) |  |  | 5.80 (2.30-14.63) |  |
| Mixed populations | 6 | 44,315 | 10.28 (2.60-40.69) |  |  | 7.18 (2.34-22.02) |  |
| **Subregion/country^✝^** |  |  |  |  |  |  |  |
| Fertile Crescent | 78 | 3,586,219 | Ref | <0.001 | 28.25% | Ref | <0.001 |
| Eastern MENA | 138 | 16,745,891 | 1.90 (1.08-3.33) |  |  | 0.65 (0.41-1.01) |  |
| Pakistan | 227 | 10,242,587 | 10.69 (6.46-17.69) |  |  | 5.16 (3.46-7.71) |  |
| Gulf | 137 | 3,766,116 | 1.10 (0.64-1.90) |  |  | 0.86 (0.55-1.33) |  |
| North Africa | 103 | 2,456,936 | 13.12 (7.44-23.15) |  |  | 3.66 (2.29-5.83) |  |
| Horn of Africa | 252 | 1,145,987 | 16.81 (10.15-27.84) |  |  | 3.94 (2.53-6.13) |  |
| **Year of data collection** |  |  |  |  |  |  |  |
| <2000 | 133 | 719,696 | Ref | <0.001 | 9.79% | Ref | <0.001 |
| 2000-2009 | 253 | 5,297,171 | 0.44 (0.28-0.69) |  |  | 0.53 (0.36-0.78) |  |
| 2010-2019 | 493 | 27,594,398 | 0.19 (0.13-0.28) |  |  | 0.45 (0.31-0.66) |  |
| ≥2020 | 56 | 4,332,170 | 0.09 (0.05-0.18) |  |  | 0.33 (0.20-0.57) |  |
| **Sex at birth** |  |  |  |  |  |  |  |
| Men | 229 | 1,705,142 | Ref | <0.001 | 13.86% | Ref | 0.018 |
| Women | 379 | 8,464,436 | 0.50 (0.35-0.70) |  |  | 0.72 (0.52-1.01) |  |
| Mix of men and women | 258 | 26,007,723 | 0.13 (0.09-0.19) |  |  | 0.64 (0.47-0.87) |  |
| Missing | 69 | 1,766,435 | 0.23 (0.14-0.40) |  |  | 0.68 (0.44-1.07) |  |
| **Study methodology characteristics** |  |  |  |  |  |  |  |
| **Infection type^‡^** |  |  |  |  |  |  |  |
| Probable current syphilis infection | 222 | 10,380,474 | Ref | 0.901 | 0.00% | Ref | <0.001 |
| Possible current infection, unspecified | 175 | 7,694,930 | 0.91 (0.59-1.43) |  |  | 1.97 (1.41-2.75) |  |
| Lifetime syphilis infection | 383 | 8,159,488 | 1.00 (0.69-1.46) |  |  | 2.35 (1.78-3.09) |  |
| Unclear infection time | 155 | 11,708,844 | 1.10 (0.69-1.75) |  |  | 2.00 (1.42-2.81) |  |
| **Syphilis ascertainment** |  |  |  |  |  |  |  |
| Assay not explicitly described | 155 | 11,708,844 | Ref | 0.553 | 0.00% | .. |  |
| Assay explicitly described | 780 | 26,234,892 | 0.89 (0.61-1.31) |  |  | .. |  |
| **Sampling method** |  |  |  |  |  |  |  |
| Non-probability based | 543 | 16,402,298 | Ref | <0.001 | 10.13% | Ref | 0.025 |
| Probability based | 186 | 79,278 | 3.47 (2.43-4.96) |  |  | 0.64 (0.46-0.90) |  |
| Missing | 206 | 21,462,160 | 0.44 (0.31-0.62) |  |  | 0.81 (0.60-1.09) |  |
| **Precision** |  |  |  |  |  |  |  |
| Low precision | 325 | 65,325 | Ref | <0.001 | 15.60% | Ref | <0.001 |
| High precision | 610 | 37,878,410 | 0.17 (0.13-0.23) |  |  | 0.42 (0.33-0.55) |  |

ARR: Adjusted risk ratio, CI: Confidence interval, LR: Likelihood ratio, MENA: Middle East and North Africa, RR: Risk ratio, R^2^: Coefficient of determination, STI: sexually transmitted infection.

The RR represents the exponentiated beta coefficient calculated by the meta-regression model. Given that prevalence can be interpreted as the probability of an individual in the population being infected, a prevalence ratio can be interpreted as a risk ratio. Hence, we have opted to use the term "risk ratio" rather than "prevalence ratio" for epidemiological relevance.

**^*^** R^2^ = 61.9%.

^✝^ These were ordered according to the hierarchy of prevalence of STIs as commonly observed in previous studies in MENA.^297,298^

**^‡^** Included in the multivariable model despite non-significance in the univariate analysis because of epidemiological relevance

Fertile Crescent includes Egypt, Iraq, Jordan, Lebanon, Palestine, Syria; North Africa includes Algeria, Libya, Morocco, Tunisia; Gulf includes: Bahrain, Kuwait, Oman, Qatar, Saudi Arabia, and United Arab Emirates; Horn of Africa includes: Djibouti, Somalia, Sudan, recently independent South Sudan, and Yemen.

# Table S21. Results of meta-regression analysis to identify associations and sources of between-study heterogeneity in syphilis prevalence in the Middle East and North Africa, including only blood donors, pregnant women, and other general population groups and using year as a categorical variable (n=560).

|  | **Prevalence measures** | **Samples** | **Univariable analysis** | | **Variance explained** | **Multivariable analysis^*^** | |
| --- | --- | --- | --- | --- | --- | --- | --- |
|  | **N** | **Tested** | **RR (95% CI)** | **LR test p-value** | **R^2^** | **ARR (95% CI)** | **LR test p-value** |
| **Population characteristics** |  |  |  |  |  |  |  |
| **Population Type** |  |  |  |  |  |  |  |
| Blood donors | 281 | 26,511,552 | Ref | <0.001 | 13.18% | Ref | 0.001 |
| Pregnant women | 190 | 8,333,116 | 1.91 (1.30-2.81) |  |  | 1.40 (0.65-2.99) |  |
| Other general populations | 89 | 2,285,148 | 8.22 (5.07-13.33) |  |  | 2.35 (1.48-3.72) |  |
| **Subregion/country^✝^** |  |  |  |  |  |  |  |
| Fertile Crescent | 65 | 3,572,481 | Ref | <0.001 | 50.31% | Ref | <0.001 |
| Eastern MENA | 58 | 16,168,072 | 0.24 (0.13-0.44) |  |  | 0.29 (0.16-0.51) |  |
| Pakistan | 169 | 10,219,437 | 8.28 (5.23-13.12) |  |  | 4.57 (2.89-7.23) |  |
| Gulf | 100 | 3,685,834 | 0.69 (0.41-1.14) |  |  | 0.79 (0.49-1.30) |  |
| North Africa | 40 | 2,397,224 | 2.52 (1.36-4.66) |  |  | 2.54 (1.43-4.51) |  |
| Horn of Africa | 128 | 1,086,768 | 18.88 (11.59-30.75) |  |  | 12.44 (7.22-21.44) |  |
| **Year of data collection** |  |  |  |  |  |  |  |
| <2000 | 50 | 115,319 | Ref | 0.000 | 8.54% | Ref | <0.001 |
| 2000-2009 | 164 | 5259,125 | 0.78 (0.39-1.54) |  |  | 0.35 (0.21-0.57) |  |
| 2010-2019 | 310 | 27,450,302 | 0.26 (0.14-0.49) |  |  | 0.50 (0.29-0.84) |  |
| ≥2020 | 36 | 4,305,070 | 0.13 (0.05-0.32) |  |  | 0.40 (0.20-0.78) |  |
| **Sex at birth** |  |  |  |  |  |  |  |
| Men | 76 | 1,611,859 | Ref | <0.001 | 7.88% | Ref | 0.001 |
| Women | 220 | 8,380,276 | 0.34 (0.20-0.59) |  |  | 0.38 (0.18-0.79) |  |
| Mix of men and women | 202 | 25,407,283 | 0.17 (0.10-0.28) |  |  | 0.50 (0.33-0.74) |  |
| Missing | 62 | 1,730,399 | 0.38 (0.19-0.75) |  |  | 0.56 (0.33-0.94) |  |
| **Study methodology characteristics** |  |  |  |  |  |  |  |
| **Infection type** |  |  |  |  |  |  |  |
| Probable current syphilis infection | 95 | 10,273,824 | Ref | <0.001 | 9.42% | Ref | <0.001 |
| Possible current infection, unspecified | 146 | 7,651,489 | 7.00 (4.00-12.27) |  |  | 2.89 (1.87-4.45) |  |
| Lifetime syphilis infection | 246 | 7,578,612 | 5.22 (3.10-8.80) |  |  | 2.68 (1.76-4.10) |  |
| Unclear infection time | 73 | 11,625,891 | 2.19 (1.14-4.21) |  |  | 2.38 (1.43-3.96) |  |
| **Syphilis ascertainment^‡^** |  |  |  |  |  |  |  |
| Assay not explicitly described | 73 | 11,625,891 | Ref | 0.013 | 1.06% | .. |  |
| Assay explicitly described | 487 | 25,503,925 | 1.97 (1.15-3.36) |  |  | .. |  |
| **Sampling method** |  |  |  |  |  |  |  |
| Non-probability based | 405 | 15,727,994 | Ref | <0.001 | 7.31% | Ref | 0.442 |
| Probability based | 13 | 14,854 | 1.53 (0.43-5.46) |  |  | 0.63 (0.26-1.54) |  |
| Missing | 142 | 21,386,968 | 0.27 (0.18-0.41) |  |  | 0.85 (0.58-1.24) |  |
| **Precision** |  |  |  |  |  |  |  |
| Low precision | 140 | 33,993 | Ref | <0.001 | 19.41% | Ref | 0.001 |
| High precision | 420 | 37,095,823 | 0.11 (0.08-0.17) |  |  | 0.35 (0.24-0.51) |  |

ARR: Adjusted risk ratio, CI: Confidence interval, LR: Likelihood ratio, MENA: Middle East and North Africa, RR: Risk ratio, R^2^: Coefficient of determination, STI: sexually transmitted infection.

The RR represents the exponentiated beta coefficient calculated by the meta-regression model. Given that prevalence can be interpreted as the probability of an individual in the population being infected, a prevalence ratio can be interpreted as a risk ratio. Hence, we have opted to use the term "risk ratio" rather than "prevalence ratio" for epidemiological relevance.

**^*^** R^2^ = 62.7%.

^✝^ These were ordered according to the hierarchy of prevalence of STIs as commonly observed in previous studies in MENA.^297,298^

^‡^ Not included in the multivariable model because of identified collinearity with infection type.

Fertile Crescent includes Egypt, Iraq, Jordan, Lebanon, Palestine, Syria; North Africa includes Algeria, Libya, Morocco, Tunisia; Gulf includes: Bahrain, Kuwait, Oman, Qatar, Saudi Arabia, and United Arab Emirates; Horn of Africa includes: Djibouti, Somalia, Sudan, recently independent South Sudan, and Yemen.

# Table S22. Results of meta-regression analysis to identify associations and sources of between-study heterogeneity in syphilis prevalence in the Middle East and North Africa, including only populations at high risk (n=194).

|  | **Prevalence measures** | **Samples** | **Univariable analysis** | | **Variance explained** | **Multivariable analysis^*^** | |
| --- | --- | --- | --- | --- | --- | --- | --- |
|  | **N** | **Tested** | **RR (95% CI)** | **LR test p-value** | **R^2^** | **ARR (95% CI)** | **LR test p-value** |
| **Population characteristics** |  |  |  |  |  |  |  |
| **Subregion/country^✝^** |  |  |  |  |  |  |  |
| Fertile Crescent | 5 | 2,244 | Ref | <0.001 | 21.12% | Ref | <0.001 |
| Eastern MENA | 33 | 40,395 | 1.21 (0.33-4.45) |  |  | 0.97 (0.28-3.38) |  |
| Pakistan | 32 | 11,736 | 7.75 (2.17-27.64) |  |  | 3.05 (0.88-10.56) |  |
| Gulf | 1 | 26 | 2.19 (0.10-50.04) |  |  | 1.37 (0.08-24.02) |  |
| North Africa | 45 | 17,140 | 6.21 (1.77-21.82) |  |  | 5.34 (1.48-19.25) |  |
| Horn of Africa | 78 | 47,975 | 3.49 (1.01-12.03) |  |  | 1.77 (0.52-5.98) |  |
| **Year of data collection** |  |  |  |  |  |  |  |
| As a linear term | 194 | 119,516 | 0.92 (0.91-0.94) | <0.001 | 32.90% | 0.92 (0.89-0.94) | <0.001 |
| **Sub-population^‡^** |  |  |  |  |  |  |  |
| MSM & transgender people | 65 | 53,583 | Ref | 0.349 | 0.00% | Ref | 0.257 |
| FSW | 126 | 65,407 | 0.95 (0.64-1.40) |  |  | 0.78 (0.56-1.07) |  |
| Mix/unclear | 3 | 526 | 0.13 (0.01-2.08) |  |  | 0.55 (0.05-6.52) |  |
| **Study methodology characteristics** |  |  |  |  |  |  |  |
| **Infection type** |  |  |  |  |  |  |  |
| Probable current syphilis infection | 55 | 16,655 | Ref | 0.005 | 5.51% | Ref | 0.201 |
| Possible current infection, unspecified | 10 | 5,104 | 0.42 (0.17-1.00) |  |  | 2.18 (1.06-4.50) |  |
| Lifetime syphilis infection | 64 | 22,923 | 0.48 (0.30-0.76) |  |  | 1.09 (0.75-1.60) |  |
| Unclear infection time | 65 | 74,834 | 0.50 (0.32-0.79) |  |  | 1.09 (0.68-1.75) |  |
| **Syphilis ascertainment^§^** |  |  |  |  |  |  |  |
| Assay not explicitly described | 65 | 74,834 | Ref | 0.156 | 0.72% | .. |  |
| Assay explicitly described | 129 | 44,682 | 1.33 (0.90-1.97) |  |  | .. |  |
| **Sampling method** |  |  |  |  |  |  |  |
| Non-probability based | 42 | 14,767 | Ref | <0.001 | 8.77% | Ref | 0.596 |
| Probability based | 115 | 46,954 | 0.43 (0.28-0.68) |  |  | 1.01 (0.65-1.59) |  |
| Missing | 37 | 57,795 | 0.37 (0.21-0.64) |  |  | 1.28 (0.73-2.26) |  |
| **Precision** |  |  |  |  |  |  |  |
| Low precision | 55 | 5,406 | Ref | <0.001 | 13.66% | Ref | 0.447 |
| High precision | 139 | 114,110 | 0.37 (0.25-0.54) |  |  | 0.86 (0.59-1.26) |  |

ARR: Adjusted risk ratio, CI: Confidence interval, FSW: Female sex workers, LR: Likelihood ratio, MENA: Middle East and North Africa, MSM: Men who have sex with men, RR: Risk ratio, R^2^: Coefficient of determination.

The RR represents the exponentiated beta coefficient calculated by the meta-regression model. Given that prevalence can be interpreted as the probability of an individual in the population being infected, a prevalence ratio can be interpreted as a risk ratio. Hence, we have opted to use the term "risk ratio" rather than "prevalence ratio" for epidemiological relevance.

**^*^** R^2^ = 51.9%.

^✝^ These were ordered according to the hierarchy of prevalence of STIs as commonly observed in previous studies in MENA.^297,298^

**^‡^** Included in the multivariable model despite non-significance in the univariable analysis because of epidemiological relevance.

**^§^** Not included in the multivariable model because of identified collinearity with infection type.

Fertile Crescent includes Egypt, Iraq, Jordan, Lebanon, Palestine, Syria; North Africa includes Algeria, Libya, Morocco, Tunisia; Gulf includes: Bahrain, Kuwait, Oman, Qatar, Saudi Arabia, and United Arab Emirates; Horn of Africa includes: Djibouti, Somalia, Sudan, recently independent South Sudan, and Yemen.

# Table S23. Results of meta-regression analysis to identify associations and sources of between-study heterogeneity in syphilis prevalence in the Middle East and North Africa, including only populations at high risk and using year as a categorical variable (n=192).

|  | **Prevalence measures** | **Samples** | **Univariable analysis** | | **Variance explained** | **Multivariable analysis^*^** | |
| --- | --- | --- | --- | --- | --- | --- | --- |
|  | **N** | **Tested** | **RR (95% CI)** | **LR test p-value** | **R^2^** | **ARR (95% CI)** | **LR test p-value** |
| **Population characteristics** |  |  |  |  |  |  |  |
| **Subregion/country**^✝^ |  |  |  |  |  |  |  |
| Fertile Crescent | 5 | 2,244 | Ref | <0.001 | 21.12% | Ref | <0.001 |
| Eastern MENA | 33 | 40,395 | 1.21 (0.33-4.45) |  |  | 1.77 (0.50-6.31) |  |
| Pakistan | 32 | 11,736 | 7.75 (2.17-27.64) |  |  | 7.6 (1.94-29.81) |  |
| Gulf | 1 | 26 | 2.19 (0.10-50.04) |  |  | 1.7 (0.09-30.61) |  |
| North Africa | 45 | 17,140 | 6.21 (1.77-21.82) |  |  | 9.35 (2.54-34.37) |  |
| Horn of Africa | 78 | 47,975 | 3.49 (1.01-12.03) |  |  | 2.79 (0.82-9.56) |  |
| **Year of data collection** |  |  |  |  |  |  |  |
| <2000 | 21 | 1,776 | Ref | <0.001 | 29.58% | Ref | <0.001 |
| 2000-2009 | 36 | 9,446 | 0.37 (0.21-0.66) |  |  | 0.15 (0.06-0.35) |  |
| 2010-2019 | 121 | 82,961 | 0.17 (0.10-0.27) |  |  | 0.12 (0.06-0.25) |  |
| ≥2020 | 16 | 25,333 | 0.05 (0.02-0.11) |  |  | 0.04 (0.01-0.09) |  |
| **Sub-population^‡^** |  |  |  |  |  |  |  |
| MSM & transgender people | 65 | 53,583 | Ref | 0.349 | 0.00% | Ref | 0.074 |
| FSW | 126 | 65,407 | 0.95 (0.64-1.4) |  |  | 0.69 (0.49-0.96) |  |
| Mix/unclear | 3 | 526 | 0.13 (0.01-2.08) |  |  | 0.46 (0.04-5.63) |  |
| **Study methodology characteristics** |  |  |  |  |  |  |  |
| **Infection type** |  |  |  |  |  |  |  |
| Probable current syphilis infection | 55 | 16,655 | Ref | 0.005 | 5.51% | Ref | 0.208 |
| Possible current infection, unspecified | 10 | 5,104 | 0.42 (0.17-1.00) |  |  | 2.22 (1.05-4.68) |  |
| Lifetime syphilis infection | 64 | 22,923 | 0.48 (0.30-0.76) |  |  | 1.27 (0.84-1.9) |  |
| Unclear infection time | 65 | 74,834 | 0.50 (0.32-0.79) |  |  | 1.07 (0.66-1.72) |  |
| **Syphilis ascertainment^§^** |  |  |  |  |  |  |  |
| Assay not explicitly described | 65 | 74,834 | Ref | 0.156 | 0.72% | .. |  |
| Assay explicitly described | 129 | 44,682 | 1.33 (0.9-1.97) |  |  | .. |  |
| **Sampling method** |  |  |  |  |  |  |  |
| Non-probability based | 42 | 14,767 | Ref | <0.001 | 8.77% | Ref | 0.649 |
| Probability based | 115 | 46,954 | 0.43 (0.28-0.68) |  |  | 1.08 (0.65-1.82) |  |
| Missing | 37 | 57,795 | 0.37 (0.21-0.64) |  |  | 1.32 (0.71-2.45) |  |
| **Precision** |  |  |  |  |  |  |  |
| Low precision | 55 | 5,406 | Ref | <0.001 | 13.66% | Ref | 0.312 |
| High precision | 139 | 114,110 | 0.37 (0.25-0.54) |  |  | 0.82 (0.56-1.21) |  |

ARR: Adjusted risk ratio, CI: Confidence interval, FSW: Female sex workers, LR: Likelihood ratio, MENA: Middle East and North Africa, MSM: Men who have sex with men, RR: Risk ratio, R^2^: Coefficient of determination.

The RR represents the exponentiated beta coefficient calculated by the meta-regression model. Given that prevalence can be interpreted as the probability of an individual in the population being infected, a prevalence ratio can be interpreted as a risk ratio. Hence, we have opted to use the term "risk ratio" rather than "prevalence ratio" for epidemiological relevance.

**^*^** R^2^ = 50.3%.

^✝^ These were ordered according to the hierarchy of prevalence of STIs as commonly observed in previous studies in MENA.^297,298^

**^‡^** Included in the multivariable model despite non-significance in the univariable analysis because of epidemiological relevance.

**^§^** Not included in the multivariable model because of identified collinearity with infection type.

Fertile Crescent includes Egypt, Iraq, Jordan, Lebanon, Palestine, Syria; North Africa includes Algeria, Libya, Morocco, Tunisia; Gulf includes: Bahrain, Kuwait, Oman, Qatar, Saudi Arabia, and United Arab Emirates; Horn of Africa includes: Djibouti, Somalia, Sudan, recently independent South Sudan, and Yemen.

# References

1. Morshed MG, Singh AE. Recent trends in the serologic diagnosis of syphilis. *Clin Vaccine Immunol* 2015; **22**(2): 137-47.

2. World Health Organization. WHO guidelines on syphilis screening and treatment for pregnant women. Available at <https://www.who.int/publications/i/item/9789241550093>. Last accessed May 20, 2024. 2017.

3. Ham DC, Lin C, Newman L, Wijesooriya NS, Kamb M. Improving global estimates of syphilis in pregnancy by diagnostic test type: A systematic review and meta-analysis. *Int J Gynaecol Obstet* 2015; **130 Suppl 1**(0 1): S10-4.

4. Keshvari M, Sharafi H, Alavian SM, Mehrabadi H, Zolfaghari S. Prevalence and trends of transfusion-transmitted infections among blood donors in Tehran, Iran from 2008 to 2013. *Transfusion and Apheresis Science* 2015; **53**(1): 38-47.

5. Khedmat H, Fallahian F, Abolghasemi H, et al. Seroepidemiologic study of hepatitis B virus, hepatitis C virus, human immunodeficiency virus and syphilis infections in Iranian blood donors. *Pakistan Journal of Biological Sciences* 2007; **10**(24): 4461-6.

6. Khedmat H, Alavian SM, Miri SM, et al. Trends in seroprevalence of hepatitis B, hepatitis C, HIV, and syphilis infections in Iranian blood donors from 2003 to 2005. *Hepatitis Monthly* 2009; **9**(1): 24-8.

7. Khoda AO, Gharehbaghian A, Jamali M, et al. Comparison of the prevalence of major transfusion-transmitted infections among iranian blood donors using confidential unit exclusion in an Iranian population. *Hepatitis Monthly* 2011; **11**(1): 11-3.

8. Mohammadali F. Changes in frequency of HBV, HCV, HIV and syphilis infections among blood donors in Tehran province 2005–2011. *Arch Iran Med* 2014; **17**(9): 613.

9. A.M AL-E, K.M AL-J, Abbas YA. Epidemiological Study for Prevalence and Incidence of Syphilis Among Blood Donors in Thi-qar Province. *Biochemical and Cellular Archives* 2018; **18(1)**: 65-8.

10. Alaidarous M, Choudhary RK, Waly MI, et al. The prevalence of transfusion-transmitted infections and nucleic acid testing among blood donors in Majmaah, Saudi Arabia. *Journal of Infection and Public Health* 2018.

11. Alshehri OM, Nahari MH, Hassan EE, Alqahtani MF, Awaji TH. Prevalence of ABO, Rh and KELL blood group types and Transfusion- Transmissible Infections (TTI) among blood donors in Najran City, Saudi Arabia. *Biomedical and Pharmacology Journal* 2021; **14(2)**: 1065-76.

12. Hossain A. Serological tests for syphilis in Saudi Arabia. *Genitourinary Medicine* 1986; **62**(5): 293-7.

13. Omer E, El Sheikh FS, Khalil IA. Evaluation of VDRL test in Sudanese blood donors. *Tropical doctor* 1982; **12**(2): 61-2.

14. El-Gilany AH, El-Fedawy S. Bloodborne infections among student voluntary blood donors in Mansoura University, Egypt. *Eastern Mediterranean Health Journal* 2006; **12**(6): 742-8.

15. Nada H, Atwa M. Seroprevalence of HBV, HCV, HIV and syphilis markers among blood donors at Suez Canal University Hospital Blood Bank. *Blood Disord Transfus* 2013; **5**: 177.

16. Emadi B, Ghahraman Rezaieh M, Sedighi M. Seroprevalence and trends of transfusion transmissible infections among retrospective blood donors in Western Azerbaijan Regional Blood Transfusion Center, Iran: A ten-years evaluation. *Transfusion and Apheresis Science* 2021; **60(3) (no pagination)**.

17. Al-Alwani HR. Seropositive prevalence of transfusion transmitted infections among blood donors in Al-anbar province after war displacement period. *Journal of Pharmaceutical Sciences and Research* 2018; **10**(12): 3333-5.

18. Alabdulmonem W, Shariq A, Alqossayir F, et al. Sero-prevalence ABO and Rh blood groups and their associated Transfusion-Transmissible Infections among Blood Donors in the Central Region of Saudi Arabia. *Journal of infection and public health* 2020; **13**(2): 299-305.

19. Alcantara JC, Alenezi FKM, Haj Ali OH. Seroprevalence and trends of markers of transfusion transmissible infections among blood donors: a 3-year hospital based-study. *2018* 2018; **5**(12): 5.

20. Bashawri LA. Pattern of blood procurement, ordering and utilization in a University Hospital in Eastern Saudi Arabia. *Saudi Medical Journal* 2002; **23**(5): 555-61.

21. Sarah YAEGA, Sabry AEGAEHES, Maryam AALS. Seropositivity of TTIs among blood donors in Hail, Saudi Arabia, from 2014 to 2015. *Asian Pacific Journal of Tropical Disease* 2016; **6**(2): 141-6.

22. Attaullah S, Khan S, Khan J. Trend of transfusion transmitted infections frequency in blood donors: Provide a road map for its prevention and control. *Journal of Translational Medicine* 2012: 20.

23. Karim F, Grabowski K, Qadir H, Bloch E. Seroprevalence of HIV, HBV, HCV, syphilis and malaria among blood donors at a large academic center in Pakistan: 2005-2016. *Vox Sanguinis* 2018; **113 (Supplement 1)**: 192.

24. Khan S. Improving the safety of blood products through stringent donor selection, pre and post donation screening of blood. *Vox Sanguinis* 2012; **1)**: 98-9.

25. Manzoor I, Hashmi N, Daud S, et al. Seroprevalence of transfusion transmissible infections (TTIS) in blood donors. *Biomedica* 2009; **25**(10): 154-8.

26. Nepal B. Frequency of reactive blood donors in a tertiary care hospital, Karachi, Pakistan. *Haematologica* 2016; **101 (Supplement 1)**: 649-50.

27. Qadeer MI, Hasnain S, Yasmeen H. Sero-prevalence of sexually transmitted disease (Hiv, Syphilis, Hepatitis-B and Hepatitis-C) in Volunteer Donors of Gaol Inmates and Student Community in Punjab Province of Pakistan. *Sexually Transmitted Infections Conference: STI and AIDS World Congress* 2013; **89**(SUPPL. 1).

28. Sultan S, Murad S, Irfan SM, Biag MA. Trends of venereal infections among healthy blood donors at Karachi. *Archives of Iranian Medicine* 2016; **19**(3): 192-6.

29. Sultan F, Mehmood T, Mahmood MT. Infectious pathogens in volunteer and replacement blood donors in Pakistan: a ten-year experience. *International Journal of Infectious Diseases* 2007; **11**(5): 407-12.

30. Waheed U, Zaheer HA, Astori S. Transfusion transmitted infections among blood donors of a teaching hospital in Islamabad, Pakistan. *Vox Sanguinis* 2012; **1)**: 157.

31. Zehra SK, Bano S, Hakim ST, Nadeem SG. Prevalence of transfusion transmitted infection in replacement and voluntary blood donor. *RADS Journal of Biological Research & Applied Sciences* 2013; **4**(2): 56-63.

32. Djoudi F, Amir N, Chouikh MT. Transfusion-transmissible infections among blood donors in Bejaia, Algeria: ten years retrospective and comparative study. *Journal of Infection in Developing Countries* 2023; **17**(6): 840-5.

33. Hashemi E, Waheed U, Saba N, Wazeer A. First Report from Afghanistan on the Prevalence of Blood-Borne Infections: A Retrospective Cross-Sectional Multicentre Study for an Epidemiological Assessment. *J Blood Med* 2022; **13**: 45-50.

34. Mansoor GF, Rahmani AM, Kakar MA, et al. Blood supply safety in Afghanistan: A national assessment of high-volume facilities. *Transfusion* 2013; **53**(9): 2061-8.

35. Abdel Messih IY, Ismail MA, Saad AA, Azer MR. The degree of safety of family replacement donors versus voluntary non-remunerated donors in an Egyptian population: A comparative study. *Blood Transfusion* 2014; **12**(2): 159-65.

36. Ashour D, Moftah F, Gobran H, Ekram D. Decreasing the risk of transfusion transmitted infections (TTIS) in the Egyptian Blood Transfusion Services. *Vox Sanguinis* 2009; **1)**: 103-4.

37. Farouk Badr R, Farouk R, Ahmed Ali A. Evaluation of nucleic acid testing to detect HBV infections in egyption blood donors. *Vox Sanguinis* 2015; **1)**: 210-1.

38. Niazkar HR, Dorgalaleh A, Rad F. First-Time Blood Donors are Double Edged Swords for Blood Transfusion Centers: a Retrospective Study in Southwest Iran. *Turkish journal of haematology : official journal of Turkish Society of Haematology* 2019; **02**.

39. Abdullah D. Khairallah AS Mohammed Detection of Infectious Agents in Blood Donors in Basra, Iraq. *Int J Adv Res* 2016; **4**(10): 1251-3.

40. Hassan SA, Nassir ES, Ibrahim AI, Tareq L. Seroprevalence of syphilis among iraq's blood donors and their relation with blood group and rh factor. *Indian Journal of Forensic Medicine and Toxicology* 2020; **14(4)**: 1700-6.

41. Hassan SH. Prevalence of syphilis in blood donors over one year in Karbala Governorate, Iraq. *Indian Journal of Public Health Research and Development* 2020; **11(4)**: 1551-5.

42. Saleh MA. Evaluating the prevalence of specific Treponemal antibodies in Iraqi blood donor. *Diyala journal for pure sciences* 2010; **6**(4): 240-9.

43. Mohammed KAS, Hameed MM, Mousa AH, Saleh AT. Prevalence and trends of transfusion transmissible infections among blood donors in Basra, Iraq. *BMJ Open Gastroenterology* 2023; **10**(1): e000968.

44. Hroob AMA, Saghir SAM, Almaiman AA, et al. Prevalence and Association of Transfusion Transmitted Infections with ABO and Rh Blood Groups among Blood Donors at the National Blood Bank, Amman, Jordan. *Medicina* 2020; **56**(12).

45. Souan L, Tout F, Siag M, Sughayer MA. Seroprevalence rates of transfusion-transmitted infections among blood donors in Jordan. *Journal of Infection in Developing Countries* 2016; **10**(4): 377-83.

46. Al-Ahmadi RA, Bukhari AA, Alsharyfi AM, et al. PREVALENCE OF TRANSFUSION TRANSMISSIBLE DISEASES BETWEEN MADINAH BLOOD BANK DONORS, SAUDI ARABIA. *Journal of Population Therapeutics and Clinical Pharmacology* 2023; **30**(18): 2456-66.

47. Almaiman AA, Almaiman SH. Evaluation of Blood Donors and transfusion transmitted infections and their association with ABO and Rh Blood groups in Unaizah, Saudi Arabia: A retrospective study. *Int J Med Res Health Sci* 2018; **7**(3): 143-50.

48. Alsughayyir J, Almalki Y, Alburayk I, et al. Prevalence of transfusion-transmitted infections in Saudi Arabia blood donors: A nationwide, cross-sectional study. *Saudi Med J* 2022; **43**(12): 1363-72.

49. Elyamany G, Al Amro M, Pereira WC, Alsuhaibani O. Prevalence of Syphilis among Blood and Stem Cell Donors in Saudi Arabia: An Institutional Experience. *Electronic physician* 2016; **8**(8): 2747-51.

50. Kabrah SM, Alandijany TA, Felimban RI, Alserihi RF, Theyab A, Ebid GT. The Prevalence of Transfusion-Transmitted Infection Markers among Blood Donors at Saudi Hospital, Makkah. *Clin Lab* 2023; **69**(2).

51. Kilany M, Bin Dajem SM, Ibrahim YM, Alshehri A, Aljeamelani AA, Ibrahim EH. Seroprevalence of anti-Treponemapallidum antibodies (Syphilis) in blood donors in the southern area of Saudi Arabia. *Research Journal of Pharmaceutical, Biological and Chemical Sciences* 2015; **6**(1): 549-56.

52. Shaikh AA, Alqasem HM, Alshubruqi YA, Alasmari SZ, Makkawi MH. Association of ABO, Rh-D and Kell blood groups with transfusion transmitted infections among blood donors from the Asir Region, Saudi Arabia: A retrospective observational study. *Saudi Med J* 2024; **45**(4): 414-23.

53. Ali MS, Qowaider S, Moftah S. Seroprevalence rates of transfusion-transmitted infections among blood donors in northeast of Libya. *J Sci Humanit* 2014; **19**: 1-7.

54. Laouina A, Alami R, Adouani B, et al. Prevalence of transmissible infectious markers transfusion among blood donors in Morocco. *Vox Sanguinis* 2016; **111 (Supplement 1)**: 172.

55. Ahmad M, Saeed M, Hanif A, Waheed U, Arshad M, Rasheed F. Slump of trends in transfusion-transmissible infectious diseases: Is syphilis alarming in Pakistan? *Vox Sanguinis* 2019; **114(SUPPL 2)**: 77-8.

56. Ahmad M, Saeed M, Rasheed F, Aamir Hanif M, Waheed U, Arshad Malik M. Challenges in blood transfusion practices in hospital blood bank, lahore. *Vox Sanguinis* 2020; **115(SUPPL 1)**: 78-9.

57. Ali SM, Raza N, Irfan M, Mohammad MF, Kazmi FH, Fatima Z. Effectiveness of Using Nucleic Acid Amplification Test to Screen Blood Donors for Hepatitis B, Hepatitis C, and HIV: A Tertiary Care Hospital Experience From Pakistan. *Cureus* 2023; **15**(1): e34216.

58. Amin H, Jafar HS, Sadiq F, et al. Prevalence of Syphilis among the Healthy Blood Donors - A Hospital based Retrospective Study. *Pakistan Journal of Medical and Health Sciences* 2019; **13**(3): 647-50.

59. Arshad A, Borhany M, Anwar N, et al. Prevalence of transfusion transmissible infections in blood donors of Pakistan. *BMC Hematology* 2016; **16 (1) (no pagination)**(27).

60. Awan SA, Junaid A, Sheikh S. Transfusion Transmissible Infections: Maximizing Donor Surveillance. *Cureus* 2018; **10**(12): e3787.

61. Batool Z, Durrani SH, Tariq S. Association of ABO and Rh blood group types to hepatitis B, hepatitis C, HIV and syphilis infection, a five year’experience in healthy blood donors in a tertiary care hospital. *J Ayub Med Coll Abbottabad* 2017; **29**(1): 90-2.

62. Bhatti FA, Ullah Z, Salamat N, Ayub M, Ghani E. Anti-hepatits B core antigen testing, viral markers, and occult hepatitis B virus infection in Pakistani blood donors: Implications for transfusion practice. *Transfusion* 2007; **47**(1): 74-9.

63. Bhatti MM, Junaid A, Sadiq F. The Prevalence of Transfusion Transmitted Infections among Blood Donors in Pakistan: A Retrospective Study. *Oman Med J* 2022; **37**(3): e386.

64. Butt S, Ahmad M, Saeed M, Waheed U, Rasheed N. Transfusion transmissible infectious diseases trends: Resurgence of syphilis, an old foe. *Vox Sanguinis* 2018; **113 (Supplement 1)**: 211-2.

65. Ghazanfar S, Hassan S, Shahid Z, et al. Frequency of transfusion transmissible infections among blood donors of Rawalpindi District, Pakistan. *African Health Sciences* 2022; **22(3)**: 590-8.

66. Hussain A, Mumtaz H, Aslam M, Abbas Z. Seroprevalence of transfusion based transmissible infections among clinically healthy donors in the community of Multan. *Pakistan J Inf Mol Biol* 2015; **3**(2): 47-51.

67. Jamal S, Mansoor N, Ali A, Nadeem A, Aijaz J, Meraj F. Degree of blood safety of voluntary non-remunerated versus replacement blood donations: A multi-centre study of the large cohort of blood donors from two provinces of Pakistan. *Vox Sanguinis* 2023; **118**(12): 1078-85.

68. Jiskani SA, Talpur RA, Soomro QA. Syphilis and Its Seroprevalence in Blood Donors at Rural Area of Sindh. *Asian Hematology Research Journal* 2019: 1-5.

69. Mudasar M, Meraj M, Hamid M, Khaskhili MS, Akhter N, Javed S. An epidemiological study for seroprevalence of transfusion transmissible infections among blood donors in Faisalabad, Pakistan. *Rawal Medical Journal* 2020; **45(3)**: 531-3.

70. Nawaz Z, Rasool MH, Siddique AB, et al. Prevalence and risk factors of Syphilis among blood donors of Punjab, Pakistan. *Tropical biomedicine* 2021; **38(1)**: 106-10.

71. Naz A, Mukry SN, Naseer I, Shamsi TS. Evaluation of efficacy of serological methods for detection of HCV infection in blood donors: A single centre experience. *Pakistan journal of medical sciences* 2018; **34**(5): 1204-8.

72. Nazir S, Pracha HS, Khan A, et al. Prevalence of syphilis in Pakistani blood donors. *Advancements in life sciences* 2013; **1**(1).

73. Niazi SK, Bhatti FA, Salamat N, Ghani E, Tayyab M. Impact of nucleic acid amplification test on screening of blood donors in Northern Pakistan. *Transfusion* 2015; **55**(7): 1803-11.

74. Noorulamin M, Babur A, Salman S, Faheem R, Waheed U, Hassan A. Prevalence of transfusion transmissible infections in blood donors in a tertiary level cardiac hospital in Pakistan: A 02 year experience. *Vox Sanguinis* 2016; **111 (Supplement 1)**: 201.

75. Rahat A, Kamani L. High Sero-Prevalence of Hepatitis E Virus among Healthy Asymptomatic Blood Donors in Pakistan: Can the Risk of Transfusion Dependent Transmission Be Ignored? *United European Gastroenterology Journal* 2022; **10(Supplement 8)**: 924.

76. Rauf R, Cheema A. Potential risk of transfusion-transmissible infections among blood donors in district Faisalabad of Pakistan. *Clinical Medicine* 2019; **19**(Suppl 3): s27-s.

77. Saba N, Waheed U, Nasir J, et al. Epidemiology and trends of transfusion transmissible infections among voluntary and replacement blood donors at a regional blood centre in Pakistan. *Vox Sanguinis* 2021; **116(SUPPL 1)**: 53-4.

78. Sabir N, Ghafoor T, Fatima S, Lodhi R, Mehmood A, Zaman G. Prevalence and Association of Transfusion-Transmissible Infections with Age of Blood Donors: A Regional Transfusion Centre Study in Northern Pakistan. *Journal of the College of Physicians and Surgeons Pakistan* 2023; **33**(9): 978-82.

79. Saeed M, Hussain S, Rasheed F, Ahmad M, Arif M, Rahmani MTH. Silent killers: Transfusion transmissible infections-tti, among asymptomatic population of Pakistan. *Journal of the Pakistan Medical Association* 2017; **67**(3): 369-74.

80. Shah H, Rahman ZU, Khan M, Zaman F, Badshah S. The Prevalence of Blood Borne Diseases in Blood Donors of Pesh-awar, Khyber Pakhtunkhwa, Pakistan. *Recent Advances in Anti-Infective Drug Discovery* 2023; **18**(3): 215-20.

81. Shah H, Ur Rahman Z, Khan M, Zaman F, Badshah S. The Prevalence of Blood Borne Diseases in Blood Donors of Peshawar, Khyber Pakhtunkhwa, Pakistan. *Recent Adv Antiinfect Drug Discov* 2023; **18**(3): 215-20.

82. Siddiqui F, Siddiqui F, Siddiqui N. Prevalence of transfusion-transmissible infections among voluntary blood donors in tertiary health-care facility in Islamabad. *Pakistan J Clin Trials* 2019; **9**(06): 1.000383.

83. Sultan S, Nasir MI, Rafiq S, Baig MA, Akbani S, Irfan SM. Multiplex real-time RT-PCR assay for transfusion transmitted viruses in sero-negative allogeneic blood donors: An experience from Southern Pakistan. *Malaysian Journal of Pathology* 2017; **39**(2): 149-54.

84. Zafar M, Saeed M, Ahmad M, Rasheed F, Waheed U, Arshad M. Wastage of blood & blood products: Where we are and where we should? *Vox Sanguinis* 2020; **115(SUPPL 1)**: 97.

85. Zameer M, Shahzad F, Khan FS, Ali H, Saeed U, Farooq M. TRANSFUSION TRANSMISSIBLE INFECTIONS AMONG HEALTHY BLOOD DONORS AT BLOOD BANK FROM CHILDREN'S HOSPITAL & INSTITUTE OF CHILD HEALTH LAHORE. *Pakistan Armed Forces Medical Journal* 2017; **67**(1): 131-6.

86. Aabdien M, Selim N, Himatt S, et al. Prevalence and trends of transfusion transmissible infections among blood donors in the State of Qatar, 2013-2017. *BMC Infectious Diseases* 2020; **20(1) (no pagination)**.

87. Nur YA, Groen J, Elmi AM, Ott A, Osterhaus AD. Prevalence of serum antibodies against bloodborne and sexually transmitted agents in selected groups in Somalia. *Epidemiology and infection* 2000; **124**(1): 137-41.

88. Ahmed EB, Essa AA, Almugadam BS, Ahmed QM, Hussein MM. Transfusion transmitted infections among male blood donors of White Nile State, Sudan: Screening of the current seroprevalence and distribution. *BMC research notes* 2020; **13(1)**: 549.

89. Mohammed B, Badneen M, Gibreel M, Othman S. Prevalence of transfusion-transmissible infections among blood donors in Port Sudan. *The Egyptian Journal of Haematology* 2019; **44**(1): 72-6.

90. Ben Jemia R, Gouider E. Seroprevalency of transfusion-transmitted infections in first-time volunteer and replacement donors in Tunisia. *Transfusion Clinique et Biologique* 2014; **21**(6): 303-8.

91. Raouf M, Al Amiri A, Gammon R. Demographic profile of blood donors and its influence on the safety of blood transfusion in the United Arab Emirates. *Transfusion* 2010; **2)**: 205A.

92. Alharazi T, Alzubiery TK, Alcantara JC, et al. Prevalence of Transfusion-Transmitted Infections (HCV, HIV, Syphilis and Malaria) in Blood Donors: A Large-Scale Cross-Sectional Study. *Pathogens* 2022; **11**(7).

93. Ghaleb YA, Alshahari A, Serouri AA, Alqupatii M, Al-Dubaeey M. Transfusion transmitted infection among blood donors at national blood transfusion and researcher center, Sana'a City-Yemen, 2018. *Vox Sanguinis* 2019; **114 (Supplement 1)**: 142.

94. Saghir SAM, Al-Hassan FM, Alsalahi OSA, Alhariry AEAA, Baqir HS. Frequencies of HBV, HCV, HIV, and syphilis markers among blood donors: A hospital-based study in Hodeidah, Yemen. *Tropical Journal of Pharmaceutical Research* 2012; **11**(1): 132-6.

95. Talbi F, Djameli D. Seroprevalence of Hepatitis B and C Viruses, Hiv-1/2 and Syphilis among Blood Donors in the Centre Region of Algeria. *HemaSphere* 2022; **6(Supplement 3)**: 3942-3.

96. Ahmed R, El Zayadi A, Ibrahim E, et al. Anti-HBC screening in Egyptian blood donors reduces the risk of hepatitis B virus transmission. *Vox Sanguinis* 2009; **1)**: 78.

97. Hussein E. Blood donor recruitment strategies and their impact on blood safety in Egypt. *Transfusion and Apheresis Science* 2014; **50**(1): 63-7.

98. Anwar F, Abdelaal MA, A.A AL, et al. Family replacement donors: Are they a threat to blood safety? Results from a hospital-based donor center in Saudi Arabia. *Transfusion* 2014; **2)**: 105A.

99. Al-Harthy K, Hamdalla J, Ashraf T, Alhashami S. Post donation counseling and its impact on community awareness about transfusion transsimeted infections (tti): Experience and role of department of blood banks services (DBBs), sultanate of Oman. *Vox Sanguinis* 2016; **111 (Supplement 1)**: 181-2.

100. Hafeez R, Waheed U, Kayani IS, Maqbool A, Zaheer HA. Analysis of blood donor deferral patterns in Islamabad, Pakistan. *Vox Sanguinis* 2016; **111 (Supplement 1)**: 116.

101. Kamran M, Mahmood RT, Khan MA, Mehmood A, Nisar L, Asad MJ. Prevalence of transfusion transmitted infections among blood donors; A prospective study. *AJPCT* 2014; **2**(4): 540-43.

102. Rasheed F, Saeed M, Waheed U, Ahmad M, Arshad M. Pre donation deferral pattern: A single center experience from pakistan. *Vox Sanguinis* 2020; **115(SUPPL 1)**: 120.

103. Soomro AA, Jamro B, Abro AK. Transfusion Transmitted Infections (TTIS) among blood donors of Sukkur. *Medical Forum Monthly* 2012; **23**(5): 45-7.

104. Wazeer A, Waheed U, Saba N, Arshad M, Zaheer H. Epidemiology of syphilis in blood donors in Pakistan. *Vox Sanguinis* 2020; **115(SUPPL 1)**: 217.

105. Zaffar G, Ali R, Ayyub M, Ahmed A, Ali A, Khan F. Frequency and trends of infectious pathogen in blood donors at a tertiary care hospital in Pakistan. *Hepatology International* 2013; **1)**: S346.

106. Sayedahmed A, Ebrahim A, Dirdiri S, Salah A, Ahmed K, Hamad S. Sero-prevalence of transfusion-transmittable infections among blood donors in Sudan: A national study. *British Journal of Haematology* 2023; **201(Supplement 1)**: 101-2.

107. Yousif DE. Seroprevalence of undiagnosed transfusion transmissible infections (HIV, hepatitis B and C and syphilis) in donated blood in National Blood Banks, Khartoum, Sudan. *British Journal of Haematology* 2015; **1)**: 90.

108. Todd CS, Ahmadzai M, Atiqzai F, et al. Seroprevalence and correlates of HIV, syphilis, and hepatitis B and C virus among intrapartum patients in Kabul, Afghanistan. *BMC Infectious Diseases* 2008; **8**: 119.

109. Yousif AA, Wallace MR, Baig BH, Rajab KE. Prenatal serologic screening in Bahrain. *Scandinavian Journal of Infectious Diseases* 1991; **23**(6): 781-3.

110. Attary JS, Daneshjou K, Hantooshzadeh S. Screening of syphilis in pregnant women reffered to Valiasr Hospital, Emam Khomeini Hospital. *Iranian Journal of Pediatrics* 2014; **24**(S2): S38.

111. Hassan JS. An evaluation of syphilis disease in pregnant women of Abu-grab Provence, Iraq. *Canadian Journal of Pure and Applied Sciences* 2015; **9**(2): 3379-81.

112. Lumbiganon P, Piaggio G, Villar J, et al. The epidemiology of syphilis in pregnancy. *International Journal of STD and AIDS* 2002; **13**(7): 486-94.

113. Shakoor Z. Antenatal screening for syphilis at a tertiary care hospital in Riyadh. *Annals of Saudi Medicine* 2004; **24**(4): 262-4.

114. Shah S. Do we need universal screening for syphilis among antenatal clinic attendees in Pakistan? International AIDS Society; 2002.

115. Shah SA, Kristensen S, Memon MA, et al. Prevalence of syphilis among antenatal clinic attendees in Karachi: Imperative to begin universal screening in Pakistan. *Journal of the Pakistan Medical Association* 2011; **61**(10): 993-7.

116. Abdalla E, Ekanem E, Said D, Arube P, Gboun M, Mohammed F. The need for a comprehensive response to HIV/AIDS in north-western Somalia: Evidence from a seroprevalence survey. *Eastern Mediterranean Health Journal* 2010; **16**(2): 141-5.

117. Jama H, Hederstedt B, Osman S, Omar K, Isse A, Bygdeman S. Syphilis in women of reproductive age in Mogadishu, Somalia: Serological survey. *Genitourinary Medicine* 1987; **63**(5): 326-8.

118. Lodiongo DK, Bior BK, Dumo GW, et al. Field evaluation of SD BIOLINE HIV/Syphilis Duo assay among pregnant women attending routine antenatal care in Juba, South Sudan. *PLoS ONE* 2018; **13 (10) (no pagination)**(e0205383).

119. Nagi AM, Allah HAW, Khalil OM. Seroprevalence of syphilis among pregnant women in the Tri-capital, Khartoum, Sudan. *Res J Med Sc* 2008; **3**: 48-52.

120. Ortashi OM, El Khidir I, Herieka E. Prevalence of HIV, syphilis, Chlamydia trachomatis, Neisseria gonorrhoea, Trichomonas vaginalis and candidiasis among pregnant women attending an antenatal clinic in Khartoum, Sudan. *Journal of Obstetrics and Gynaecology* 2004; **24**(5): 513-5.

121. Znazen A, Zribi N, Maazoun L, Khrouf S, Hammami A. Epidemiological features of sexually transmitted infections among women in Tunisia: High prevalence of chlamydia trachomatis among women requesting abortion. *Sexually Transmitted Infections* 2013; **89**(1): 56.

122. Ayachi F, Kechrid A, Lagha N, Ben Hamida A, Amamou H, Ben Mahmoud R. Seroprevalence rate of syphilis in 3 groups of sexually active tunisian women. [French]. *Medecine et Maladies Infectieuses* 1997; **27**(11): 913-4.

123. AYAT EJ, Zare A. Antenatal screening for syphilis in Yazd. 2006.

124. Josheghani SB, Moniri R, Taheri FB, Sadat S, Heidarzadeh Z. The prevalence of serum antibodies in TORCH infections during the first trimester of pregnancy in Kashan, Iran. *Iranian Journal of Neonatology* 2015; **6**(1): 8-12.

125. Motamedifar M, Hassanzadeh P, Taghinia MA, Hassazadeh Y. DETECTION OF SYPHILIS BY SEROLOGIC TESTS IN PREGNANT IRANIAN WOMEN, SHIRAZ, IRAN (SHORT COMMUNICATIONORT). 2013.

126. Emmanuel SK, Lado M, Amwayi S, Abade AM, Oundo JO, Ongus JR. Syphilis among pregnant women in Juba, Southern Sudan. *East African medical journal* 2010; **87**(5): 192-8.

127. Aidaoui M, Bouzbid S, Laouar M. Seroprevalence of HIV infection in pregnant women in the Annaba region (Algeria). [French]. *Revue d'Epidemiologie et de Sante Publique* 2008; **56**(4): 261-6.

128. Chaudry AE, Chaudhri R, Kayani A, et al. Acceptability and feasibility of screening pregnant women for sexually transmitted infections in Rawalpindi, Pakistan. *International Journal of STD and AIDS* 2021; **32(10)**: 940-5.

129. McCauley M, Madaj B, White SA, et al. Burden of physical, psychological and social ill-health during and after pregnancy among women in India, Pakistan, Kenya and Malawi. *BMJ global health* 2018; **3**(3): e000625.

130. Abdelrahim NA, Ahmed HI, Fadl-Elmula IM, Bayoumi MA, Homeida MM. Sexually transmitted infections other than HIV/AIDS among women of low socio-economic class attending antenatal clinics in Khartoum, Sudan. *International Journal of STD and AIDS* 2017; **28**(8): 781-7.

131. Elkheir SM, Babiker ZO, Elamin SK, et al. Seroprevalence of maternal HIV, hepatitis B, and syphilis in a major maternity hospital in North Kordofan, Sudan. *International journal of STD & AIDS* 2018: 956462418784687.

132. Sudan National HIV/AIDS Prevention and Control Programme. 2010 ANC HIV Sentinel Sero-Survey: Technical Report Sudan 2014.

133. Sudan National HIV/AIDS Prevention and Control Programme. 2009 ANC HIV Sentinel Sero-Survey: Technical Report. Sudan 2010.

134. Jabara H, Soltani MS, Bchir A, Ouni M, Jeddi M. Evaluation of the systematic serodiagnosis of syphilis in the area of Monastir (Tunisia). [French]. *Medecine et Maladies Infectieuses* 1993; **23**(5): 383-4.

135. WHO Eastern Mediterranean Region. HIV surveillance systems: Regional update 2011, 2011.

136. Safir N, Adnaoui M, Halim A, et al. Prevalence of HIV and other sexually transmitted infections in pregnant women in routine antenatal care. *Clinical Chemistry and Laboratory Medicine* 2012; **50 (4)**: A120.

137. Gul F, Faiz NR, Raziq F, et al. Frequency of vaginal discharge and its association with various sexually transmitted diseases in women attending antenatal clinic. *Journal of Postgraduate Medical Institute* 2005; **19**(1): 86-91.

138. Orang M, Asmar M. Seroreversion of serological tests for syphilis in the disabled community in Mazandaran province of Iran. 2002.

139. Mahafzah AM, Al-Ramahi MQ, Asagd AM, El-Khateeb MS. Prevalence of sexually transmitted infections among sexually active Jordanian females. *Sexually Transmitted Diseases* 2008; **35**(6): 607-10.

140. Zaki SA, Naous J, Ghanem A, et al. Prevalence of STIs, sexual practices and substance use among 2083 sexually active unmarried women in Lebanon. *Scientific reports* 2021; **11(1)**: 9855.

141. Deeb ME, Awwad J, Yeretzian JS, Kaspar HG. Prevalence of reproductive tract infections, genital prolapse, and obesity in a rural community in Lebanon. *Bulletin of the World Health Organization* 2003; **81**(9): 639-45.

142. Khallouk JL, Zeggwagh A, El Bakkouri J, Takourt B, Farouqi B, Fellah H. Syphilis prevalence of patients in IBN Rochd teaching hospital of casablanca from 2008 to 2011. *Clinical Chemistry and Laboratory Medicine* 2012; **50 (4)**: A124.

143. Mir AM, Wajid A, Reichenbach L, Khan M. STI prevalence and associated factors among urban men in Pakistan. *Sexually Transmitted Infections* 2009; **85**(3): 199-200.

144. Nasrallah GK, Al-Buainain R, Younes N, et al. Screening and diagnostic testing protocols for HIV and Syphilis infections in health care setting in Qatar: Evaluation and recommendations. *PloS one* 2023; **18**(2): e0278079.

145. Ismail SO, Ahmed HJ, Jama MA, et al. Syphilis, gonorrhoea and genital chlamydial infection in a Somali village. *Genitourin Med* 1990; **66**(2): 70-5.

146. Kafi SK, Mohamed AO, Musa HA. Prevalence of sexually transmitted diseases (STD) among women in a suburban Sudanese community. *Upsala journal of medical sciences* 2000; **105**(3): 249-53.

147. Zribi M, Ben Mansour K, Abid F, Masmoudi A, Fendri C. Syndromic approach to sexually transmitted infections in Tunisian women: Bacteriological validation. *International Journal of STD and AIDS* 2008; **19**(2): 112-4.

148. Nsanze H, Lestringant GG, Ameen AM, Lambert JM, Galadari I, Usmani MA. Serologic tests for treponematoses in the United Arab Emirates. *International Journal of Dermatology* 1996; **35**(11): 800-1.

149. Nassery T, Seigneurin JM. A serologio study in Afghanistan (rickettsioses, brucelloses, typhoid fever, syphilis). [French]. *Bulletin de la Societe de pathologie exotique et de ses filiales* 1971; **64**(6): 827-32.

150. Cirera P, Lefevre-Witier P. Serology of syphilis in Tassili n'Ajjer (Central Sahara). [French]. *Bulletin de la Societe de pathologie exotique et de ses filiales* 1967; **60**(1): 33-43.

151. Rodier GR, Morand JJ, Olson JS, Watts DM, Said S. HIV infection among secondary school students in Djibouti, horn of Africa: knowledge, exposure and prevalence. *East African medical journal* 1993; **70**(7): 414-7.

152. El-Sayed NM, Gomatos PJ, Rodier GR, et al. Seroprevalence survey of Egyptian tourism workers for hepatitis B virus, hepatitis C virus, human immunodeficiency virus, and Treponema pallidum infections: Association of hepatitis C virus infections with specific regions of Egypt. *American Journal of Tropical Medicine and Hygiene* 1996; **55**(2): 179-84.

153. Van Peenen PFO, Reid TP. A SEROLOGICAL and STOOL SURVEY of BEDOUIN TRIBESMEN in THE WESTERN DESERT of EGYPT. *Trop* 1963; **Geogr. Med. 15**(3): 243-8.

154. Hodgson R, Stewart IS. Serological investigation for treponemal infection of artisan recruits in South Persia. *British Journal of Venereal Diseases* 1951; **27**(4): 188-9.

155. El Ghoroury AA. The syphilis problem in Asir province, Saudi Arabia. *Bulletin of the World Health Organization* 1954; **10**(4): 691-702.

156. Zahraoui-Mehadji M, Baakrim MZ, Laraqui S, et al. [Infectious risks associated with blood exposure for traditional barbers and their customers in Morocco]. *Sante* 2004; **14**(4): 211-6.

157. Hyder O, Ijaz M, Arshad MA, Zahira T. Frequency of VDRL seropositivity among clinically healthy adult men from districts of Punjab province in Pakistan. *Sexually Transmitted Infections* 2010; **86**(7): 513.

158. Khan E, Memon BI, Ayaz A, et al. Trend of syphilis in Pakistan, 1991-2008. *Indian Journal of Medical Microbiology* 2010; **28**(3): 263-4.

159. Khan EA, Khokhar N, Malik GJ. Seroprevalence of syphilis in asymptomatic adults seeking employment abroad. *Rawal Med J* 2004; **29**(2): 65-7.

160. Ahmed SA, Sanyal RK, Ahmed MG. Prevalence of syphilis in South Sudan. *The Journal of communicable diseases* 1985; **17**(3): 251.

161. Younis N, Khattab H, Zurayk H, el-Mouelhy M, Amin MF, Farag AM. A community study of gynecological and related morbidities in rural Egypt. *Studies in family planning* 1993; **24**(3): 175-86.

162. Memish ZA, Almasri M, Chentoufi AA, et al. Seroprevalence of herpes simplex virus type 1 and type 2 and coinfection with HIV and syphilis: The first national seroprevalence survey in Saudi Arabia. *Sexually Transmitted Diseases* 2015; **42**(9): 526-32.

163. Filemban SM, Yasein YA, Abdalla MHH, Al-Hakeem R, Al-Tawfiq JA, Memish ZA. Prevalence and behavioral risk factors for STIs/HIV among attendees of the Ministry of Health hospitals in Saudi Arabia. *Journal of Infection in Developing Countries* 2015; **9**(4): 402-8.

164. Kaiser R, Kebdamo T, Lane J, Kessia G, Handzel T, Downing R. HIV/STI prevalence and risk factor surveys in Yei and Rumbek, South Sudan, 2002/2003. Proceedings of the XV International Conference on AIDS; 2004; 2004. p. 11-6.

165. Sube KL, Seriano O, Lako J, et al. Prevalence of HBV, HCV, HIV and syphilis infections among secondary school students in Juba, South Sudan. *American Journal of Tropical Medicine and Hygiene* 2020; **103(5 SUPPL)**: 9-10.

166. Todd CS, Nasir A, Mansoor GF, et al. Cross-sectional assessment of prevalence and correlates of blood-borne and sexually-transmitted infections among Afghan National Army recruits. *BMC Infectious Diseases* 2012; **12 (no pagination)**(196).

167. Yousif A, Wallace M, Baig B. The seroprevalence of syphilis, toxoplasmosis and hepatitis B in patients in Bahrain infected with human immunodeficiency virus. *Transactions of the Royal Society of Tropical Medicine and Hygiene* 1994; **88**(1): 60.

168. Hashemi-Shahri SM, Sharifi-Mood B, Kouhpayeh HR, Moazen J, Farrokhian M, Salehi M. Sexually Transmitted Infections Among Hospitalized Patients With Human Immunodeficiency Virus Infection and Acquired Immune Deficiency Syndrome (HIV/AIDS) in Zahedan, Southeastern Iran. *International journal of high risk behaviors & addiction* 2016; **5**(3): e28028.

169. Vahdani P, Hosseini-Moghaddam SM, Gachkar L, Sharafi K. Prevalence of hepatitis B, hepatitis C, human immunodeficiency virus, and syphilis among street children residing in southern Tehran, Iran. *Archives of Iranian Medicine* 2006; **9**(2): 153-5.

170. Yanni EA, Naoum M, Odeh N, Han P, Coleman M, Burke H. The health profile and chronic diseases comorbidities of US-bound Iraqi refugees screened by the International Organization for Migration in Jordan: 2007-2009. *Journal of immigrant and minority health / Center for Minority Public Health* 2013; **15**(1): 1-9.

171. Chowdhury MNH, Jamjoom GA, Pareek SS, Mahgoub ES. Incidence of treponemal infection. In different clinics at King Abdul Aziz Teaching Hospital Riyadh, Saudi Arabia. *Saudi Medical Journal* 1982; **3**(1): 31-4.

172. Hamdi SA, Ibrahim MA. Sexually Transmitted Diseases in Domestic Expatriate Workers in Jeddah, Saudi Arabia. *Annals of Saudi Medicine* 1997; **17**(1): 29-31.

173. Bourouache M, Mimouni R, Nejmeddine M, et al. The prevalence of syphilis in HIV-seropositive patients: A retrospective study at the regional hospital in Agadir, Morocco. *Pan African Medical Journal* 2019; **33 (no pagination)**(252).

174. El Ghrari K, Terrab Z, Benchikhi H, Lakhdar H, Jroundi I, Bennani M. Prevalence of syphilis and HIV infection in female prisoners in Morocco. [French]. *Eastern Mediterranean Health Journal* 2007; **13**(4): 774-9.

175. Altaf A, Shah SA, Zaidi NA, Memon A, Nadeem ur R, Wray N. High risk behaviors of injection drug users registered with harm reduction programme in Karachi, Pakistan. *Harm Reduction Journal* 2007; **4 (no pagination)**(7).

176. Baqi S, Nabi N, Hasan SN, et al. HIV antibody seroprevalence and associated risk factors in sex workers, drug users, and prisoners in Sindh, Pakistan. *Journal of acquired immune deficiency syndromes and human retrovirology : official publication of the International Retrovirology Association* 1998; **18**(1): 73-9.

177. Faisel A, Cleland J. Migrant men: A priority for HIV control in Pakistan? *Sexually Transmitted Infections* 2006; **82**(4): 307-10.

178. Laghari AH, Sultana V, Samoo AH, Makhija P, Ara J. Prevalence and associated risk factors for syphilis in women with recurrent miscarriages. *Pakistan journal of medical sciences* 2014; **30**(2): 295.

179. National AIDS Control Program. National Study of Reproductive Tract and Sexually Transmitted Infections: Survey of High Risk Groups in Lahore and Karachi. Pakistan 2005.

180. Kazi AM, Shah SA, Jenkins CA, Shepherd BE, Vermund SH. Risk factors and prevalence of tuberculosis, human immunodeficiency virus, syphilis, hepatitis B virus, and hepatitis C virus among prisoners in Pakistan. *International Journal of Infectious Diseases* 2010; **14**(SUPPL. 3): e60-e6.

181. Platt L, Vickerman P, Collumbien M, et al. Prevalence of HIV, HCV and sexually transmitted infections among injecting drug users in Rawalpindi and Abbottabad, Pakistan: Evidence for an emerging injection-related HIV epidemic. *Sexually Transmitted Infections* 2009; **85**(SUPPL. 2): ii17-ii22.

182. International Organization for Migration (IOM). Integrated HIV and Sexually Transmitted Infections (STIs) Bio-Behavioral Survey (IBBS) among Key Populations in Somalia. Somalia, 2017.

183. International Organization for Migration (IOM). Integrated HIV and Sexually Transmitted Infections (STIs) Bio-Behavioral Survey (IBBS) among Key Populations in Somaliland. Somaliland, 2017.

184. Watts DM, Corwin AL, Omar MA, Hyams KC. Low risk of sexual transmission of hepatitis C virus in Somalia. *Transactions of the Royal Society of Tropical Medicine and Hygiene* 1994; **88**(1): 55-6.

185. Kaiser R, Kedamo T, Lane J, et al. HIV, syphilis, herpes simplex virus 2, and behavioral surveillance among conflict-affected populations in Yei and Rumbek, southern Sudan. *Aids* 2006; **20**(6): 942-4.

186. Badie BM, Yavari Z, Esmaeeli S, et al. Prevalence survey of infection with Treponema pallidum among HIV-positive patients in Tehran. *Asian Pacific Journal of Tropical Biomedicine* 2013; **3**(4): 334-6.

187. Ghassabi F, Malekzadegan Y, Ebrahim-Saraie HS, et al. Gonorrhea and syphilis co-infection and related risk factors in HIV patients from Shiraz, South of Iran. *Caspian Journal of Internal Medicine* 2018; **9**(4): 397-402.

188. Mohamadi H, Esfahani A. Prevalence of syphilis and Chlamydia trachomitis in soldiers. *Journal Mil Med* 2009; **11**(2): 109-14.

189. Hajiabdolbaghi M, Razani N, Karami N, et al. Insights from a survey of sexual behavior among a group of at-risk women in Tehran, Iran, 2006. *AIDS Education and Prevention* 2007; **19**(6): 519-30.

190. Jahanbakhsh F, Bagheri Amiri F, Sedaghat A, Fahimfar N, Mostafavi E. Prevalence of HAV Ab, HEV (IgG), HSV2 IgG, and Syphilis Among Sheltered Homeless Adults in Tehran, 2012. *International journal of health policy and management* 2017; **7**(3): 225-30.

191. Nokhodian Z, Yazdani MR, Yaran M, et al. Prevalence and risk factors of HIV, syphilis, hepatitis B and C among female prisoners in Isfahan, Iran. *Hepatitis Monthly* 2012; **12**(7): 92-7.

192. Vahdani P, Hosseini-Moghaddam SM, Family A, Moheb-Dezfouli R. Prevalence of HBV, HCV, HIV and syphilis among homeless subjects older than fifteen years in Tehran. *Arch Iran Med* 2009; **12**(5): 483-7.

193. Al-Mughales JA. Co-infection assessment in HBV, HCV, and HIV patients in Western Saudi Arabia. *Journal of Medical Virology* 2016.

194. National AIDS Control Program-Ministry of Public Health. Integrated Biological & Behavioral surveillance (IBBS) in Selected Cities of Afghanistan: Findings of 2012 IBBS Survey and Comparison to 2009 IBBS Survey. Afghanistan 2012.

195. National AIDS Control Program-Ministry of Public Heath. Integrated Behavioral & Biological Surveillance (IBBS) in Afghanistan: Year 1 report. Afghanistan 2010.

196. Nasir A. Prevalence of HIV, viral hepatitis, syphilis and risk behaviors among injection drug users in Hirat, Afghanistan. International AIDS Society; 2008.

197. Ruisenor-Escudero H, Wirtz AL, Berry M, et al. Risky behavior and correlates of HIV and Hepatitis C Virus infection among people who inject drugs in three cities in Afghanistan. *Drug and Alcohol Dependence* 2014; **143**(1): 127-33.

198. Todd CS, Nasir A, Raza Stanekzai M, et al. Prevalence and correlates of syphilis and condom use among male injection drug users in four Afghan cities. *Sexually Transmitted Diseases* 2010; **37**(11): 719-25.

199. Todd CS, Nasir A, Stanekzai MR, et al. Prevalence and correlates of HIV, syphilis, and hepatitis B and C infection and harm reduction program use among male injecting drug users in Kabul, Afghanistan: A cross-sectional assessment. *Harm Reduction Journal* 2011; **8 (no pagination)**(22).

200. Todd CS. Seroprevalence of sexually and blood-borne transmitted infections among Afghan National Security Forces recruits. International AIDS Conference, 2012; 2012.

201. Ministry of Health. Integrated Biological and Behavioral Surveillance Survey (IBBSS) among Female Sex Workers and Long-Distance Truck Drivers in Four Quartiers in Djibouti: Survey Report. Djibouti 2014.

202. Zaki SA. Sexual practices, contraception methods, and self-reported urogenital symptoms among 116 refugee women in Beirut Lebanon. International AIDS Society, 2020; 2020.

203. Rehan N, Bokhari A, Nizamani NM, et al. National study of reproductive tract infections among high risk groups of Lahore and Karachi. *Journal of the College of Physicians and Surgeons Pakistan* 2009; **19**(4): 228-31.

204. Rehman Khan A, Ahmad B, Waheed U, Hafeez R, Wazeer A, Abbas Zaheer H. Evaluation of rapid screening tests for syphilis in Islamabad, Pakistan. *Vox Sanguinis* 2017; **112 (Supplement 1)**: 182.

205. Sahiner F, Idiris MH, Hosbul T, et al. HIV Seroprevalence in Mogadishu, Somalia: a Retrospective Study between 2015 and 2019. *Clinical Laboratory* 2022; **68**(7).

206. Teyeb H. Epidemiological profile of infectious diseases in the region of Ben Guerdane (Southern Tunisia). *Biochimica Clinica* 2013; **1)**: S153.

207. Eihab AAA, Mousa A, Adam DA, Bakri YMN, Ra'ed A, Ali E. Prevalence of Syphilis and Human Immunodeficiency Virus in expatriates in Sharjah, United Arab Emirates. *Trop Biomed* 2016; **33**(4): 613-8.

208. Beheshti S, Salehi L, Ziadlou S. Prevalence of Commonly Encountered Sexually Transmitted Infections Among Imprisoned Incarcerated Women. *Women’s Health Bulletin* 2014; **1**(3): 1-5.

209. Faramarzi H. HIV/AIDS and co-morbid diseases among people visiting VCT center in Shiraz, South Iran: a time-series analysis investigation. Internetional AIDS Society; 2010.

210. Ledward RS. Infertility in Saudi Arabia: initial experience in a new gynaecological unit. *Trop Doct* 1980; **10**(3): 117-9.

211. Belglaiaa E, Souho T, Badaoui L, et al. Awareness of cervical cancer among women attending an HIV treatment centre: A cross-sectional study from Morocco. *BMJ Open* 2018; **8 (8) (no pagination)**(e020343).

212. Al Soub H, Al-Khal AL, Al Maslamani M, Dousa K, Ahmed A, Fabella A. Epidemiology and the Changing Face of HIV Infection in Qatar. *Infectious Diseases in Clinical Practice* 2018; **26**(4): 220-3.

213. Kazerooni PA, Motazedian N, Motamedifar M, et al. The prevalence of human immunodeficiency virus and sexually transmitted infections among female sex workers in Shiraz, South of Iran: By respondent-driven sampling. *International Journal of STD and AIDS* 2014; **25**(2): 155-61.

214. Shahesmaeili A, Karamouzian M, Shokoohi M, et al. Symptom-Based Versus Laboratory-Based Diagnosis of Five Sexually Transmitted Infections in Female Sex Workers in Iran. *AIDS and behavior* 2018.

215. Hancali Sr A. Prevalence of stis among female sex workers in agadir in the south of Morocco. *Sexually Transmitted Diseases* 2014; **1)**: S145-S6.

216. Ministry of Health-Morocco, The Joint United Nations Programme on HIV/AIDS (UNAIDS), The Global Fund. HIV Integrated Behavioral and Biological Surveillance Surveys: Female Sex Workers in Agadir, Fes, Rabat and Tanger. Morocco, 2012.

217. Johnston LG, Alami K, Rhilani MHE, et al. HIV, syphilis and sexual risk behaviours among men who have sex with men in Agadir and Marrakesh, Morocco. *Sexually Transmitted Infections* 2013; **89**(SUPPL. 3): iii45-iii8.

218. Oukouchoud H, Ouanaim C, Bellaji B, et al. Sero-prevalence of syphilis among female sex workers in morocco. *Sexually Transmitted Infections* 2017; **93 (Supplement 2)**: A158-A9.

219. Hawkes S, Collumbien M, Platt L, et al. HIV and other sexually transmitted infections among men, transgenders and women selling sex in two cities in Pakistan: A cross-sectional prevalence survey. *Sexually Transmitted Infections* 2009; **85**(SUPPL. 2): ii8-ii16.

220. Khan MS, Unemo M, Zaman S, Lundborg CS. HIV, STI prevalence and risk behaviours among women selling sex in Lahore, Pakistan. *BMC Infectious Diseases* 2011; **11 (no pagination)**(119).

221. Baqi S, Shah SA, Baig MA, Mujeeb SA, Memon A. Seroprevalence of HIV, HBV and syphilis and associated risk behaviours in male transvestites (Hijras) in Karaehi, Pakistan. *International Journal of STD and AIDS* 1999; **10**(5): 300-4.

222. Shah A, Memon M, Soomro S, Kazi N, Kristensen S. Seroprevalence of HIV, syphilis, hepatitis B and hepatitis C among female commercial sex workers in Hyderabad, Pakistan. Int AIDS Conf; 2004; 2004. p. C12368.

223. Bchir A, Jemni L, Saadi M, Milovanovic A, Brahim H, Catalan F. Markers of sexually transmitted diseases in prostitutes in central Tunisia. *Genitourin Med* 1988; **64**(6): 396-7.

224. Corwin AL, Olson JG, Omar MA, Razaki A, Watts DM. HIV-1 in Somalia: prevalence and knowledge among prostitutes. *Aids* 1991; **5**(7): 902-4.

225. International Organization for Migration (IOM). Integrated Biological and Behavioural Surveillance Survey among Vulnerable Women in Hargeisa, Somaliland. Somaliland, 2017.

226. Jama Ahmed H, Omar K, Adan SY, Guled AM, Grillner L, Bygdeman S. Syphilis and human immunodeficiency virus seroconversion during a 6-month follow-up of female prostitutes in Mogadishu, Somalia. *International Journal of STD and AIDS* 1991; **2**(2): 119-23.

227. Okiria AG, Achut V, McKeever E, et al. High HIV and syphilis prevalence among female sex workers and sexually exploited adolescents in Nimule town at the border of South Sudan and Uganda. *PLoS ONE* 2023; **18(1 January) (no pagination)**.

228. Government of the Republic of South Sudan-Ministry of Health. A Biobehavioral HIV Survey of Female Sex Workers in South Sudan. South Sudan, 2016.

229. Government of the Republic of South Sudan-Ministry of Health. A bio-behavioral HIV survey of female sex workers in Wau, South Sudan: The Eagle II Survey. South Sudan, 2020.

230. Government of the Republic of South Sudan-Ministry of Health. A bio-behavioral HIV survey of female sex workers in Yambio, South Sudan: The Eagle II Survey. South Sudan, 2020.

231. Bashmaq SM, Ahmadi A, Mohsenpour B, et al. Prevalence of HIV, HBV, HCV, HPV and syphilis among female sex workers in Kurdistan, west of Iran. *Caspian Journal of Internal Medicine* 2024; **15**(1): 38-45.

232. Kassaian N, Ataei B, Yaran M, Babak A, Shoaei P, Ataie M. HIV and other sexually transmitted infections in women with illegal social behavior in Isfahan, Iran. *Advanced biomedical research* 2012; **1**: 5.

233. Navadeh S, Mirzazadeh A, Mousavi L, Haghdoost A, Fahimfar N, Sedaghat A. HIV, HSV2 and Syphilis Prevalence in Female Sex Workers in Kerman, South-East Iran; Using Respondent-Driven Sampling. *Iran J Public Health* 2012; **41**(12): 60-5.

234. Assi A, Abu Zaki S, Ghosn J, et al. Prevalence of HIV and other sexually transmitted infections and their association with sexual practices and substance use among 2238 MSM in Lebanon. *Scientific reports* 2019; **9(1)**: 15142.

235. Hakim AJ, Bolo A, Werner M, et al. High HIV and syphilis prevalence among female sex workers in Juba, South Sudan. *PLoS ONE* 2020; **15(9 September) (no pagination)**.

236. Štulhofer A, Bozicevic I. HIV Bio-Behavioural Survey among FSWs in Aden, Yemen. Yemen, 2008.

237. Todd C. Prevalence of HIV, viral hepatitis, syphilis and risk behaviors among sex workers in Afghanistan. International AIDS Conference, 2012; 2008.

238. Todd CS, Nasir A, Stanekzai MR, et al. HIV, hepatitis B, and hepatitis C prevalence and associated risk behaviors among female sex workers in three Afghan cities. *Aids* 2010; **24**(SUPPL. 2): S69-S75.

239. Izadi N, Gouya MM, Akbarpour S, et al. HIV prevalence and associated factors among female sex workers in Iran: a bio-behavioral survey in 2020. *AIDS Behav* 2023; **27**(3): 909-18.

240. Khezri M, Shokoohi M, Mirzazadeh A, et al. Early sex work initiation and its association with condomless sex and sexually transmitted infections among female sex workers in Iran. *International Journal of STD and AIDS* 2020; **31(7)**: 671-9.

241. Connecting Research to Development (CRD), National AIDS Programme (NAP), International Organization for Migration (IOM). An Integrated Bio Behavioral Surveillance Study among Two Vulnerable Groups in Lebanon: Men who have Sex with Men and Commercial Sex Workers. Lebanon, 2019.

242. Bibi I, Devrajani BR, Shah SZA, Soomro MH, Jatoi MA. Frequency of syphilis in female sex workers at red light area of Hyderabad, Pakistan. *Journal of the Pakistan Medical Association* 2010; **60**(5): 353-6.

243. Javed H, Bano A, Fatima W, Khan R, Akhtar A. Sexually transmitted infections and associated risk factors among the transgender population of Pakistan. *BMC Infectious Diseases* 2023; **23**(1): 618.

244. Raza MH. HIV/AIDS and syphilis screening among high risk groups. *Journal of Rawalpindi Medical College* 2015; **19**(1): 11-4.

245. Burans JP, Fox E, Omar MA, et al. HIV infection surveillance in Mogadishu, Somalia. *East African medical journal* 1990; **67**(7): 466-72.

246. Kriitmaa K, Testa A, Osman M, et al. HIV prevalence and characteristics of sex work among female sex workers in Hargeisa, Somaliland, Somalia. *Aids* 2010; **24**(SUPPL. 2): S61-S7.

247. International Organization for Migration, World Health Organization. HIV & Syphilis Bio-Behavioural Surveillance Survey (BSS) among Female Transactional Sex Workers in Hargeisa, Somaliland. Somaliland, 2008.

248. Elhadi M, Elbadawi A, Abdelrahman S, et al. Integrated bio-behavioural HIV surveillance surveys among female sex workers in Sudan, 2011-2012. *Sexually Transmitted Infections* 2013; **89**(SUPPL. 3): iii17-iii22.

249. National AIDS Control Programme. Integrated Bio-Behavioral HIV Surveillance Survey (IBBS) among Female Sex Workers and Men who have Sex with Men in Sudan. Sudan 2012.

250. Znazen A, Frikha-Gargouri O, Berrajah L, et al. Sexually transmitted infections among female sex workers in Tunisia: High prevalence of Chlamydia trachomatis. *Sexually Transmitted Infections* 2010; **86**(7): 500-5.

251. Afsar Kazerooni P, Motazedian N, Motamedifar M, et al. The prevalence of HIV and STIs among female sex workers in Shiraz, South of Iran: By respondent driven sampling. *Sexually Transmitted Infections Conference: STI and AIDS World Congress* 2013; **89**(SUPPL. 1).

252. Mirzazadeh A, Shokoohi M, Karamouzian M, et al. Declining trends in HIV and other sexually transmitted infections among female sex workers in Iran could be attributable to reduced drug injection: a cross-sectional study. *Sex Transm Infect* 2020; **96**(1): 68-75.

253. Moayedi-Nia S, Bayat Jozani Z, Esmaeeli Djavid G, et al. HIV, HCV, HBV, HSV, and syphilis prevalence among female sex workers in Tehran, Iran, by using respondent-driven sampling. *AIDS Care - Psychological and Socio-Medical Aspects of AIDS/HIV* 2016; **28**(4): 487-90.

254. Johnston L, Bennani A, Latifi A, et al. Using respondent-driven sampling to estimate HIV and syphilis prevalence among female sex workers in agadir, fes, rabat and tangier, Morocco. *Sexually Transmitted Infections Conference: STI and AIDS World Congress* 2013; **89**(SUPPL. 1).

255. Johnston L. Vulnerability, risk and HIV, Syphilis and other infections among female sex workers in Agadir, Casablanca, Fes, Marrakesh, Rabat and Tangier, Morocco 2016. International AIDS Society; 2018.

256. Khan A. Differences in Sexual Behaviors of Male Commercial Sex Workers (MSWs) and Transgenders/Transvestites (Hijras) In Pakistan. International AIDS Society; 2006.

257. Khan AA, Rehan N, Qayyum K, Khan A. Correlates and prevalence of HIV and sexually transmitted infections among Hijras (male transgenders) in Pakistan. *International Journal of STD and AIDS* 2008; **19**(12): 817-20.

258. Narjis R, Qureshi AF. New face of female sex work in Pakistan: need for innovative interventions. *Pakistan Journal of Public Health* 2011; **1**(1): 28-35.

259. Osama M. Rate of STI among selected MSM and transgenders in 5 cities of Pakistan. *Sexually Transmitted Infections* 2017; **93 (Supplement 2)**: A18.

260. Solangon M, Kriitmaa K, Taher S, et al. Integrated biological and behavioural surveillance (IBBS) survey among men who have sex with men in South Sudan. *Journal of the International AIDS Society Conference: 22nd International AIDS Conference, AIDS* 2018; **21**(Supplement 6).

261. Tunisian National Program. Integrated Biological-Behavioral Surveillance (IBBS) Surveys among People who Inject Drugs, Men who have Sex with Men and Female Sex Workers -Tunisia. Tunisia, 2021.

262. Ghanaat J, Sadeghian A, Ghazvini K, Nassiri MR. Prevalence and risk factors for hepatitis B virus infections among STD patients in northeast region of Iran. *Medical Science Monitor* 2003; **9**(2): CR91-CR4.

263. Madani TA. Sexually transmitted infections in Saudi Arabia. *BMC infectious diseases* 2006; **6 (no pagination)**(3).

264. Pareek SS, Chowdhury MNH. Sexually transmitted diseases in Riyadh, Saudi Arabia. A study of patients attending a teaching hospital clinic. *British Journal of Venereal Diseases* 1981; **57**(5): 343-5.

265. Ryan CA, Zidouh A, Manhart LE, et al. Reproductive tract infections in primary healthcare, family planning, and dermatovenereology clinics: Evaluation of syndromic management in Morocco. *Sexually Transmitted Infections* 1998; **74**(SUPPL. 1): S95-S105.

266. Maan MA, Hussain F, Iqbal J, Akhtar SJ. Sexually transmitted infections in Pakistan. *Annals of Saudi Medicine* 2011; **31**(3): 263-9.

267. Ismail SO, Ahmed HJ, Grillner L, Hederstedt Issa BA, Bygdeman S. Sexually transmitted diseases in men in Mogadishu, Somalia. *International Journal of STD and AIDS* 1990; **1**(2): 102-6.

268. Omer EE, Ali MH, Taha OM, Ahmed MA, Abbaro SA. Sexually transmitted diseases in Sudanese males. *Tropical doctor* 1982; **12**(4 Pt 2): 208-10.

269. Omer EFEO, El-Naeem HAR, Ali MH. Micro-organisms associated with vaginal trichomoniasis among Sudanese women. *Saudi Medical Journal* 1985; **6**(2): 129-34.

270. Taha O, Ali MH, Omer E, Ahmed M, Abbaro S. Study of STDs in patients attending venereal disease clinics in Khartoum, Sudan. *Sexually Transmitted Infections* 1979; **55**(5): 313-5.

271. Shareef MA, Al Tamimi MH, Al Ammadi NY, et al. Characteristics of patients with anogenital wart: An observational and comparative study. *Bahrain Medical Bulletin* 2021; **43(3)**: 551-5.

272. Kareem HK, Hamad MM, Hasan MA, Abd alsammed MA. Study on Trichomonas vaginalis infection in women with type-2 diabetes mellitus and vaginal discharge in Thi-Qar Government. *European Journal of Molecular and Clinical Medicine* 2020; **7(8)**: 4471-8.

273. Heikel J, Sekkat S, Bouqdir F, et al. The prevalence of sexually transmitted pathogens in patients presenting to a casablanca STD clinic. *European Journal of Epidemiology* 1999; **15**(8): 711-5.

274. Alemi AA, Subramanian M. The problem of venereal diseases in Teheran: a retrospective study. *Iranian Journal of Public Health* 1976; **4**(4): 254-65.

275. Al-Mutairi B. Hepatitis B and Hepatitis C Virus Prevalence Among Sexually Transmitted Disease Patients in Farwania Region of Kuwait. *Sexually Transmitted Infections* 2013; **89**(Suppl 1): A198-A9.

276. Toudou-Daouda M, Filali-Adib A, Slassi A, Belahsen MF, Souirti Z. Limbic encephalitis: Experience of a moroccan center. *Brain and behavior* 2019; **9**(1): e01177.

277. Zouhair K, El Ouazzani T, El Omari K, El Fajri S, Lakhdar H. Vulvar pathology. [French]. *Eastern Mediterranean Health Journal* 2002; **8**(6): 812-8.

278. Khan I, Rizvi K, Javed M, Shah SH. Pattern of sexually transmitted diseases in Hazara Division. *Journal of Ayub Medical College Abbottabad* 1995; **7**(2): 28-9.

279. Rehan N. Profile of men suffering from sexually transmitted infections in Pakistan. *Journal of Ayub Medical College Abbottabad* 2003; **15**(2).

280. Sami S, Baloch SN. Vaginitis and sexually transmitted infections in a hospital based study. *JPMA The Journal of the Pakistan Medical Association* 2005; **55**(6): 242-4.

281. Alsuhaibani O, Pereira WC, Tareeqanwar M, et al. Infectious disease screening among stem cell transplant donors: An Institutional experience in Saudi Arabia. *Annals of Neurosciences* 2015; **22**(2): 81-6.

282. Saxena AK, Panhotra BR, Naguib M, Uzzaman W, Al MK. Nosocomial transmission of syphilis during haemodialysis in a developing country. *Scand J Infect Dis* 2002; **34**(2): 88-92.

283. Doufik J, Zemmama H, Bouri S, et al. Prevalence of sexually transmitted infections in patients with schizophrenia in Morocco. *Infectious Diseases Now* 2022; **52(5)**: 304-5.

284. Ruge H. Serological findings in leprosy and tuberculosis with the Wassermann, Meinicke, and VDRL tests. *Bull World Health Organ* 1955; **13**(5): 861-86.

285. Sokrab TEO, Sid-Ahmed FM, Idris MNA. Acute stroke type, risk factors, and early outcome in a developing country: A view from Sudan using a hospital-based sample. *Journal of Stroke and Cerebrovascular Diseases* 2002; **11**(2): 63-5.

286. Hasan AA, Hamad SS, Salman YJ. Seropositivity for toxoplasmosis and other protozoan infections in patients with ocular diseases in Kirkuk province, Iraq. *Journal of Population Therapeutics and Clinical Pharmacology* 2023; **30**(9): e61-e71.

287. Batool T, Nawab S, Mehmood B, Younas NS, Khan MI, Nadeem K. The Analysis of Transfusion Transmitted Infections (TTIs) in Thalassemia Patients. *Pakistan Journal of Medical and Health Sciences* 2022; **16(2)**: 269-71.

288. Ali T, Schved JF. Registry of Hemophilia and other bleeding disorders in Syria. *Haemophilia* 2012; **18**(6): 851-4.

289. Abdo NM, Aslam I, Irfan S, et al. Seroepidemiology of Treponema pallidum, Mycoplasma hominis, and Ureaplasma urealyticum in fertility treatment-seeking patients in the Emirate of Abu Dhabi, United Arab Emirates. *Journal of Infection and Public Health* 2024; **17**(1): 163-71.

290. Waheed U, Abbas Zaheer H. Blood donor deferral pattern in Islamabad, Pakistan. *Vox Sanguinis* 2017; **112 (Supplement 2)**: 68.

291. el-Hassan AM, Wasfi A. Cardiovascular disease in Khartoum. Post-mortem and clinical evidence. *Tropical and geographical medicine* 1972; **24**(2): 118-23.

292. Wanni NHO, Al Dossary R, Obeid OE, et al. Seropositivity of syphilis among individuals screened in a tertiary hospital in the Eastern Province of Saudi Arabia. *Annals of Saudi Medicine* 2021; **41(1)**: 8-13.

293. Scott DA, Corwin AL, Constantine NT, et al. Low prevalence of human immunodeficiency virus-1 (HIV-1), HIV-2, and human T cell lymphotropic virus-1 infection in Somalia. *American Journal of Tropical Medicine and Hygiene* 1991; **45**(6): 653-9.

294. McCarthy MC, Burans JP, Constantine NT, et al. Hepatitis B and HIV in Sudan: A serosurvey for hepatitis B and human immunodeficiency virus antibodies among sexually active heterosexuals. *American Journal of Tropical Medicine and Hygiene* 1989; **41**(6): 726-31.

295. Mailloux M. Serologic syphilis reactions in the Tangiers region. [French]. *Maroc medical* 1971; **51**(544): 172-3.

296. World Health Organization. Global Health Observatory data repository. Available at: <https://apps.who.int/gho/data/node.main.A1357STI?lang=en>. Accessed June 7, 2023.

297. Chemaitelly H, Weiss HA, Smolak A, Majed E, Abu-Raddad LJ. Epidemiology of Treponema pallidum, Chlamydia trachomatis, Neisseria gonorrhoeae, Trichomonas vaginalis, and herpes simplex virus type 2 among female sex workers in the Middle East and North Africa: systematic review and meta-analytics. *J Glob Health* 2019; **9**(2): 020408.

298. Smolak A, Chemaitelly H, Hermez JG, Low N, Abu-Raddad LJ. Epidemiology of Chlamydia trachomatis in the Middle East and north Africa: a systematic review, meta-analysis, and meta-regression. *Lancet Glob Health* 2019; **7**(9): e1197-e225.
